# Genome evolution across 1,011 *Saccharomyces cerevisiae* isolates

**DOI:** 10.1038/s41586-018-0030-5

**Published:** 2018-04-11

**Authors:** Jackson Peter, Matteo De Chiara, Anne Friedrich, Jia-Xing Yue, David Pflieger, Anders Bergström, Anastasie Sigwalt, Benjamin Barre, Kelle Freel, Agnès Llored, Corinne Cruaud, Karine Labadie, Jean-Marc Aury, Benjamin Istace, Kevin Lebrigand, Pascal Barbry, Stefan Engelen, Arnaud Lemainque, Patrick Wincker, Gianni Liti, Joseph Schacherer

**Affiliations:** 1 Université de Strasbourg, CNRS, GMGM UMR 7156, Strasbourg, France; 2 Université Côte d’Azur, CNRS, INSERM, IRCAN, Nice, France; 3 Commissariat à l’Energie Atomique (CEA), Genoscope, Institut de Biologie François-Jacob, Evry, France; 4 Université Côte d’Azur, CNRS, IPMC, Sophia Antipolis, Valbonne, France; 5 CNRS UMR 8030, Université d’Evry Val d’Essonne, Evry, France

**Keywords:** Genome evolution, Rare variants

## Abstract

Large-scale population genomic surveys are essential to explore the phenotypic diversity of natural populations. Here we report the whole-genome sequencing and phenotyping of 1,011 *Saccharomyces cerevisiae* isolates, which together provide an accurate evolutionary picture of the genomic variants that shape the species-wide phenotypic landscape of this yeast. Genomic analyses support a single ‘out-of-China’ origin for this species, followed by several independent domestication events. Although domesticated isolates exhibit high variation in ploidy, aneuploidy and genome content, genome evolution in wild isolates is mainly driven by the accumulation of single nucleotide polymorphisms. A common feature is the extensive loss of heterozygosity, which represents an essential source of inter-individual variation in this mainly asexual species. Most of the single nucleotide polymorphisms, including experimentally identified functional polymorphisms, are present at very low frequencies. The largest numbers of variants identified by genome-wide association are copy-number changes, which have a greater phenotypic effect than do single nucleotide polymorphisms. This resource will guide future population genomics and genotype–phenotype studies in this classic model system.

## Abstract (web summary)

Whole-genome sequencing of 1,011 natural isolates of the yeast Saccharomyces cerevisiae reveals its evolutionary history, including a single out-of-China origin and multiple domestication events, and provides a framework for genotype–phenotype studies in this model organism.

## Main

The budding yeast *S. cerevisiae* is a powerful model system for understanding eukaryotic biology at the cellular, molecular and genomic levels^1,2^. *S. cerevisiae* has recently emerged as a model in population genomics^3–5^, because it can be found worldwide in a broad array of human-associated (for example, wine, sake, beer and other fermented beverages) and wild (for example, plant, soil and insect) biotopes. Recent years have seen a spike in the number of published *S. cerevisiae* genome sequences, which together have revealed a high level of genetic diversity and a complex population structure in this yeast^6–12^. However, the number of available sequenced genomes from natural isolates remains limited and stands in contrast to the wealth of data on *Arabidopsis thaliana*^13^ and humans^14,15^; this small sample size for yeast genomes has not fully captured the global evolutionary processes relevant to the species. Here we apply deep coverage genome sequencing to more than 1,000 natural *S. cerevisiae* isolates and explore their phenotypic landscape. To our knowledge, our large-scale genome analysis provides the first comprehensive view of genome evolution at different levels (for example, accounting for differences among ploidy, aneuploidy, genetic variants, hybridization and introgressions), which is challenging to obtain at this scale and accuracy for other model organisms. This sequencing effort substantiates previous hypotheses but also reveals novel aspects of *S. cerevisiae* evolutionary history. Natural *S. cerevisiae* isolates have previously proven to be a powerful tool for investigating the genotype–phenotype relationship via linkage mapping^16^. Although genome-wide association studies (GWAS) have led to the identification of common alleles with strong effects in other organisms, the small sample size of yeast genomes has so far prevented similar attempts for yeast. Our dataset enables GWAS and exhaustively captures genetic variants (single nucleotide polymorphisms (SNPs), copy-number variants (CNVs) and genome content), providing insights into the genetic architecture of traits and the source of missing heritability.

## Species-wide genetic and phenotypic diversity

We assembled a collection of 1,011 *S. cerevisiae* isolates that maximized the breadth of their ecological and geographical origins (Supplementary Fig. 1a, b and Supplementary Table 1). We deeply sequenced 918 isolates using the Illumina paired-end strategy with a 232-fold mean coverage, and we also included 93 strains that had previously been sequenced^6–8^ (Supplementary Fig. 1c). The reads associated with each sample were mapped to the S288C reference genome and de novo assembled. A total of 1,625,809 high-quality reference-based SNPs were detected across the 1,011 genomes. Most of these SNPs are present at very low frequencies, with 31.3% of the polymorphic positions being singletons and 93% with a minor allele frequency (MAF) < 0.1 (Supplementary Fig. 2). This bias might in part be driven by the sampling scheme. Deleterious mutations as predicted by SIFT^17^ show the strongest bias toward rare alleles, consistent with the notion that selection prevents such SNPs from spreading in the population (Supplementary Fig. 2). In addition, we detected 125,701 small-scale insertions and deletions (indels) (up to 50 bp) with the majority exhibiting a low frequency (Supplementary Fig. 3a). Most indels are short (42.6% are 1 bp in length) and those present in coding regions are strongly biased to lengths that are multiples of three, which reflects the influence of purifying selection (Supplementary Fig. 3b, c). We also characterized CNVs by measuring the coverage ratio of each individual pangenomic open reading frame (ORF) (see below) normalized to the genome of each respective strain (Methods). CNVs are heavily enriched in subtelomeric regions, whereas internal chromosomal regions are largely copy-number stable (Supplementary Fig. 4a). Nearly all ORFs have at least one strain with a CNV. Most CNVs associated with individual ORFs are rare in the population (Supplementary Fig. 4b). Variants with high copy numbers affect only a small fraction of ORFs (Supplementary Fig. 4c) and extreme cases (> 20 copies) include 2μ plasmid ORFs, mitochondrial genome, ribosomal DNA and repetitive elements such as Ty and Y′.

In parallel, 971 strains were phenotyped in different conditions that affect various physiological and cellular responses (Methods and Supplementary Table 2). In total, we analysed 34,956 phenotypic measurements that covered 36 traits, providing a comprehensive analysis of their inheritance patterns. Most of the traits vary continuously across the population in a manner consistent with their genetic complexity (Supplementary Fig. 5). However, some traits—such as resistance to copper sulfate (CuSO_4_) or anisomycin^18^—follow a bimodal distribution model, and therefore a Mendelian inheritance pattern. Estimates of the narrow-sense heritability, *h*^2^, from genome-wide SNPs genotyped^19^ show a substantial amount of variance explained across all the traits, with a mean of 0.69, which suggests the feasibility of performing GWAS (Supplementary Fig. 6).

## Population structure supports out-of-China origin

**Fig. 1:**
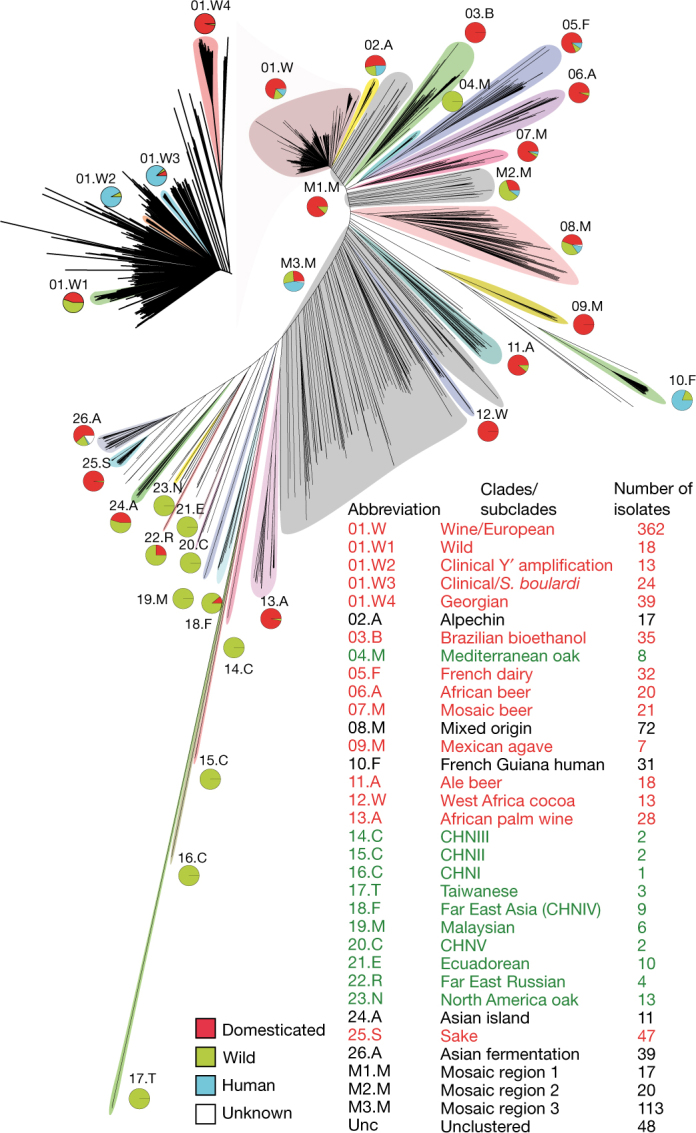
Neighbour-joining tree built using the biallelic SNPs. We identified 26 clades (numbered clockwise from 1 to 26) and three mosaic groups (M1–M3). The pie charts represent the ecological origins of the clade: domesticated (red), wild (green) and human (cyan). The colour of the clade name indicates its assignment: domesticated (red) and wild (green). The top left inset represents a magnification of the wine/European clade with four major subclades highlighted.

The phylogenetic tree of the 1,011 strains shows well-defined clades, loose clusters and isolated branches (Fig. 1). Most of the strains (813 in total) fall into 26 clades, and another 150 strains belong to three groups of poorly related strains (Supplementary Information note 1). Our data revealed a complex pattern of genetic differentiation with distinct lineages that correlate with geography, environmental niche and the degree of human association, as has previously been reported^8,9,11,12,20^. Domesticated and wild clades largely fall into two well-delineated sides of the tree, and are separated by a large group of mosaic strains. The main exceptions are the wild Mediterranean oak strains, which group with the domesticated clades, and the sake strains, which group with wild clades. However, the Mediterranean oak lineage groups together with the other wild lineages on the basis of ORF-content strain clustering (Supplementary Fig. 7).

We used ADMIXTURE^21^ to investigate ancestry in the genomes of individual strains. Mosaic strains are characterized by admixture from two or more lineages derived by outbreeding^3,4^ and frequently manifest as isolated branches in the phylogenetic tree. We identified three groups of mosaic strains that are mostly associated with human-related environments (Fig. 1). Population structure analysis revealed different sources of ancestry and degrees of mosaicism, consistent with multiple hybridization events (Supplementary Fig. 8). These findings underscore the role of human-driven admixture in shaping the population structure of *S. cerevisiae*.

**Fig. 2:**
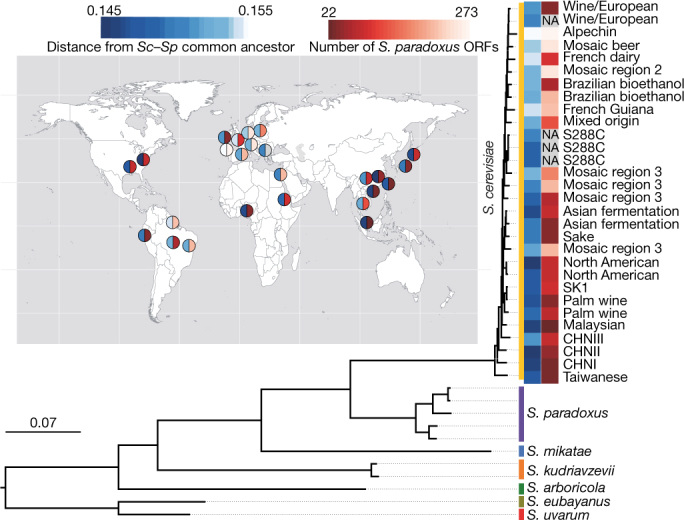
Chinese origin of *S. cerevisiae*. Maximum-likelihood rooted tree of the *Saccharomyces* complex, based on the alignment of 2,018 concatenated conserved genes. Heat maps display the distance from the last common ancestor of *S. cerevisiae* (*Sc*)–*S. paradoxus* (*Sp*) (white–blue), and the number of introgressed *S. paradoxus* ORFs (white–red). The map shows the geographical origins of the strains.

The recent discovery of highly diverged wild Chinese lineages suggests that East Asia may represent the geographic origin of *S. cerevisiae*^22^. The Taiwanese wild lineage represents the most divergent population that has yet been described (average of 1.1% sequence divergence to non-Taiwanese strains). This lineage also contains an extremely divergent 2μ plasmid that shares only 80% of identity with known plasmid variants (Supplementary Fig. 9 and Supplementary Information note 2). We used a subset of highly contiguous de novo assemblies that sample the main *S. cerevisiae* lineages and closely related *Saccharomyces* species^23,24^ to generate a rooted phylogenetic tree (Fig. 2). The outgroup species branched off near the Taiwanese and Chinese lineages, which strongly supports a Chinese origin for *S. cerevisiae*. This scenario is also consistent with the isolation of closely related *Saccharomyces* species such as *S. mikatae* and *S. arboricola*^25,26^, which are restricted to East Asia, and the broad genetic diversity of the Japanese *S. kudriavzevii* populations^27^. Together, these observations suggest an Asian origin for the whole *Saccharomyces* species complex. We then tested the number of out-of-China events by investigating the relationship of non-Chinese strains to the genetic structure of Chinese strains. We performed a principal component analysis on SNPs that included only the CHN I–V (Chinese isolates) and Taiwanese strains and then projected the rest of the strains onto the principal component space defined by these highly diverged lineages (Supplementary Fig. 10). Principal component 1 defines the separation of the Taiwanese strains from all other strains, consistent with the deep genetic differentiation of this lineage. Principal components 2–4 then define differentiation between different Chinese lineages. Notably, the non-Chinese strains all project onto the same part of the space, which implies that they are essentially all equally related to the different Chinese lineages. In other words, it appears that different non-Chinese strains do not derive from different Chinese lineages but instead share a single out-of-China origin.

## Ploidy and aneuploidy variation by ecological origin

**Fig. 3:**
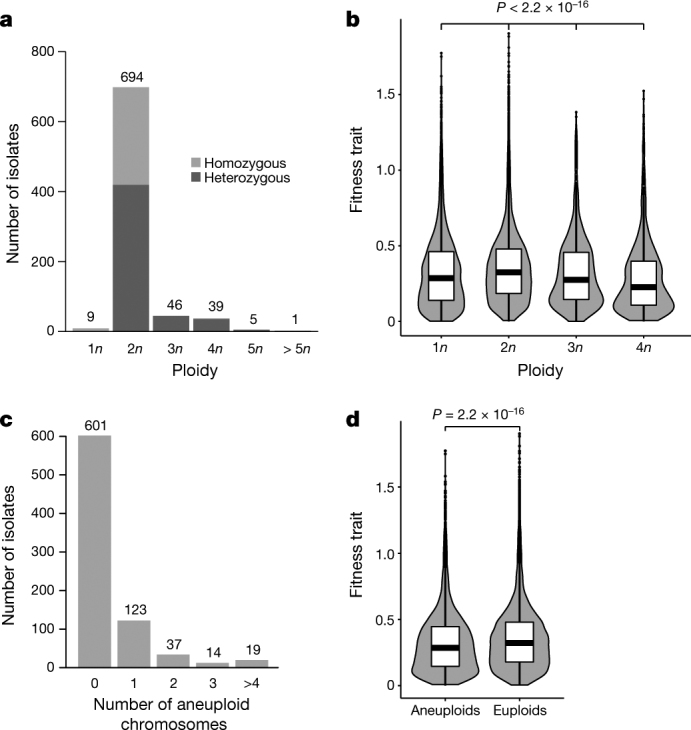
Ploidy and aneuploidy natural variation. **a**, Distribution of ploidy and fraction of heterozygous isolates. **b**, Violin plot of growth fitness trait by ploidy. Diploid isolates are globally fitter than individuals with other ploidy levels. Number of trait values for 1*n* isolates = 4,585; for 2*n* isolates = 26,249; for  3*n* isolates = 1,610; and for  4*n* isolates = 1,330. **c**, Distribution of aneuploid chromosomes per individual. **d**, Violin plot of growth fitness trait of aneuploid (*n* = 6,510) and euploid (*n* = 20,719) isolates shows a significant difference in fitness trait between the two categories. All *P* values were calculated using a two-sided Mann–Whitney–Wilcoxon test. Centre lines, median; boxes, interquartile range (IQR); whiskers, 1.5 × IQR. Data points beyond the whiskers are outliers.

Variation in ploidy and aneuploidy is not uncommon in yeast, and this genomic plasticity is often described as a strategy for rapid adaptation to environmental changes^28–30^. As 217 strains were genetically manipulated and no longer in their natural ploidy states, we assigned a relative ploidy state to the other 794 isolates (Methods), and found 9 haploids, 694 diploids and 91 isolates with higher ploidy (Fig. 3a). Our results reveal that most (about 87%) of the natural isolates are in a diploid state. Polyploid isolates (3–5*n*) are not frequent (approximately 11.5%) and enriched in specific subpopulations such as the beer, mixed-origin and African palm wine clades, which strongly suggests that some human-related environments have had an effect on the ploidy level (Supplementary Fig. 11). By testing the effect of ploidy on the growth fitness trait across the species, a general advantage was observed for diploid isolates compared to other types of ploidy (Fig. 3b). This result supports a general mitotic growth advantage for diploidy in yeast^30,31^ that holds across every condition tested, and shows no major environment-specific effects (Supplementary Fig. 12).

By combining coverage analysis and allele frequency distributions, we determined the copy number of each chromosome together with instances of segmental duplications (Fig. 3c, Supplementary Fig. 13a and Supplementary Table 1). We identified a total of 342 cases of aneuploidy that affected 193 isolates (19.1% of the 1,011 strains). The most frequently observed cases of aneuploidy involve chromosomes I, III and IX and are only weakly correlated with chromosome size (Supplementary Fig. 13b). There is a strong enrichment of aneuploid strains in the sake, ale beer and mixed-origin clades (Supplementary Fig. 14). Aneuploidy is therefore not uncommon but its relationship with fitness is paradoxical. Indeed, cases of aneuploidy arise under a variety of selective regimes but lead to a decrease of global cellular fitness^32–34^. We found a general mitotic growth advantage in euploid versus aneuploid strains, consistent with a fitness cost for chromosomal aneuploidy (Fig. 3d).

## A portrait of the *S. cerevisiae* pangenome

**Fig. 4:**
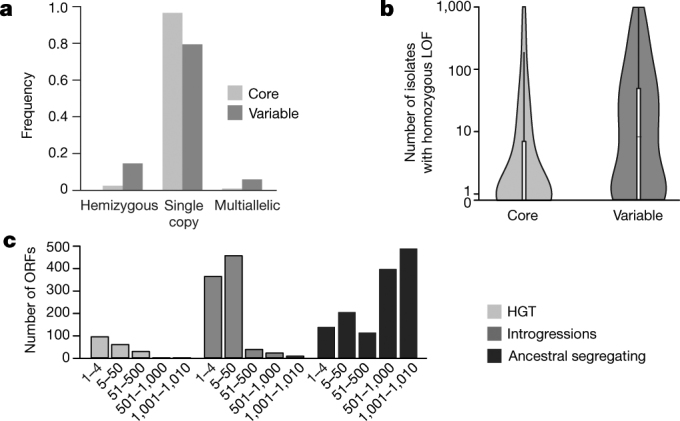
The *S. cerevisiae* pangenome. **a**, Copy number distribution for core and variable ORFs. Variable ORFs have a greater frequency of both hemizygous and multiallelic genes. **b**, Logarithmic-scale distribution of isolates carrying loss-of-function mutations for core (*n* = 4,931) and variable ORFs (*n* = 1,111). The core genome is characterized by far fewer loss-of-function mutations compared to variable ORFs (*P* value = 6.45 × 10^−78^, two-sided Mann–Whitney–Wilcoxon test). Centre lines, median; boxes, IQR; whiskers, 1.5 × IQR. Data points beyond the whiskers are outliers. **c**, Different types of variable ORFs have marked differences in distribution.

The 1,011 genomes provided an opportunity to determine the yeast pangenome^35^ using de novo genome assemblies and detection of non-reference material (Methods). Containing a total of 7,796 ORFs, the pangenome (Supplementary Table 3) is composed of 4,940 core ORFs and 2,856 ORFs that are variable within the population. Most core ORFs are present as a single copy per haploid genome, whereas variable ORFs show a higher frequency of both hemizygous and multi-allelic copy numbers (Fig. 4a). Analysis of the 6,081 non-redundant ORFs present in the well-annotated S288C reference genome (4,937 core and 1,144 variable ORFs) highlighted different trends. First, the distribution of variable ORFs is biased towards subtelomeric regions (Supplementary Fig. 15a), known as hotspots of gene content variation^7,24,36^, and shows a strong functional enrichment for cell–cell interactions, secondary metabolisms and stress responses (Supplementary Table 4). Second, the core genome is characterized by lower levels of loss-of-function mutations and has a different ratio of substitution rates (ratio of nonsynonymous to synonymous polymorphisms, *d*_N_/*d*_S_), which reflects differences in selective constraints (Fig. 4b and Supplementary Fig. 15b). Out of the 1,072 essential genes defined in the S288C background, 123 do not belong to the core genome, although the absence of 71 of these is complemented by closely related orthologues (Supplementary Table 5).

To trace the origins of the variable ORFs, we implemented a phylogenetic approach by inspecting the evolution of each individual ORF (Methods and Supplementary Fig. 16). We defined 1,380 ancestral segregating ORFs with sequence-similarity levels consistent with genome-wide species relatedness. We identified 913 introgressed ORFs, with a clear majority (*n* = 885) being unambiguously traced to a *Saccharomyces paradoxus* origin. All *S. cerevisiae* isolates carry at least one *S. paradoxus* ORF (median of 26), indicating ubiquitous gene flow between these two closely related species^37^. We also detected 14 ORFs in the Taiwanese lineage that were introgressed from an undocumented species (Supplementary Fig. 17). The amount of introgressed content is highly variable, with an enrichment in four human-associated subpopulations in which two species might coexist and which might represent interspecific hybrid zones (Supplementary Fig. 18 and Supplementary Information note 3). By contrast, introgressions are rare in the highly diverged lineages, consistent with secondary contacts with *S. paradoxus* occurring mainly after the out-of-China dispersal (Fig. 2). There is a notable match between the geographic origins of the *S. cerevisiae* clades and the ancestry of introgressed ORFs from *S. paradoxus* (Supplementary Fig. 19 and Supplementary Information note 3).

Finally, 183 ORFs are likely to be the result of horizontal gene transfer (HGT) events from highly divergent yeast species. Introgressed ORFs tend to replace *S. cerevisiae* orthologues, which suggests that they are integrated by homologous recombination; by contrast, HGT segments localize mainly at subtelomeres. We traced the donor for 85 HGT ORFs and found an enrichment for *Torulaspora* and *Zygosaccharomyces* species. These species coexist with *S. cerevisiae* in fermentative environments, which might promote HGT events or favour their retention. We identified 6 large (38–165 kb) HGT events with most isolates retaining only small segments in complex patterns, consistent with multiple independent rearrangements leading to partial deletions of the large ancestral HGT (Supplementary Information note 3, Supplementary Figs. 20–22 and Supplementary Table 6). Together, these introgression and HGT events with distinct population frequency distributions (Fig. 4c) correspond to important evolutionary processes that have shaped the *S. cerevisiae* species genome.

## Patterns of extensive loss-of-heterozygosity

**Fig. 5:**
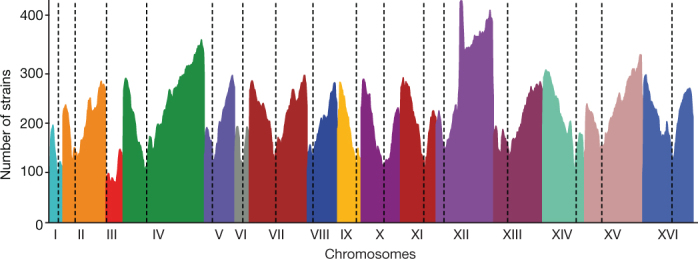
Landscape of loss-of-heterozygosity events. Density of the regions under LOH across the 16 nuclear chromosomes (I–XVI) within our population. Each colour corresponds to a chromosome, and centromere locations are represented by dotted lines.

*S. cerevisiae* is highly inbred and characterized by rare sexual cycles^38–40^. The frequency of outcrossing has considerable effects on genome variation, and particularly on patterns of heterozygosity. Among the 794 natural isolates, 505 isolates (about 63%) are heterozygous (Fig. 3a), with the proportion varying across subpopulations and with marked differences between domesticated and wild clades (Supplementary Fig. 23), as has previously been observed^41^. Heterozygous sites were spread across the genomes, but we also detected large regions of loss-of-heterozygosity (LOH) and generated an accurate genome-wide LOH map (Fig. 5, Methods and Supplementary Fig. 24a). LOH events range from 2 to 56 regions per strain and represent up to 80% of the sake isolate genomes (Supplementary Fig. 24b). Although LOH levels are variable across subpopulations (Supplementary Fig. 25), we observed an overall high LOH level with 25 regions covering approximately 50% of the genome on average (Supplementary Fig. 24b, c and Supplementary Table 7). However, LOH events are not evenly distributed along the genome (Fig. 5) and centromere-proximal regions exhibit low levels of recombination initiation, consistent with them experiencing few LOH events.

By masking the LOH regions we precisely determined the level of heterozygosity, which ranges from 0.63 to 6.56 heterozygous sites per kb (Supplementary Table 8). The distribution of the level of heterozygosity in the population is bimodal, reflecting the variability observed across subpopulations (Supplementary Fig. 26). These observed patterns are likely to be related to variation in the rate of outcrossing rate, as LOH and heterozygosity correlate with one another (Supplementary Fig. 27). The prevalence of LOH events agrees with the low outcrossing rate^38^ and most LOH is thought to be the result of mitotic recombination^42^ or cells returning to mitotic growth after entering the meiotic phase^43,44^. Overall, our data support the idea that *S. cerevisiae* undergoes clonal expansion followed by LOH-mediated diversification, enabling the expression of recessive alleles and generating novel allele combinations with potential effects on phenotypic diversity.

## Genetic diversity and evolution by subpopulation

The comparison of genome content variation and levels of SNPs in domesticated and wild clades (Supplementary Fig. 28) shows higher SNP density (median 0.55% versus 0.41%) and lower genome content variation (median 115 ORFs that are not shared, versus 161 shared ORFs) in wild versus domesticated clades, respectively (Supplementary Fig. 28 and Supplementary Tables 9, 10). These findings suggest a shift in evolutionary mechanisms during the domestication process. The wild clades share similar genome content, and their evolution is mainly driven by the accumulation of SNPs. The specific artificial environments colonized by domesticated clades probably promote rapid ORF expansion and/or loss, leading to considerable variation in genome content and CNVs. Some domesticated clades also exhibit high copy numbers of Ty1 and Ty2 transposable elements (Supplementary Fig. 29).

We investigated evolutionary patterns across multiple *S. cerevisiae* subpopulations using 19 clades that contained at least 10 isolates. By determining the ploidy and measuring the genome-wide levels of genetic diversity (*π* and *θ*_w_), the MAF spectrum and Tajima’s *D* values, our results revealed distinct evolutionary histories across subpopulations (Supplementary Table 11). We also estimated the timings of the *S. cerevisiae* out-of-China and domestication events (Supplementary Information note 4).

Analyses of human-related *S. cerevisiae* subpopulations provide strong evidence for various independent and lineage-specific domestication events. Although *S. cerevisiae* beer isolates are polyphyletic, they are characterized by higher ploidy (≥3*n*) and a higher number of aneuploidy events than other domesticated lineages. In addition, the genetic diversity of beer isolates is very high (*π* = 2.8 × 10^−3^ on average) and the number of heterozygous sites they possess is elevated (ranging from 17,807 to 34,203 heterozygous sites on average). Finally, LOH regions represent a small proportion of the beer genomes (11% on average). The independent but convergent domestication processes undergone by beer isolates are, therefore, marked by genome-level modification and high nucleotide variation.

By contrast, wine and sake isolates are primarily diploids, monophyletic and have limited genetic diversity (*π* values of 1 × 10^−3^ and 0.8 × 10^−3^, respectively). The wine cluster is characterized by a strong bias towards low-frequency polymorphisms, with more than 95% of the polymorphic sites with MAF < 0.1. In addition, wine isolates harbour low heterozygosity and extensive LOH, which suggests a low outcrossing rate. All of these observations indicate that wine isolates experienced a population expansion after a domestication bottleneck. The effect of the domestication event on the sake genomes is very similar. Indeed, these genomes have low levels of genetic diversity, low heterozygosity and extensive LOH regions. However, the sake subpopulation does not exhibit a bias towards low-frequency alleles (Tajima’s *D* value of 0.0481), reflecting their more recent origin (Supplementary Information note 1)^45^.

## New insights into the genotype–phenotype relationship

Natural *S. cerevisiae* isolates have been a powerful tool for linkage mapping, revealing an large number of quantitative trait loci, over 100 quantitative trait genes and 50 quantitative trait nucleotides^16^. We investigated the allele frequency associated with these polymorphisms, which underlie quantitative trait variation (Supplementary Table 12). Among 36 well-characterized quantitative trait nucleotides, 24 were found with at a frequency of lower than 5%, and were therefore undetectable using a GWAS strategy. This strong bias towards rare alleles for these polymorphims is consistent with the overall MAF spectrum. Consequently, this result highlights that a fraction of the missing heritability of complex traits might be explained by rare variants in species with an allele frequency distribution similar to that of yeast.

**Fig. 6:**
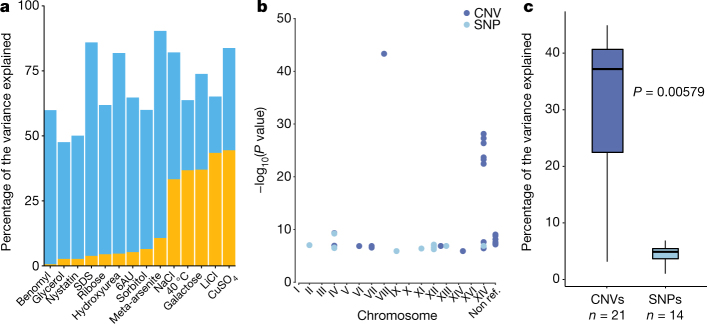
Genotype–phenotype relationship in *S. cerevisiae*. **a**, Narrow-sense heritability (blue) and phenotypic variance explained (yellow) for phenotypes with associated variants. **b**, Association scores of the detected genetic variants across the 16 chromosomes and the non-reference ORFs. **c**, Variance explained by CNVs and SNPs associated with traits. Association scores and variance explained are higher for CNVs compared to SNPs (*P* value = 0.00579, two-sided Mann–Whitney–Wilcoxon test). Centre lines, median; boxes, IQR; whiskers, 1.5 × IQR. Data points beyond the whiskers are outliers.

The high genetic diversity (*π* = 3 × 10^−3^) and low linkage disequilibrium (LD_1/2_ = 500 bp) (Supplementary Fig. 30) among *S. cerevisiae* isolates indicates that this species could represent a powerful resource for GWAS. We built a matrix of genetic variants that included comprehensive sets of SNPs, CNVs and non-reference variable ORFs. Our matrix contains a total of 82,869 SNPs and 925 CNVs, which represents a dense map with, on average, one marker every 143 bp. We estimated genome-wide phenotype heritability^19^ and revealed that traits are not stratified by subpopulations (Fig. 6a, Supplementary Figs. 31, 32 and Supplementary Tables 13, 14).

We performed a mixed-model association^19^ and detected 35 genetic variants associated with 14 conditions (22 CNVs versus 13 SNPs), with an enrichment and high association scores for CNVs (Fig. 6b, Supplementary Fig. 33 and Supplementary Table 15). In addition, four of the variants we detected are linked to non-reference ORFs. For five traits, the phenotypic variation explained is greater than 25% (Fig. 6a). CNVs explained larger proportions of trait variance compared to SNPs (a median of 36.8% and 4.49% of the variance explained, respectively; Fig. 6c). As an example, we found the *CUP1* gene strongly associated with resistance to copper sulfate (*P* value = 4.85 × 10^−44^) (Supplementary Fig. 33 and Supplementary Table 15). Amplification of this locus strongly contributes to the resistance to high concentrations of copper and cadmium^46^ with copy number variation alone explaining 44.5% of phenotypic variation. Our GWAS analyses, which included an exhaustive catalogue of genome content and CNVs, highlighted the overall importance of these genetic variants for phenotypic diversity. The high number of associated CNVs is consistent with the notion that these variants can contribute to a large amount of phenotypic variation^47^.

## Conclusion

Our whole-genome sequencing of 1,011 isolates, combined with our phenotyping efforts, provides a detailed view of *S. cerevisiae* variation. This resource has revealed previously undescribed evolutionary history as well as the driving forces of genome evolution, and has provided insights into the genotype–phenotype relationship. Our study lays the foundation for GWAS in *S. cerevisiae* and provides a population genomic resource at a scale that matches those of other model organisms^13,14^. The difference between the estimated genome-wide heritability and explained phenotypic variance gives an overview of the extent of missing heritability^48,49^. Many SNPs are present at low frequencies, which echoes observations previously made in human GWAS^15^ and raises the question of whether rare SNPs have an important role in modulating the phenotypic landscape. Furthermore, the comprehensive characterization of the species pangenome can further improve the genotype-to-phenotype map. The availability of end-to-end genome assemblies will enable the organization of such a dataset with genome graphs^50^. This collection of genetic and phenotypic variants will therefore enable novel functional approaches in a powerful model system.

## Methods

### *S. cerevisiae* sequenced isolates

The isolates included in this project were carefully selected to be representative of the *S. cerevisiae* whole species. All the isolate details, including ecological and geographical origins, providers and references, are listed in Supplementary Table 1. We maximized the isolate ecological origins by including both human-associated environments such as wine and sake fermentation, brewing and dairy products, as well as natural environments such as soil, insects, tree exudate and fruit. Geographical origins are also highly diverse and have a worldwide distribution (Supplementary Table 1). In addition to the 918 isolates provided by research laboratories and yeast collections, we included 93 strains sequenced in previous studies^6–8^, to give a total of 1,011 samples analysed in this study. We sought to keep the isolates in their natural state before sequencing to provide a global picture of the ploidy and level of heterozogosity. However, among the 918 selected isolates, 124 were non-natural haploid with the *HO* gene deleted and the 93 external isolates were genetically manipulated and made homozygous before sequencing.

### Sequencing and quality filtering

Yeast cell cultures were grown overnight at 30 °C in 15 ml of YPD medium to early stationary phase. Total genomic DNA was subsequently extracted using MasterPure Yeast DNA Purification Kit and Genomic Illumina HiSeq 2000 sequencing libraries were prepared for 918 strains with an insert size between 300 and 600 bp. Ten libraries were multiplexed per Illumina HiSeq2000 lane and subjected to paired-end sequencing, producing reads of 102 bases.

An in-house quality control process was applied to the reads that passed the Illumina quality filters. Illumina sequencing adapters and primers sequences were removed from the reads and the low-quality nucleotides (*Q < *20) were discarded from both ends of the reads. Reads shorter than 30 nucleotides after trimming were removed. These trimming and removal steps were achieved using software designed in-house, based on the FastX package. The last step identifies and discards read pairs that mapped to the phage phiX genome (GenBank code: NC_001422.1) using SOAP^51^. A total of 3.35 Tb of high-quality genomic sequence was generated with a mean sequencing depth of 232 × per isolate (ranging from 50 × to 1,014 ×, Supplementary Fig. 1c). For the publically available Illumina paired-end reads related to 93 strains (see ‘*S. cerevisiae* sequenced isolates’), the mean sequencing depth is 169 × (from 20 × to 570 × , Supplementary Fig. 1c).

### Reads mapping and variant calling

For each isolate, the reads were mapped to the *S. cerevisiae* S288C reference genome (version R64-1-1) with Burrows–Wheeler Aligner (BWA v.0.7.4-r385)^52^, using default parameters. Duplicated reads were marked with Picard-tools (v.1.124) (http://picard.sourceforge.net) and local realignment around indels and variant calling were performed with GATK (v.3.3-0)^53^. Default parameters were applied except for the realignment step (GATK IndelRealigner), in which the following parameters were set: ‘–maxReadsForConsensuses 500–maxReadsForRealignment 40000–maxConsensuses 60–maxPositionalMoveAllowed 400–entropyThreshold 0.2’. GATK Variant Annotator was run to add allele balance information in the.vcf files.

### Ploidy, types of aneuploidy and segmental duplications

The natural ploidy of the 794 natural isolates (see ‘*S. cerevisiae* sequenced isolates’), as well as their aneuploidy and segmental duplication content were investigated by combining three complementary approaches:

First, measurement of the cell DNA content using high-throughput flow cytometry: DNA content was analysed using a propidium iodide (PI) staining assay. Cells were first pulled out from glycerol stocks in liquid YPD in 96-well plates (30 °C, overnight). Five microlitres of the culture were transferred into 195 μl of fresh YPD and incubated for 8 h at 30 °C. Then, 3 μl were taken and resuspended in 100 μl of cold 70% ethanol. Cells were fixed overnight at 4 °C, washed twice with PBS, resuspended in 100 μl of staining solution (15 μM PI, 100 μg/ml RNase A, 0.1% v/v Triton-X, in PBS) and finally incubated for 3 h at 37 °C in the dark. Ten thousand cells for each sample were analysed on a FACS-Calibur flow cytometer using the HTS module for processing 96-well plates. Cells were excited at 488 nM and fluorescence was collected with a FL2-A filter. The distributions of both FL2-A and FSC-H values have been processed to find the two main density peaks, which correspond to the two cell populations in G1 and G2. The peaks were detected using the densityClust R package after removing the cells reaching the FACS saturation (either FLS-A or FSC-H values equal to 1,000). We categorized the values of FLS-A, which correlate with the DNA quantity, to estimate the ploidy according to the following scheme: strains with G1 cell values between 39 and 181 and G2 values between 148 and 255 were labelled as haploid; strains with G1 cell values between 145 and 265 and G2 values between 295 and 500 were labelled as diploid; strains with G1 cell values between 245 and 355 and G2 values between 500 and 700 were labelled as triploid; strains with G1 cell values between 295 and 500 and G2 values between 700 and 905 were labelled as tetraploid; strains with G1 cell values between 395 and 605 and G2 values over 905 were labelled as over 4*n*; strains with other combinations of values have been manually evaluated.

Second, study of sequencing coverage: systematic analysis of the coverage depth along the genome was performed with 1-kb non-overlapping sliding windows, which enabled the survey of chromosomal copy number variations as well as segmental duplications. The ratio between the coverage of the aneuploid chromosomes and the rest of the genome was also used to validate the ploidy of isolates.

Third, investigation of the allele balance ratio associated with heterozygous SNPs, as heterozygous sites should fit an expected range of ratios according to the copy number of the chromosome being considered (see ‘SNPs filtering and matrix’).

The precise locations of segmental duplications were manually investigated in the .vcf files (Supplementary Table 16).

### SNP filtering and matrix

For each sample, variants were first called with GATK HaplotypeCaller (see ‘Reads mapping and variant calling’). At this stage, isolates with less than 5% of heterozygous sites (average percentage of heterozygous sites detected in a sample of 104 haploid and/or homozygous diploid isolates) were considered as homozygous. The raw files were then post-processed to deal with highly confident variants to be included in our complete SNPs matrix, based on both coverage and allele balance information:

First, a minimal coverage depth of 50 × was required for a SNP to be retained for the 918 isolates that were sequenced in this study; this coverage depth was lowered to 10 × for the other 93 previously sequenced isolates.

Second, for the haploid and homozygous isolates (< 5% of heterozygous sites), the fraction of heterozygous SNPs detected was considered as representing false positives and was therefore filtered out.

Third, for heterozygous isolates, heterozygous sites were filtered according to their allele balance ratio (ABHet). The thresholds for allele balance ratios were determined according to the allelic frequency distribution all over the heterozygous samples at each level of ploidy (from 2*n* to 5*n*). A heterozygous site was rejected when its ratio did not fit the expected range according to the copy number of the chromosome being considered (or region, in the case of segmental duplication).

The joint calling method of GATK was run with the cleaned .vcf files to create a complete genotyping matrix (.gvcf format, see ‘Data availability’). This matrix of SNPs included 1,625,809 segregating sites across our 1,011 isolates (Supplementary Table 1).

SnpEff (v.4.1)^54^ was used to annotate and predict the effect of the variants. Non-synonymous SNPs predicted as deleterious by SIFT (v.5.2.2)^17^ as well as nonsense mutations were considered as deleterious for protein function. Insertions and deletions were considered to cause frame shifts when their sum produced a number not divisible by three in a single gene.

A sequence representative of each isolate was constructed by inserting these filtered SNPs into the reference sequence with GATK FastaAlternateReferenceMaker (see ‘Data availability’).

### Pangenome

#### De novo genome assembly

We used Abyss software (v.1.5.2)^55^ with the option ‘-k 64’ to produce the de novo assemblies (see ‘Data availability’). The pre-assembly filtering step was performed with condetri v.2.2 to remove the 6 bases closest to the 5′ end and to discard low-quality 3′-end bases of reads. The resulting assemblies had a median N50 of 136 kb and a median number of contigs of 3,259. The median length of the genome is 12.1 Mb and the median GC content is 38.06 (Supplementary Table 17).

#### Detection of non-reference material

We set up a custom pipeline to identify non-reference genome material. Each genome was aligned to the reference sequence (S288C, version R64-1-1) using blastn with the following settings: ‘-gapopen 5 -gapextend 5 -penalty -5 -reward 1 -evalue 10 -word_size 11 -no_greedy’.

The CDH and CFH strains were excluded from the identification of non-reference genome material owing to the presence of *Staphylococcus epidermis* contamination. The sequences aligned with an identity greater than 95% were divided in three categories to be further processed. If the aligned sequence belonged to contigs shorter than 100 bp or if the aligned sequence was up to 200 bp and belonged to a contig with a length that was shorter than the length of the alignment plus 75 bp, the contig was discarded. If the aligned sequence was in the range 200–1,500 bp only the aligned sequence was discarded. If the aligned sequence was longer than 1,500 bp, it was divided into segments of 250 bp. Each sub-sequence was aligned again to the reference and discarded if found with an alignment identity of over 95% on an alignment length of at least 187 bp (75% of the subsequence length). After this step the relative position of the retained sequence has been evaluated. If two or more of them belonged to the same contig and were separated each other by less than 100 bp, the sequence from the starting of the first one to the end of the final one was kept as a whole. In the subsequent step, all the kept sequences from the 1,011 genomes were sorted for length in decreasing order. The set of sequence was then aligned against itself (with the same criteria as the first step) to eliminate repeated elements. When two sub-sequences were found to have an alignment identity of over 95%, the one belonging to the shorter sequence was eliminated. The process led to 12,325 sequences for 9.3 total Mb.

#### Annotation of non-reference material

To annotate ORFs in dispensable regions, we set up an integrative yeast gene annotation pipeline by combining different existing annotation approaches, which gave rise to an evidence-leveraged final annotation^24^. We independently ran the three individual components (RATT^56^, YGAP^57^ and MAKER^58^) for gene annotation, and subsequently integrated their results using EVM^59^. Proteomes of the *Saccharomyces* species (*S*. *cerevisiae*, *S*. *paradoxus*, *S*. *mikatae*, *S*. *kudriavzevii*, *S*. *arboricolus*, *S*. *uvarum* and *S*. *eubayanus*) were retrieved and used in our annotation pipeline to provide protein alignment support for annotated gene models^60,61^.

#### Pangenome definition

We compiled the pangenome by adding the 2,245 non-reference ORFs annotated here to the 6,713 genomic reference ORFs listed in the set ‘orf_genomic_all’ from the *Saccharomyces* Genome Database (SGD) database (updated 13 January 2015, https://downloads.yeastgenome.org/sequence/S288C_reference/orf_dna/). Three RDN genes were also added (*RDN18-1*, *RDN25-1* and *RDN37-1*) from the set ‘rna_genomic’ available from the SGD database (https://downloads.yeastgenome.org/sequence/S288C_reference/rna/). We applied a graph-based pipeline to this set of ORFs, to remove duplicate and closely related sequences. This step also removed overlapping ORFs present in the ‘orf_genomic_all’ SGD dataset. A disconnected graph was created in which each node is an ORF and each edge represents an alignment identity of over 95% on at least 75% of the sequence of the smaller ORF in the couple. Each connected subgraph represents a single ORF family. For each of these families a representative has been chosen. The connectivity has been computed for each node. The first choice for the representative was the most central, non-dubious reference ORF, if any of them were present. The second choice was the most central reference ORF; if only non-reference ORFs were present in the family, the most central of these was taken.

This led to a catalogue of 7,796 non-redundant ORFs (see ‘Data availability’), which represent the *S. cerevisiae* pangenome (Supplementary Table 3). Among the reference ORFs, the number of similar ORFs collapsed for each final ORFs has a wide range (up to 67, which is the cluster of Ty1 elements), although usually this number does not exceed 2. Other large clusters are the Y′ elements one (59 ORFs) and the Ty2 elements (27 ORFs). Out of the 6,713 reference ORFs, 5,679 were not redundant and 1,032 were collapsed into 402 unique ORFs; 89% of these unique ORFs (*n* = 357) are duplicated ORFs.

#### Pangenome CNVs

To assess the copy number of each ORF of the pangenome, we mapped the reads from each strain to the pangenomic ORFs with BWA,using default parameters and the option –U 0. The result was then filtered using samtools view with the options –bSq 20 –F 260. The median coverage for each ORF was taken as coverage for the ORF in the specific isolate. The ratio between the values of individual ORFs and the values of genome coverage on the reference of the isolate (as the median of the median coverage for each nuclear chromosome) was considered as the copy number for the haploid genome (see ‘Data availability’).

The mapping was also used as a confirmation step for the presence of the ORFs in each strain, leading to the identification of 4,940 ORFs present in the 1,011 strains of the collection, representing the core genome plus 2,856 ORFs present in different subsets of the population. Fifty-two ORFs were removed because they were present in single strains with low coverage (~10% of the genome wide coverage) and were likely to be contaminations from *Escherichia coli* and *Clavispora lusitaniae*. Eighty-nine other ORFs that did not have sufficient coverage were kept in the pangenome, but were not used for subsequent analyses owing to poor mapping. Three of the core ORFs are present but not annotated in the S288C reference and were annotated by our annotation pipeline as 584-snap_masked-1700-AIE_1, 610-snap_masked-2999-BGP_1 and 611-snap_masked-3001-BGP_1.

To evaluate the difference between domesticated clades and wild clades, we normalized the data by calculating the clade copy-number median for each ORF to avoid sample bias. The distributions of medians in the domesticated and wild clades were then compared using the Mann–Whitney*–*Wilcoxon test (R function wilcox.test) (Supplementary Fig. 28 and Supplementary Tables 18, 19).

#### Inference of pangenome origin

We constructed a local ORFs database for 57 representative species that deeply probed both closely related *Saccharomyces* species as well as a highly divergent yeast species^62^ (Supplementary Table 20). In addition, we added the ORFs of 12 representative *S. cerevisiae* and *S. paradoxus* strains with complete genome sequenced by long reads (PacBio)^24^. For each annotated variable ORF, we first performed a BLASTN search (‘-evalue 1E-6’) against this local ORFs database to find its best hit. ORFs without hits in our local yeast ORFs database underwent a further round of BLAST searching (-evalue 1E-6) against the NCBI non-redundant database (ftp://ftp.ncbi.nlm.nih.gov/blast/db/). Based on the sequence identity and query coverage of the top hits, we classified the variable genes into different categories.

#### d_N_/d_S_

For all isolates, sequences of the protein-coding genes were inferred from the filtered SNPs and inserted into the reference sequence with GATK FastaAlternateReferenceMaker. For each gene, the coding sequences were aligned and the ratio of nonsynonymous to synonymous polymorphisms (*d*_N_/*d*_S_) was computed with the yn00 program in PAML software^63^. Median values were used for comparison.

### Genomic diversity characterization

#### Genomic and genetic distances

The 1,544,489 biallelic segregating sites were used to construct a neighbour-joining tree (Fig. 1), using the R packages ape and SNPrelate. The .gvcf matrix was first converted into a .gds file and individual dissimilarities were estimated for each pair of individuals with the snpgdsDiss function. The bionj algorithm was then run on the distance matrix that was obtained.

The genomic content distance (see ‘Data availability’) has been calculated as the number of ORF differences in the pangenome presence/absence profile (that is, the number of ORFs present in only one strain for each pairwise strain comparison) (see ‘Data availability’).

#### Genetic diversity

As an estimate of the scaled mutation rate, we computed *π*, the average pairwise nucleotide diversity *θ*_w_, the proportion of segregating sites and Tajima’s *D* value, which represents the difference between *π* and *θ*_w_.

Variscan 2.0^64^ was run (runmode = 12, 10-kb non-overlapping windows) on multiple alignments of the concatenated chromosomes that were representative of the isolates.

#### Model-based ancestry

Model-based ancestry estimation was performed on the biallelic SNPs using ADMIXTURE v.1.23^21^ in unsupervised mode.

#### Principal component analysis

Principal components analysis on the biallelic SNPs was performed using EIGENSOFT v.6.0.1. The ‘-w’ argument was used to calculate the principal components using only a subset of the samples, with the remaining samples then being projected onto the resulting components.

#### Discriminant analysis of principal components

The matrix of presence/absence of ORFs in the population has been analysed using the discriminant analysis of principal components (DAPC) algorithm implemented in the R package adegenet 2.0.1. DAPC describes clusters by maximizing the between-cluster variance while minimizing the within-cluster variance. The number of components retained for the principal component analysis calculation was 150, accounting for > 88% of total variance. For the subsequent DAPC calculation, the alpha-score indicates 25 as the optimal number of discriminant principal components to be retained. Clustering was performed using the *K*-means with different number of groups (*n* = 5, 10, 15, 20, 25, 30, 35, 40, 45 and 50).

#### Linkage disequilibrium

The Plink package^65^ was used to compute *r*^2^, the correlation coefficient between pairs of loci that stands as a measure of association for linkage disequilibrium. All pairs of polymorphic sites were investigated through .map and .ped files generated with vcftools^66^, excluding SNPs with a MAF lower than 5%.

We averaged *r*^2^ based on the SNP distance (100-bp intervals) over 25-kb regions and calculated the half-length of *r*^2^, which is the distance at which linkage disequilibrium decays to half of its maximum value.

#### Loss of heterozygosity

Heterozygous isolates were investigated for LOH regions with an R script generated in-house (see ‘Data availability’). Regions over 50 kb with less than 10 heterozygous sites per 50 kb were considered to be under LOH (Supplementary Table 7).

#### Saccharomyces rooted tree

To construct the tree, we used 22 *S. cerevisiae* isolates representative of the species genetic diversity that were sequenced with Oxford Nanopore technology^67^. We annotated these 22 assemblies with the pipeline described above. The annotated protein-coding genes were pooled together with the *S. cerevisiae* reference genome (SGD R64-1-1) and another 18 yeast strains for orthology identification. These 18 other yeast strains included 7 *S. cerevisiae* strains, 5 *S. paradoxus* strains and 6 out-group strains from other *Saccharomyces* yeast species as previously described^24^. The orthology identification was carried out using Proteinortho (v.5.15)^68^ and synteny information was considered (the PoFF feature of Proteinortho). This leads to the delineation of 2,018 1-to-1 orthologous groups across all the 41 sampled genomes. For each orthologous group, the protein sequences across the 41 strains were aligned with MUSCLE (v.3.8.1551)^69^, and the resulting protein alignment was further used to guide the corresponding CDS alignment using PAL2NAL (v.14)^70^. A concatenated multi-gene matrix was built for the CDS alignment of these 2,018 orthologous groups, which was further partitioned based on codon positions (for example, 1st, 2nd and 3rd codon positions). We used RAxML (v.8.2.6) to build the maximum likelihood tree based on the GTRGAMMA model with 100 rapid bootstraps. As an alternative, we also performed phylogenetic analysis using the consensus tree approach, in which we built individual gene trees for each of the 2,018 orthologous groups using the same method described for the concatenated tree. These individual gene trees were further summarized by ASTRAL (v.4.7.12)^71^ to produce the ‘species tree’. Both the concatenated tree and the consensus tree were visualized in FigTree (v.1.4.2) (http://tree.bio.ed.ac.uk/software/figtree/).

#### Phenotyping

Quantitative high-throughput phenotyping was performed using end-point colony growth on solid medium. Strains were pregrown in flat-bottom 96-well microplates containing liquid YPD medium. The replicating ROTOR HDA benchtop robot (Singer Instruments) was used to mix and pin strains onto a solid YPD matrix plate at a density of 384 wells. The matrix plates were incubated overnight at 30 °C to allow sufficient growth and replicated on 36 medium conditions, including YPD 30 °C as the pinning and growth control condition (Supplementary Table 2). Each isolate was present in quadruplicates on the corresponding matrix (interplate replicates) and at two different positions (intraplate replicates). The plates were incubated at 30 °C for 40 h and were scanned at a resolution of 600 dpi and 16-bit grayscale. Quantification of the colony size from plate images was performed using the software package Gitter^72^. Each value was normalized using the growth ratio between stress media and standard YPD medium at 30 °C (see ‘Data availability’). Pairwise Pearson’s correlations of fitness trait values between replicates were calculated for each condition.

#### Genome-wide association studies

Mixed-model association analysis was performed using FaST-LMM v.2.07^19^. We used the normalized phenotypes by replacing the observed value with the corresponding quantile from a standard normal distribution, as FaST-LMM expects normally distributed phenotypes. In this step, we used the markers showing a MAF > 5%. We also filtered missing genotypes as ‘fs’: an arbitrary threshold has been set to exclude all variants present in less than 1,000 individuals for the total matrix.

The command used for association was the following: ‘fastlmmc -bfile $snp -bfileSim $snp -pheno $pheno -out $assoc_file –verboseOutput’.

The mixed model adds a polygenic term to the standard linear regression designed to circumvent the effects of relatedness and population stratification. To quantify the extent of the bulk inflation and the excess false positive rate, we computed the genomic inflation factor, *λ*, for each condition (Supplementary Fig. 34). This factor is defined as the ratio between the median of the empirically observed distribution of the test statistic on the expected median. For example, the *λ* for a standard allelic test for association is based on the median (0.456) of the 1-degree-of-freedom *χ*^2^ distribution. Under a null model of no association and unlinked variants, the expectation is for the *λ* to be 1. A *λ* greater than 1 indicates inflated *P* values of association, possibly owing to a confounding factor that has not been accounted for.

We estimated a trait-specific *P* value threshold for each condition by permuting phenotypic values between individuals 100 times. The significance threshold was the 5% quantile (the 5th lowest *P* value from the permutations). Using this method, variants passing this threshold have a 5% family-wise error rate (Supplementary Fig. 33).

The estimations of genome-wide heritabilities were completed by dividing the genetic variance of the null model by the total variance of the null model (genetic variance and residual variance), computed using FaST-LMM (Supplementary Fig. 6). The values reported here are based on the quantile-normalized phenotypes. To compute the variance explained by our significantly associated markers, we included them in the covariance matrix with the ‘-bfileSim’ option and performed the same calculation again (Fig. 6).

### Reporting Summary

Further information on experimental design is available in the Nature Research Reporting Summary linked to this paper.

### Code availability

The 1002 Yeast Genome website (http://1002genomes.u-strasbg.fr/files/) provides access to ‘-scripts.tar.gz’, which contains the Perl and R scripts used to (1) extract all the non-reference material from a large set of assemblies; (2) collapse ORFs that are more similar than a specific threshold; and (3) detect regions of LOH.

### Data availability

All the strains are available on request except for 11 isolates, which cannot be distributed (see Supplementary Table 1).

The Illumina reads are available in the Sequence Read Archive under accession number ERP014555.

The 1002 Yeast Genome website (http://1002genomes.u-strasbg.fr/files/) provides access to:

-1011Matrix.gvcf.gz: all SNPs and indels called at the population level (.gvcf format).

-1011GWASMatrix.tar.gz: the matrix used for GWAS, which contains all biallelic positions known for 1,000 isolates or more with MAF > 5% as well as CNVs (encoded 0/1/2 for absence/0.5–1 copy/multiple copies) (.bed,.bim and.fam formats).

-1011DistanceMatrixBasedOnSNPs.tab.gz: for each pair of strains, the value is the percentage, based on SNPs, of non-identical bases. Heterozygous differences were half-weighted compared to the homozygous differences. 

-1011DistanceMatrixBasedOnORFs.tab.gz: for each couple of strains, the value is the number of ORFs that are present in only one out of the two isolates. 

-1011Assemblies.tar.gz: de novo assemblies of the 1,011 isolates (.fasta format). 

-allReferenceGenesWithSNPsAndIndelsInferred.tar.gz: sequences of the genes found in the reference genome in which SNPs and indels have been automatically inferred for each isolate. 

-allORFs_pangenome.fasta.gz: sequences of the 7,796 pangenomic ORFs (.fasta format). 

-genesMatrix_PresenceAbsence.tab.gz: pattern of presence and/or absence of pangenomic ORFs for each isolate, in which the presence of an ORF is marked as 1 and its absence is marked as 0. 

-genesMatrix_CopyNumber.tab.gz: estimated copy number for each pangenomic ORF, per isolate. Values are given for the haploid genome, so that non-integer values can be found (different copy number on homologous chromosomes). 

-genesMatrix_Frameshift.tab.gz: for each isolate, the presence or absence (indicated by 1 or 0, respectively) of homozygous frameshift is reported in each gene, based on the number of bases affected by indels. 

-phenoMatrix_35ConditionsNormalizedByYPD.tab.gz: growth ratio between 35 stress conditions and standard YPD medium at 30 °C, for 971 isolates.

All other data are available from the corresponding authors upon reasonable request.

## Online content

Any Methods, including any statements of data availability and Nature Research reporting summaries, along with any additional references and Source Data files, are available in the online version of the paper at 10.1038/s41586-018-0030-5.

## Supplementary Information

Supplementary Information This file contains Supplementary Figures 1-34, the Supplementary Figure Legends, Supplementary Notes 1-4 and Supplementary References

Reporting Summary

Supplementary Tables This file contains Supplementary Tables 1-21
