# Supplementary material for: Genome evolution across 1,011 Saccharomyces cerevisiae isolates
Source: Nature. 2018 Apr 11;556(7701):339–44. doi: 10.1038/s41586-018-0030-5 (PMC6784862; doi:10.1038/s41586-018-0030-5)
Supplement: Supplementary file 1 — This file contains Supplementary Figures 1-34, the Supplementary Figure Legends, Supplementary Notes 1-4 and Supplementary References [file 41586_2018_30_MOESM1_ESM.pdf]

nature SUPPLEMENTARY INFORMATION
https://doi.org/10.1038/s41586-018-0030-5

In the format provided by the authors and unedited.

Genome evolution across 1,011 *Saccharomyces cerevisiae* isolates

Jackson Peter[1,6], Matteo De Chiara[2,6], Anne Friedrich[1], Jia-Xing Yue[2], David Pflieger[1], Anders Bergström[2], Anastasie Sigwalt[1], Benjamin Barre[2], Kelle Freel[1], Agnès Llored[2], Corinne Cruaud[3], Karine Labadie[3], Jean-Marc Aury[3], Benjamin Istace[3], Kevin Lebrigand[4], Pascal Barbry[4], Stefan Engelen[3], Arnaud Lemainque[3], Patrick Wincker[3,5,7], Gianni Liti[2,7]* & Joseph Schacherer[1,7]*

[1]Université de Strasbourg, CNRS, GMGM UMR 7156, Strasbourg, France. [2]Université Côte d'Azur, CNRS, INSERM, IRCAN, Nice, France. [3]Commissariat à l'Energie Atomique (CEA), Genoscope, Institut de Biologie François-Jacob, Evry, France. [4]Université Côte d'Azur, CNRS, IPMC, Sophia Antipolis, Valbonne, France. [5]CNRS UMR 8030, Université d'Evry Val d'Essonne, Evry, France. [6]These authors contributed equally: Jackson Peter, Matteo De Chiara. [7]These authors jointly supervised this work: Patrick Wincker, Gianni Liti, Joseph Schacherer. *e-mail: gianni.liti@unice.fr; schacherer@unistra.fr

© 2018 Macmillan Publishers Limited, part of Springer Nature. All rights reserved.

Supplementary figures and notes

Genome evolution across 1,011 *Saccharomyces cerevisiae* isolates

Jackson Peter[1,†], Matteo De Chiara[2,†], Anne Friedrich[1], Jia-Xing Yue[2], David Pflieger[1], Anders Bergström[2], Anastasie Sigwalt[1], Benjamin Barre[2], Kelle Freel[1], Agnès Llored[2], Corinne Cruaud[3], Karine Labadie[3], Jean-Marc Aury[3], Benjamin Istace[3], Kevin Lebrigand[4], Pascal Barbry[4], Stefan Engelen[3], Arnaud Lemainque[3], Patrick Wincker[3,5], Gianni Liti[2,]* and Joseph Schacherer[1,]*

[1] Université de Strasbourg, CNRS, GMGM UMR 7156, F-67000 Strasbourg, France
[2] Université Côte d'Azur, CNRS, INSERM, IRCAN, Nice, France
[3] Commissariat à l'Energie Atomique (CEA), Genoscope, Institut de biologie François-Jacob, BP5706, 91057 Evry, France
[4] Université Côte d'Azur, CNRS, IPMC, Sophia-Antipolis, Valbonne, France
[5] CNRS UMR 8030, Université d'Evry Val d'Essonne, Evry, France

Figure S1

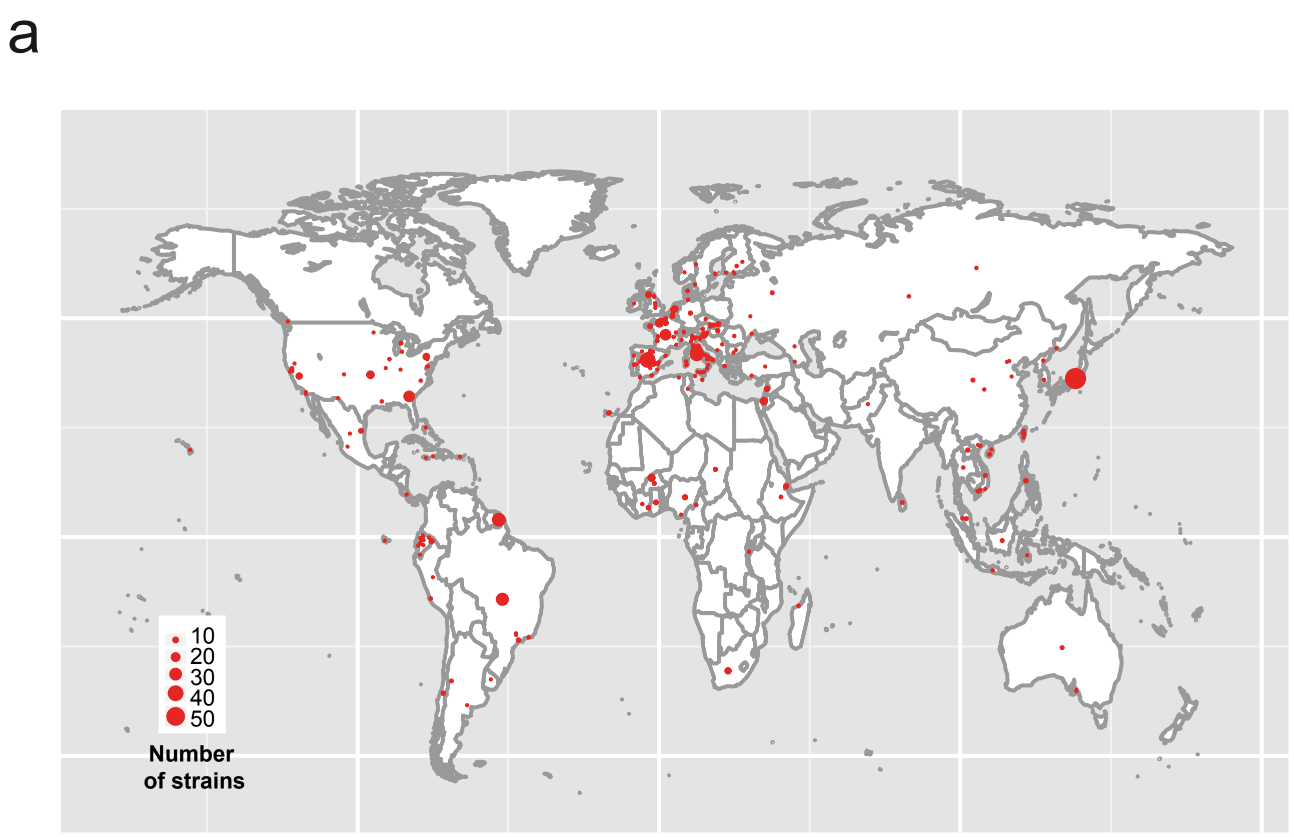
a
10
20
30
40
50
Number
of strains

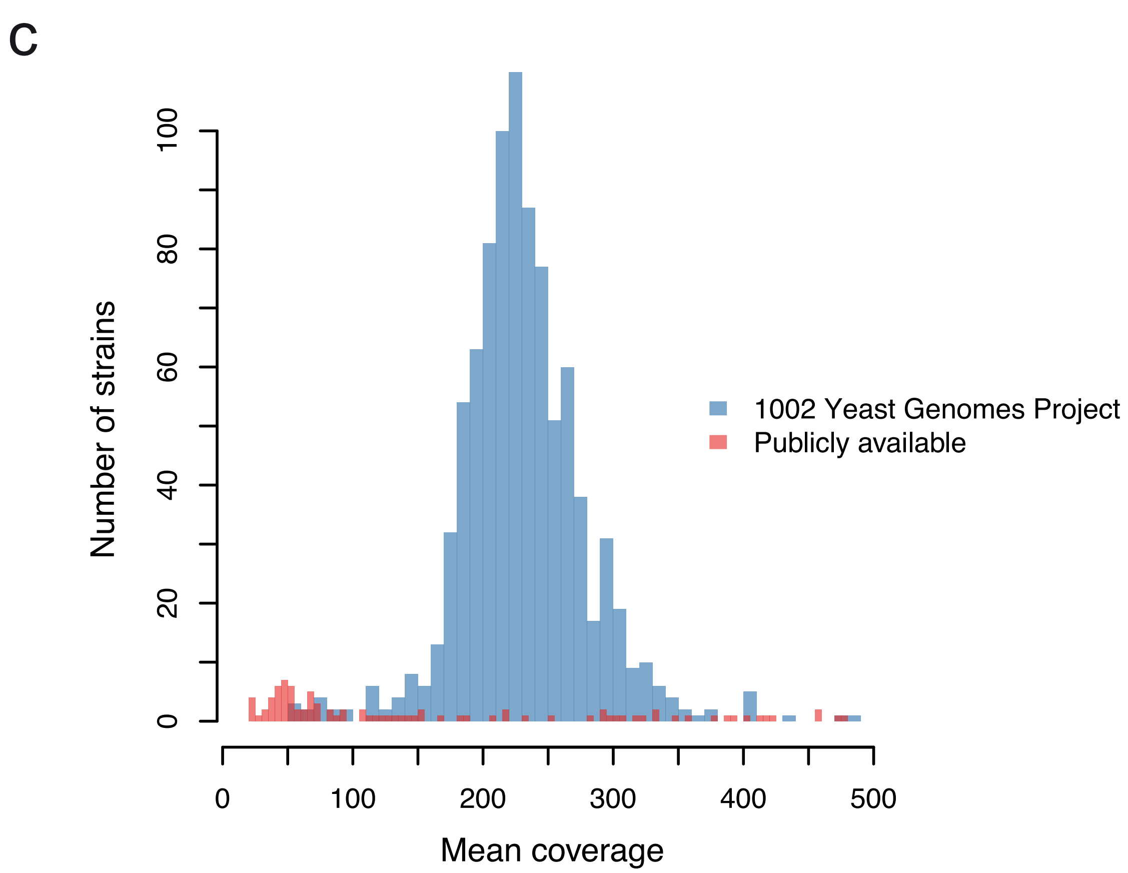
c
Number of strains
0
20
40
60
80
100
0
100
200
300
400
500
Mean coverage
1002 Yeast Genomes Project
Publicly available

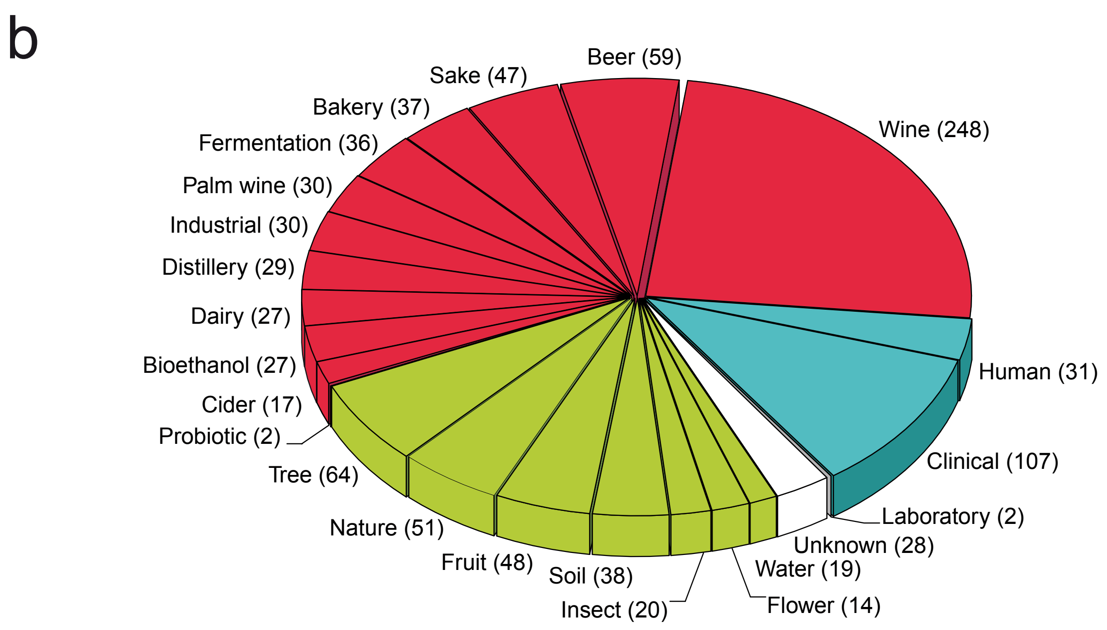
b
Beer (59)
Sake (47)
Bakery (37)
Fermentation (36)
Palm wine (30)
Industrial (30)
Distillery (29)
Dairy (27)
Bioethanol (27)
Cider (17)
Probiotic (2)
Tree (64)
Nature (51)
Fruit (48)
Soil (38)
Insect (20)
Flower (14)
Water (19)
Unknown (28)
Laboratory (2)
Clinical (107)
Human (31)
Wine (248)

Figure S2

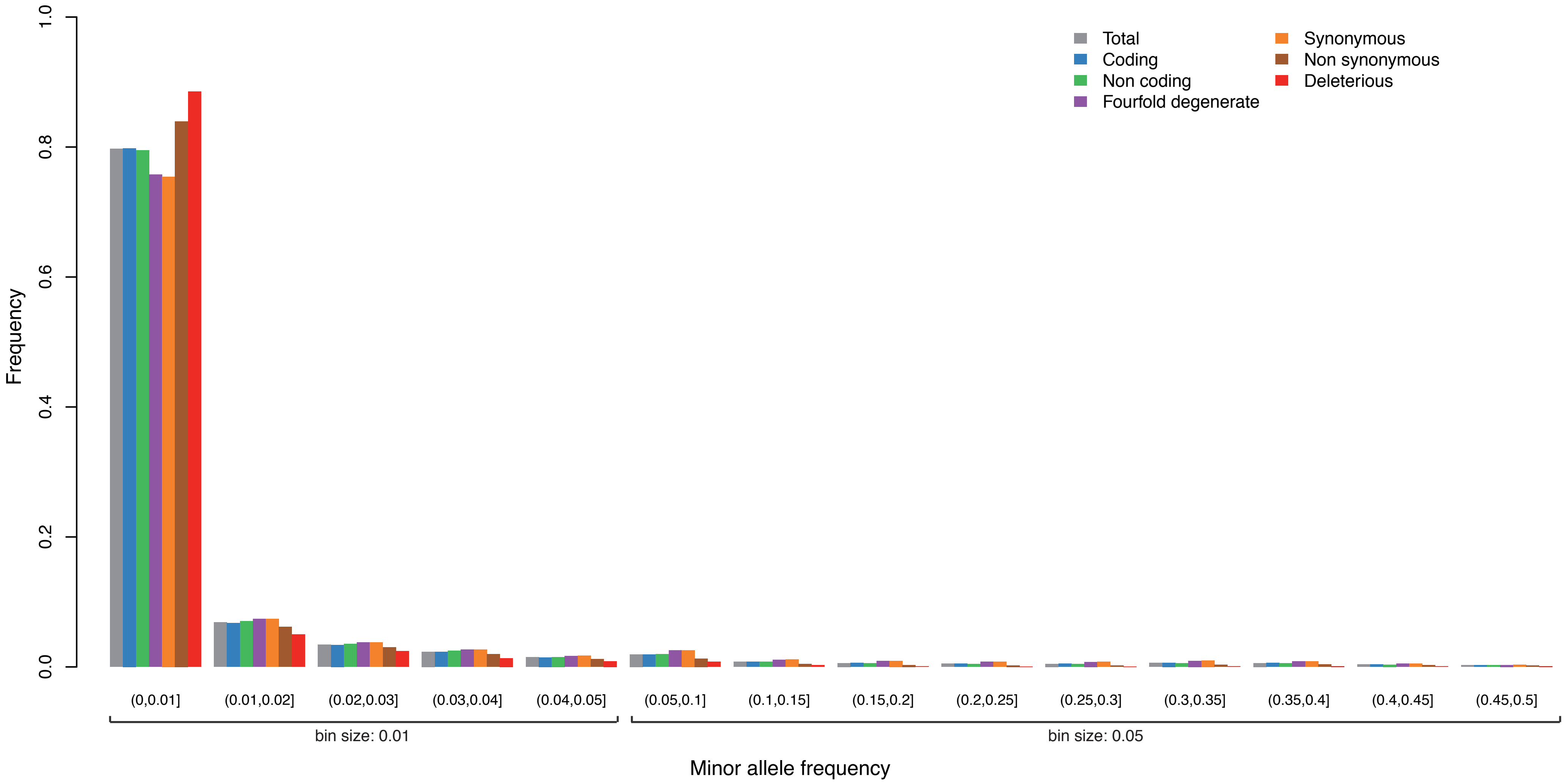

Total
Coding
Non coding
Fourfold degenerate
Synonymous
Non synonymous
Deleterious
Frequency
0.0
0.2
0.4
0.6
0.8
1.0
(0,0.01]
(0.01,0.02]
(0.02,0.03]
(0.03,0.04]
(0.04,0.05]
(0.05,0.1]
(0.1,0.15]
(0.15,0.2]
(0.2,0.25]
(0.25,0.3]
(0.3,0.35]
(0.35,0.4]
(0.4,0.45]
(0.45,0.5]
bin size: 0.01
bin size: 0.05
Minor allele frequency

Figure S3

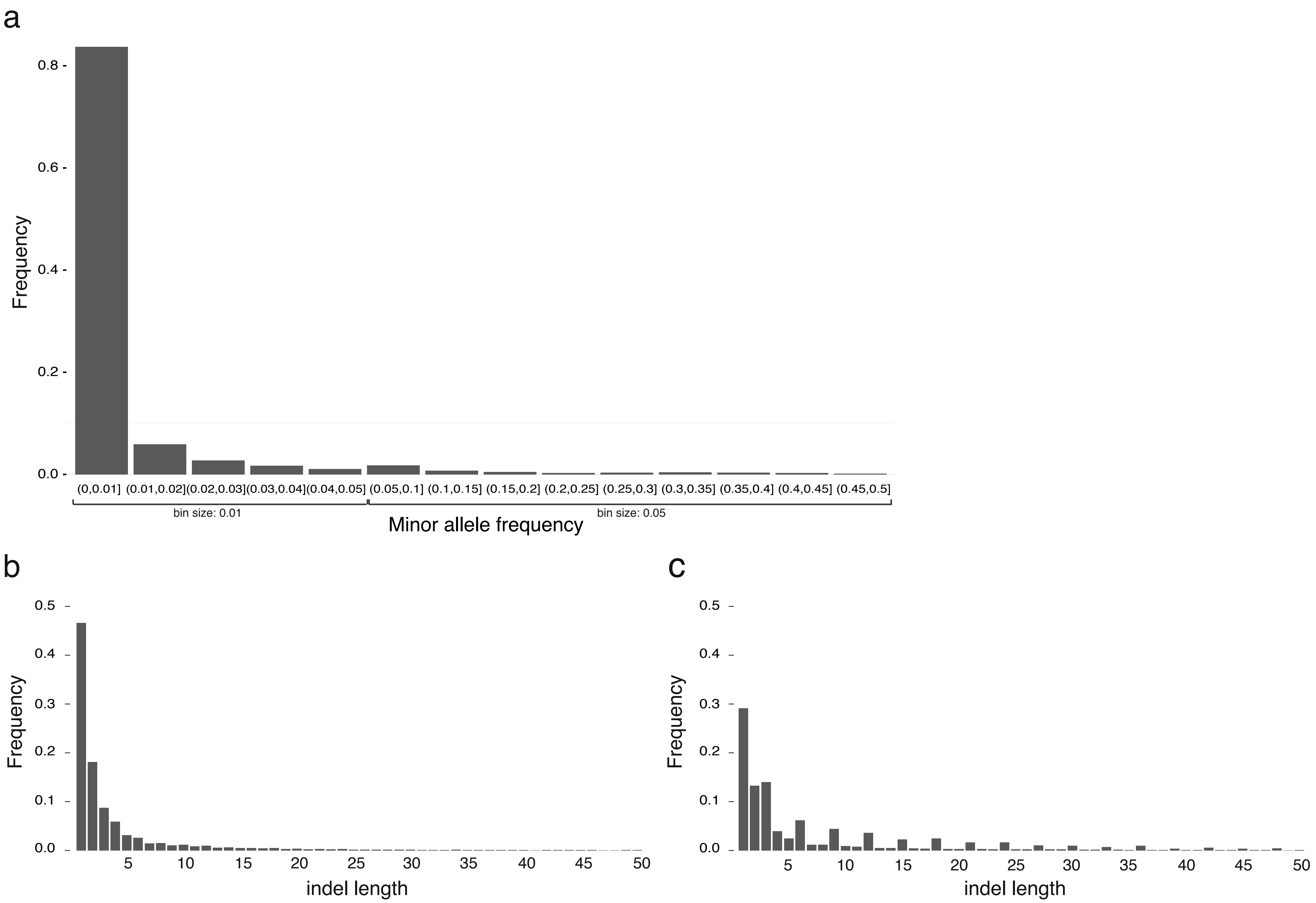
a
0.8
0.6
0.4
0.2
0.0
Frequency
(0,0.01] (0.01,0.02] (0.02,0.03] (0.03,0.04] (0.04,0.05] (0.05,0.1] (0.1,0.15] (0.15,0.2] (0.2,0.25] (0.25,0.3] (0.3,0.35] (0.35,0.4] (0.4,0.45] (0.45,0.5]
bin size: 0.01
bin size: 0.05
Minor allele frequency
b
c
0.5
0.4
0.3
0.2
0.1
0.0
Frequency
5 10 15 20 25 30 35 40 45 50
indel length

Figure S4

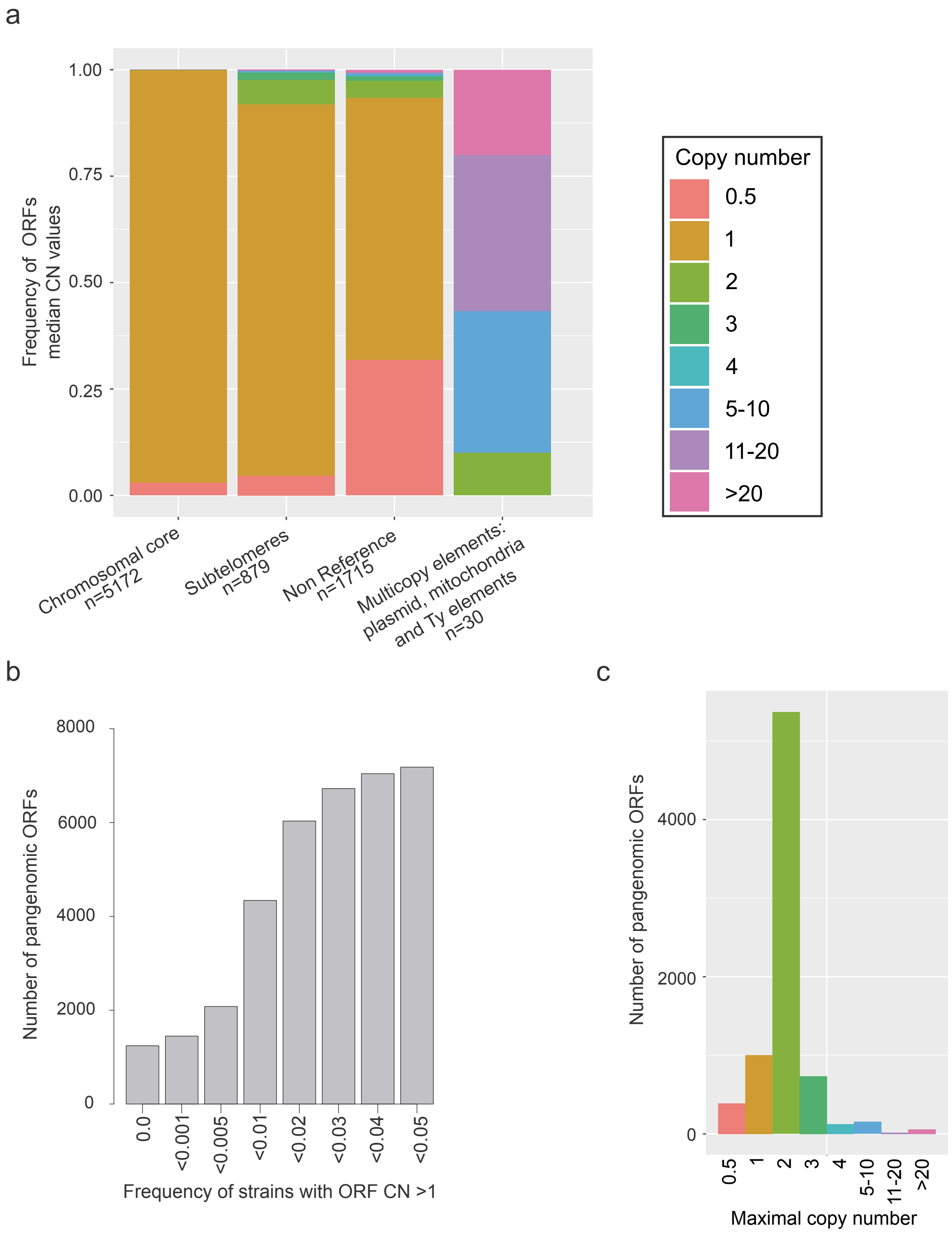
a
Frequency of ORFs median CN values
1.00
0.75
0.50
0.25
0.00
Chromosomal core n=5172
Subtelomeres n=879
Non Reference n=1715
Multicopy elements: plasmid, mitochondria and Ty elements n=30
Copy number
0.5
1
2
3
4
5-10
11-20
>20
b
Number of pangenomic ORFs
8000
6000
4000
2000
0
0.0
<0.001
<0.005
<0.01
<0.02
<0.03
<0.04
<0.05
Frequency of strains with ORF CN >1
c
Number of pangenomic ORFs
4000
2000
0
0.5
1
2
3
4
5-10
11-20
>20
Maximal copy number

Figure S5

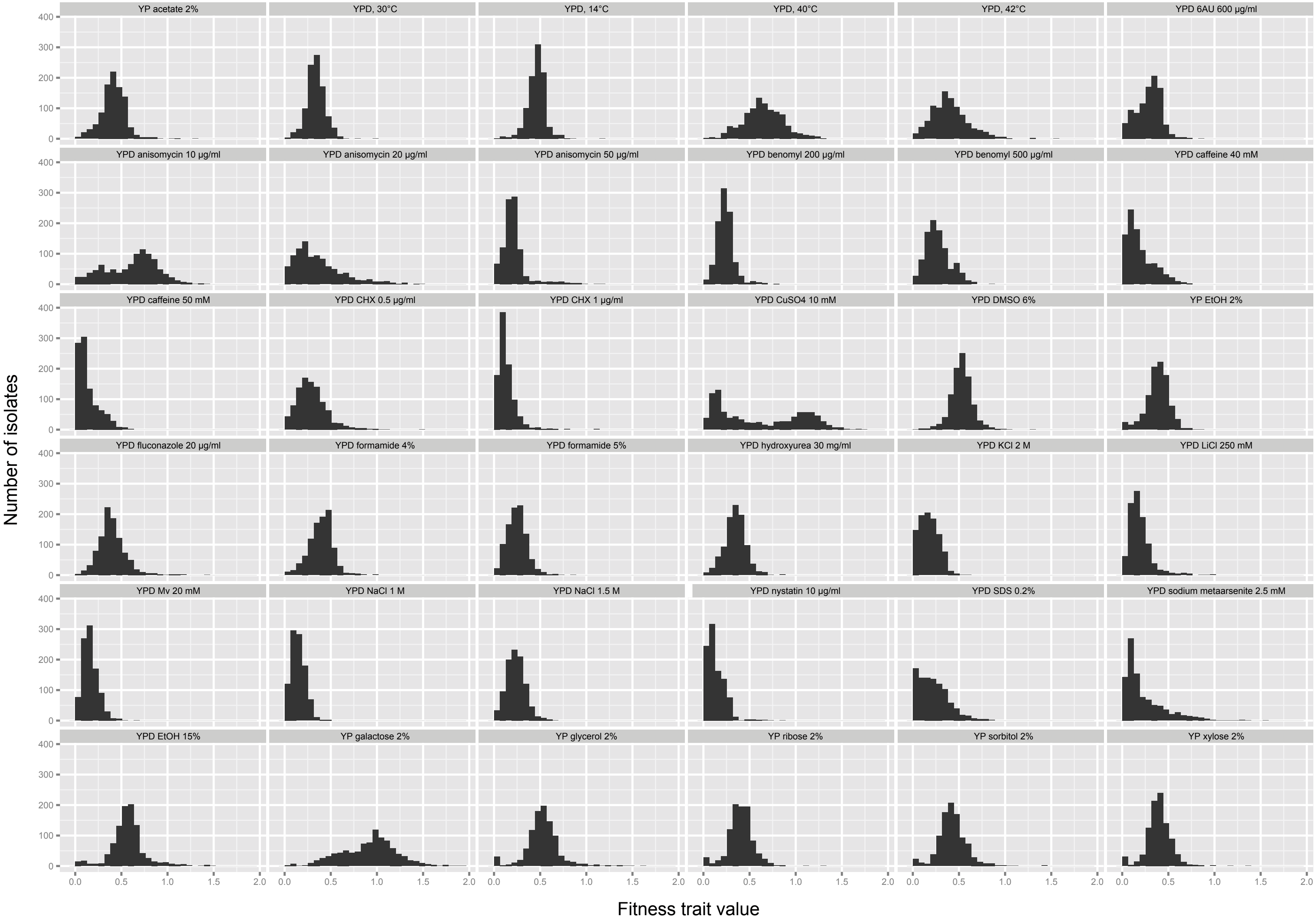

YP acetate 2%
YPD, 30°C
YPD, 14°C
YPD, 40°C
YPD, 42°C
YPD 6AU 600 µg/ml
YPD anisomycin 10 µg/ml
YPD anisomycin 20 µg/ml
YPD anisomycin 50 µg/ml
YPD benomyl 200 µg/ml
YPD benomyl 500 µg/ml
YPD caffeine 40 mM
YPD caffeine 50 mM
YPD CHX 0.5 µg/ml
YPD CHX 1 µg/ml
YPD CuSO4 10 mM
YPD DMSO 6%
YP EtOH 2%
YPD fluconazole 20 µg/ml
YPD formamide 4%
YPD formamide 5%
YPD hydroxyurea 30 mg/ml
YPD KCl 2 M
YPD LiCl 250 mM
YPD Mv 20 mM
YPD NaCl 1 M
YPD NaCl 1.5 M
YPD nystatin 10 µg/ml
YPD SDS 0.2%
YPD sodium metaarsenite 2.5 mM
YPD EtOH 15%
YP galactose 2%
YP glycerol 2%
YP ribose 2%
YP sorbitol 2%
YP xylose 2%
Number of isolates
0
100
200
300
400
0.0
0.5
1.0
1.5
2.0
Fitness trait value

Figure S6

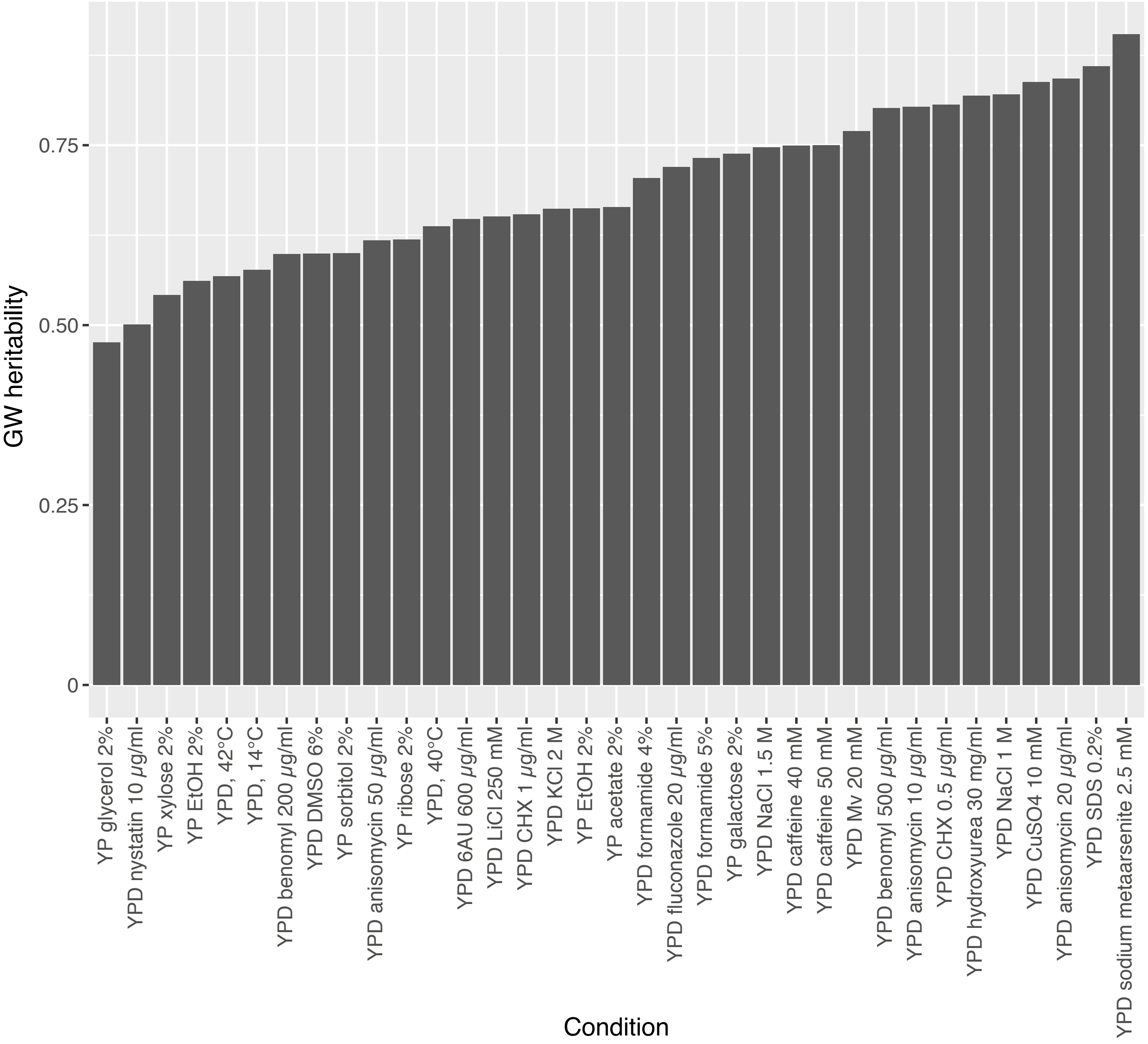

GW heritability
0.75
0.50
0.25
0
Condition
YP glycerol 2%
YPD nystatin 10 μg/ml
YP xylose 2%
YP EtOH 2%
YPD, 42°C
YPD, 14°C
YPD benomyl 200 μg/ml
YPD DMSO 6%
YP sorbitol 2%
YPD anisomycin 50 μg/ml
YP ribose 2%
YPD, 40°C
YPD 6AU 600 μg/ml
YPD LiCl 250 mM
YPD CHX 1 μg/ml
YPD KCl 2 M
YP EtOH 2%
YP acetate 2%
YPD formamide 4%
YPD fluconazole 20 μg/ml
YPD formamide 5%
YP galactose 2%
YPD NaCl 1.5 M
YPD caffeine 40 mM
YPD caffeine 50 mM
YPD Mv 20 mM
YPD benomyl 500 μg/ml
YPD anisomycin 10 μg/ml
YPD CHX 0.5 μg/ml
YPD hydroxyurea 30 mg/ml
YPD NaCl 1 M
YPD CuSO4 10 mM
YPD anisomycin 20 μg/ml
YPD SDS 0.2%
YPD sodium metaarsenite 2.5 mM

Figure S7

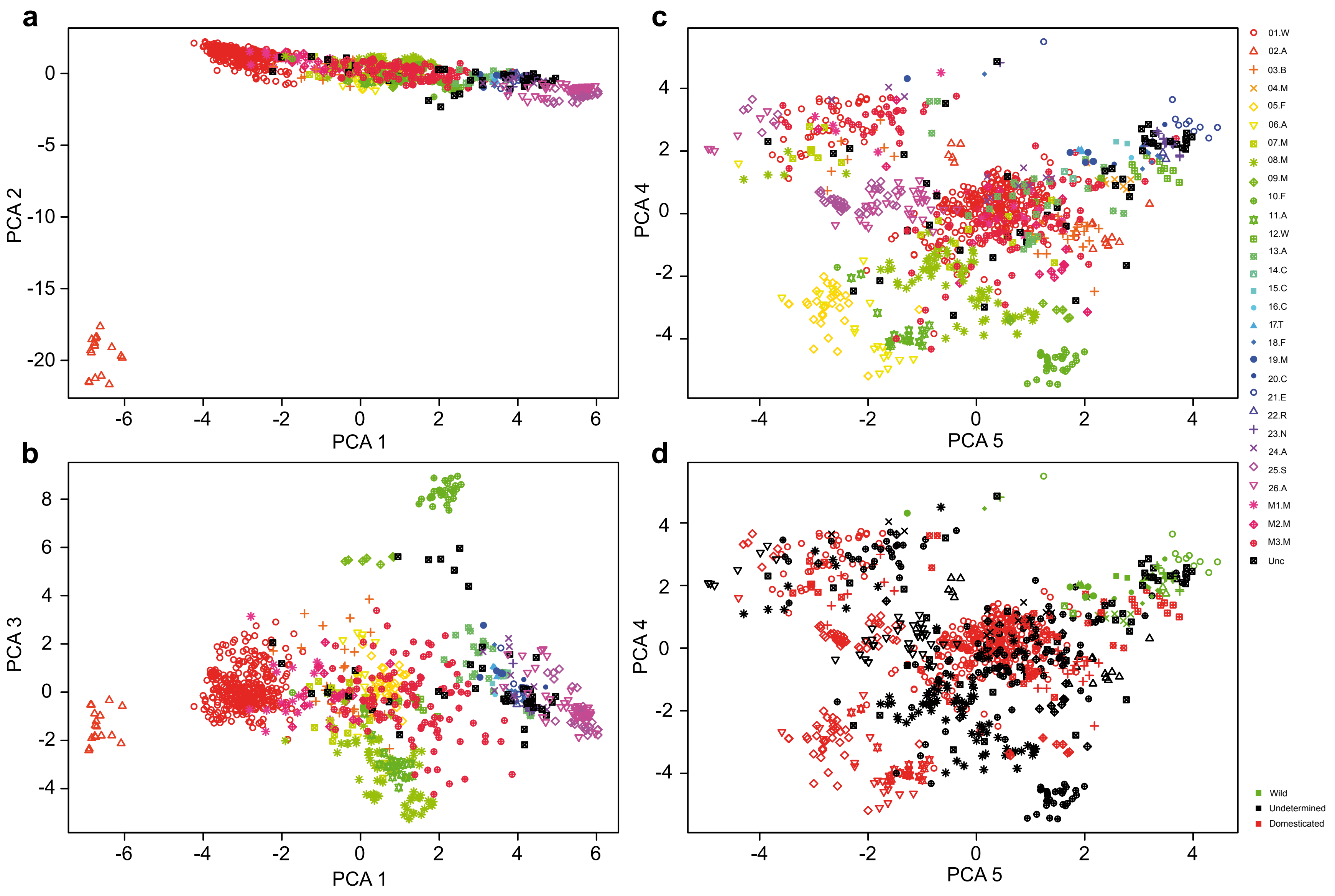

a
b
c
d
PCA 1
PCA 2
PCA 3
PCA 4
PCA 5
01.W
02.A
03.B
04.M
05.F
06.A
07.M
08.M
09.M
10.F
11.A
12.W
13.A
14.C
15.C
16.C
17.T
18.F
19.M
20.C
21.E
22.R
23.N
24.A
25.S
26.A
M1.M
M2.M
M3.M
Unc
Wild
Undetermined
Domesticated

Figure S8

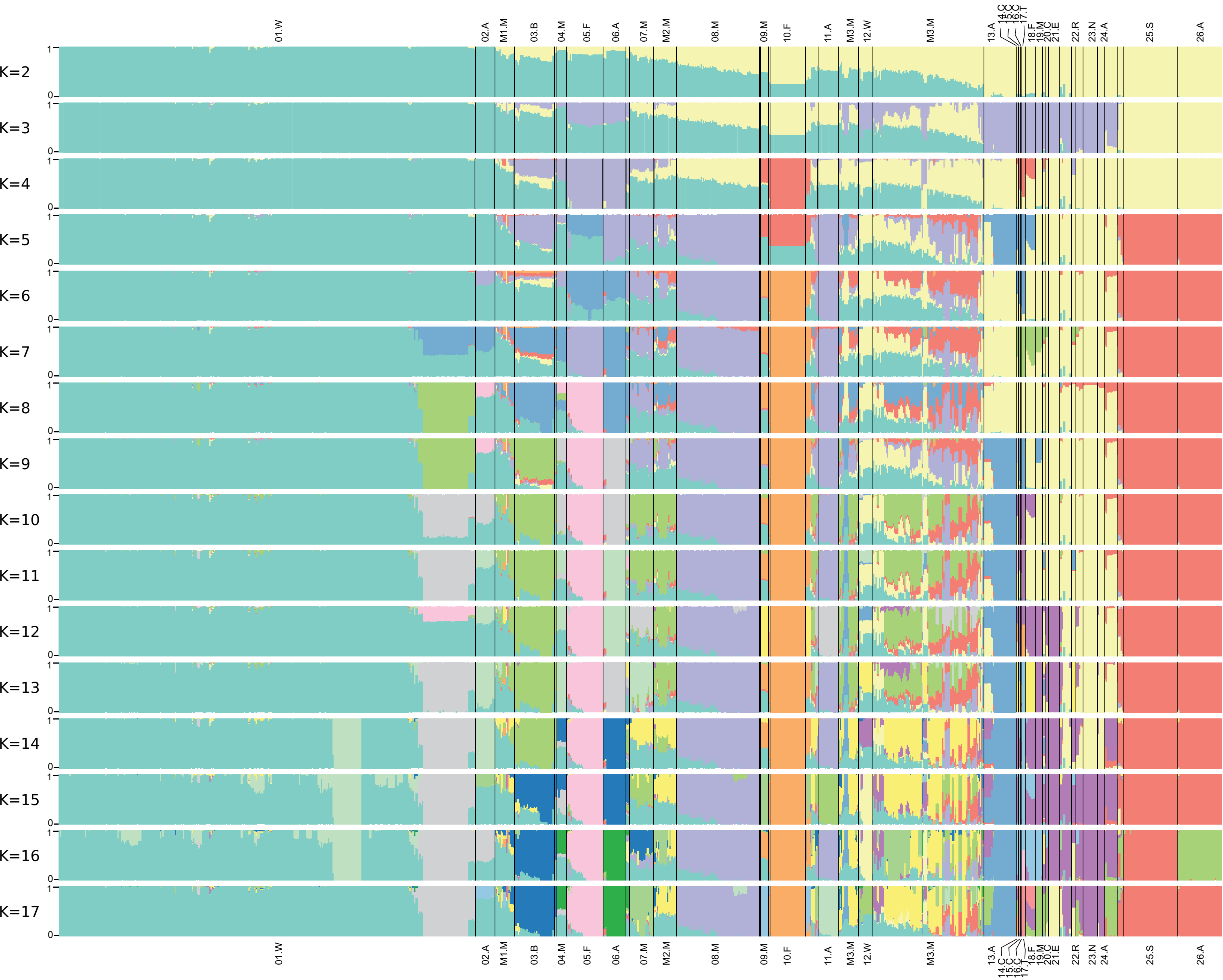
01.W
02.A
M1.M
03.B
04.M
05.F
06.A
07.M
M2.M
08.M
09.M
10.F
11.A
M3.M
12.W
M3.M
13.A
14.C
15.C
16.C
17.T
18.F
19.M
20.C
21.E
22.R
23.N
24.A
25.S
26.A
K=2
K=3
K=4
K=5
K=6
K=7
K=8
K=9
K=10
K=11
K=12
K=13
K=14
K=15
K=16
K=17
1
0

Figure S9

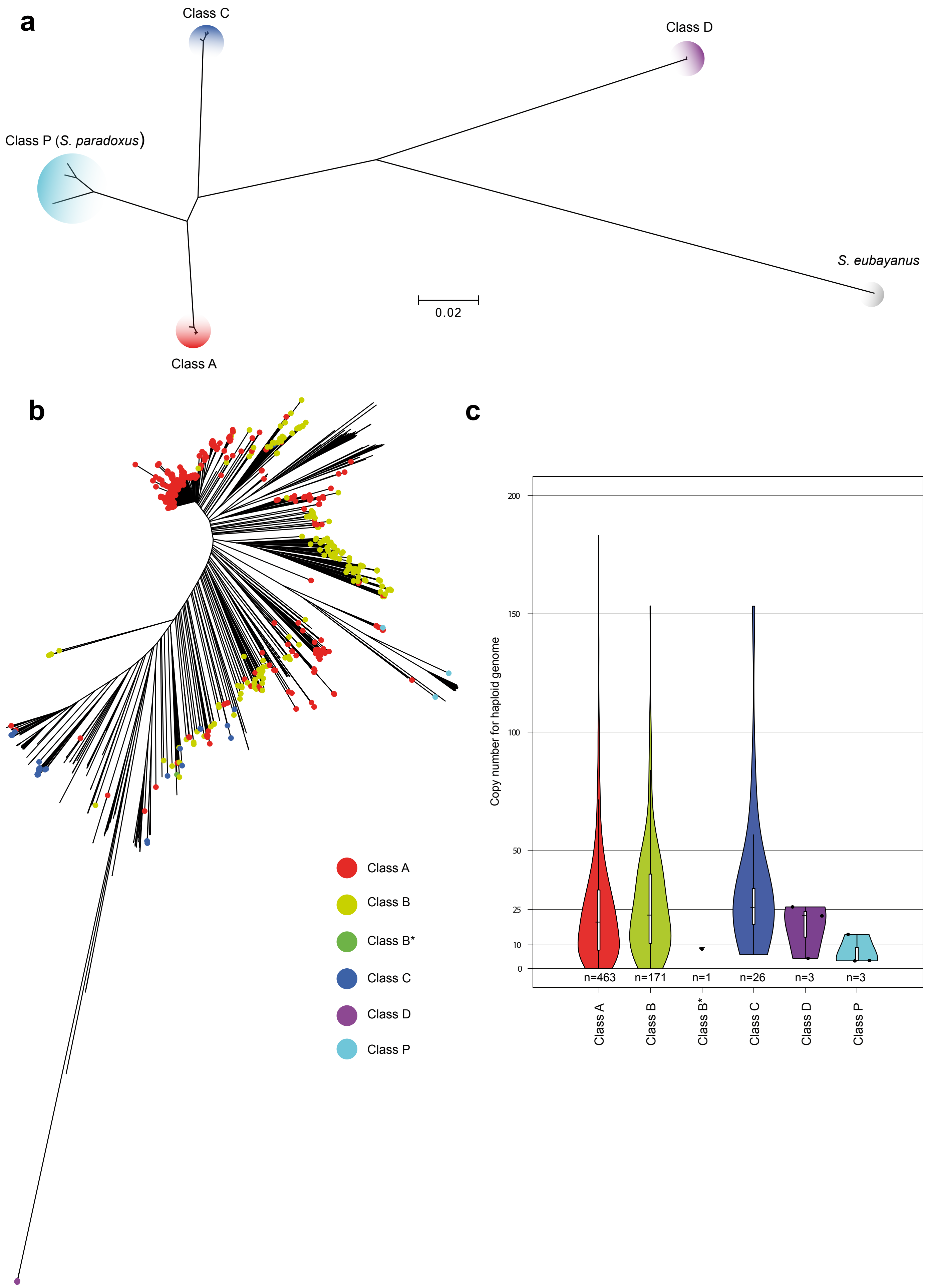
a
Class C
Class D
Class P (S. paradoxus)
S. eubayanus
0.02
Class A
b
Class A
Class B
Class B*
Class C
Class D
Class P
c
Copy number for haploid genome
200
150
100
50
25
10
0
n=463
n=171
n=1
n=26
n=3
n=3
Class A
Class B
Class B*
Class C
Class D
Class P

Figure S10

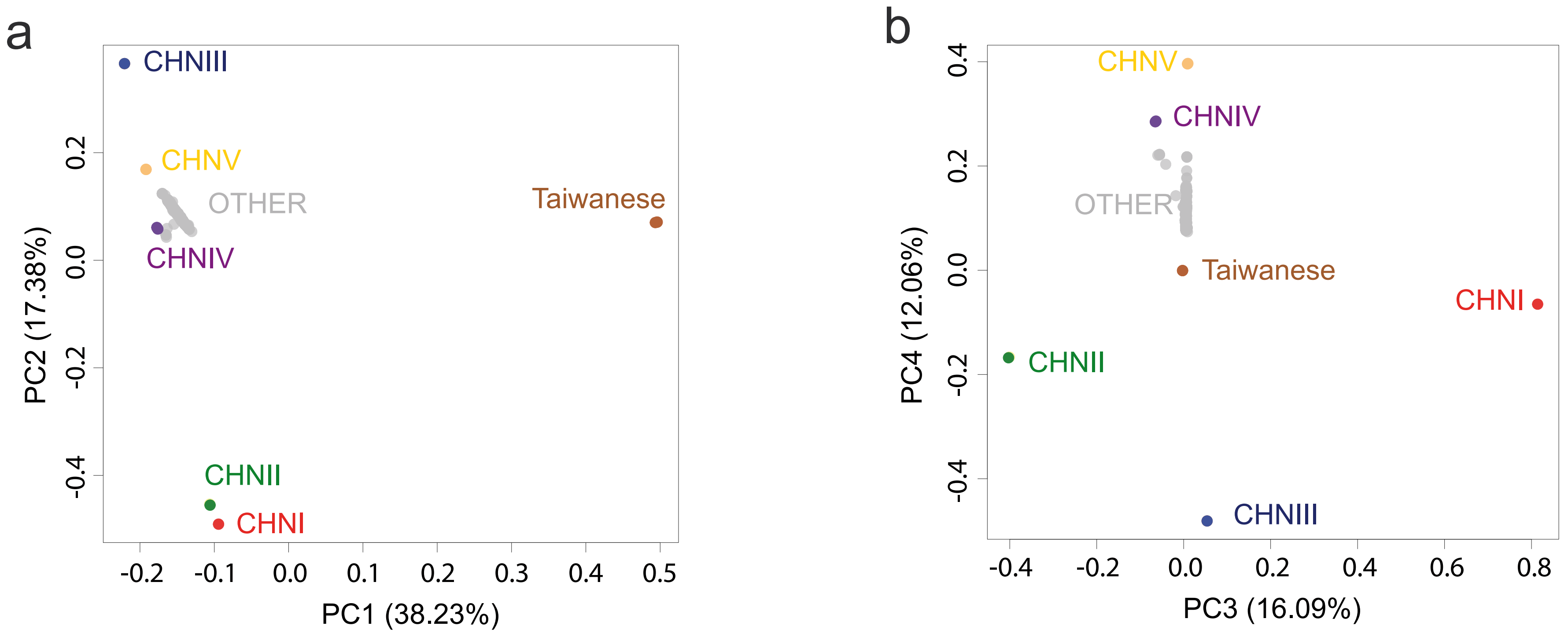

a
CHNIII
CHNV
OTHER
Taiwanese
CHNIV
CHNII
CHNI
0.2
0.0
-0.2
-0.4
PC2 (17.38%)
-0.2
-0.1
0.0
0.1
0.2
0.3
0.4
0.5
PC1 (38.23%)
b
CHNV
CHNIV
OTHER
Taiwanese
CHNI
CHNII
CHNIII
0.4
0.2
0.0
-0.2
-0.4
PC4 (12.06%)
-0.4
-0.2
0.0
0.2
0.4
0.6
0.8
PC3 (16.09%)

Figure S11

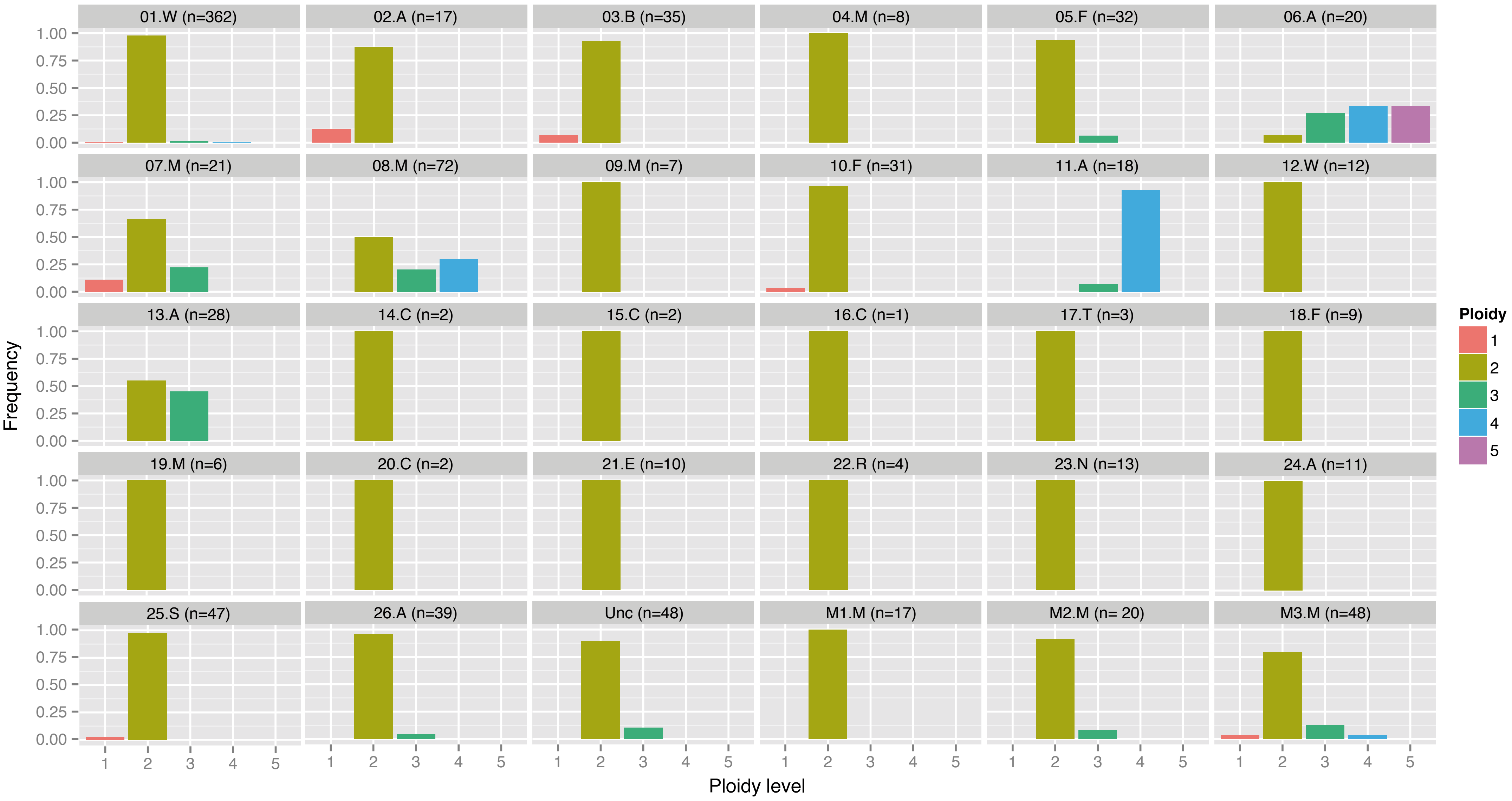
01.W (n=362)
02.A (n=17)
03.B (n=35)
04.M (n=8)
05.F (n=32)
06.A (n=20)
07.M (n=21)
08.M (n=72)
09.M (n=7)
10.F (n=31)
11.A (n=18)
12.W (n=12)
13.A (n=28)
14.C (n=2)
15.C (n=2)
16.C (n=1)
17.T (n=3)
18.F (n=9)
19.M (n=6)
20.C (n=2)
21.E (n=10)
22.R (n=4)
23.N (n=13)
24.A (n=11)
25.S (n=47)
26.A (n=39)
Unc (n=48)
M1.M (n=17)
M2.M (n= 20)
M3.M (n=48)
Frequency
Ploidy level
Ploidy
1
2
3
4
5

Figure S12

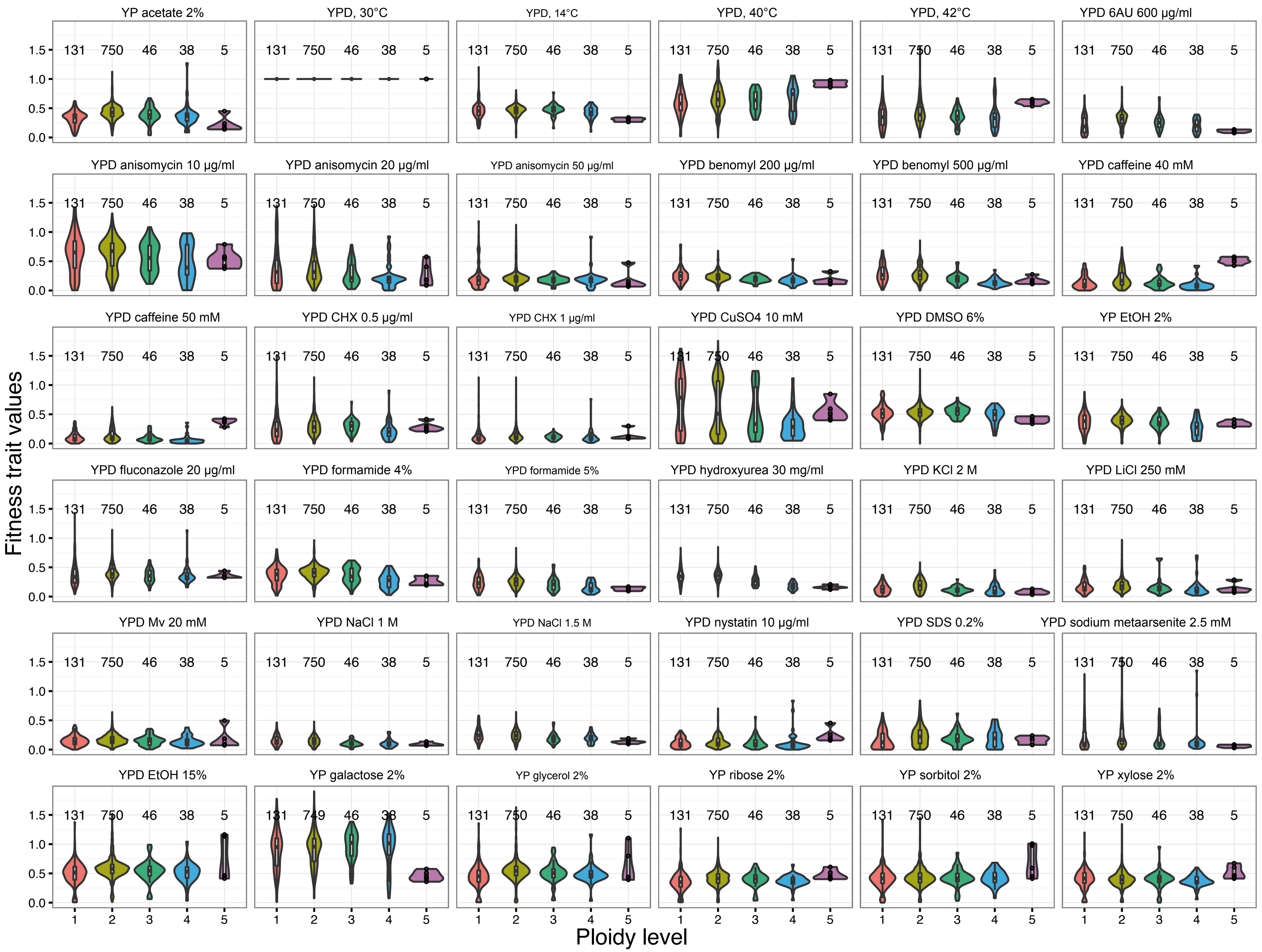

YP acetate 2%
YPD, 30°C
YPD, 14°C
YPD, 40°C
YPD, 42°C
YPD 6AU 600 µg/ml
YPD anisomycin 10 µg/ml
YPD anisomycin 20 µg/ml
YPD anisomycin 50 µg/ml
YPD benomyl 200 µg/ml
YPD benomyl 500 µg/ml
YPD caffeine 40 mM
YPD caffeine 50 mM
YPD CHX 0.5 µg/ml
YPD CHX 1 µg/ml
YPD CuSO4 10 mM
YPD DMSO 6%
YP EtOH 2%
YPD fluconazole 20 µg/ml
YPD formamide 4%
YPD formamide 5%
YPD hydroxyurea 30 mg/ml
YPD KCl 2 M
YPD LiCl 250 mM
YPD Mv 20 mM
YPD NaCl 1 M
YPD NaCl 1.5 M
YPD nystatin 10 µg/ml
YPD SDS 0.2%
YPD sodium metaarsenite 2.5 mM
YPD EtOH 15%
YP galactose 2%
YP glycerol 2%
YP ribose 2%
YP sorbitol 2%
YP xylose 2%
131 750 46 38 5
131 749 46 38 5
Fitness trait values
Ploidy level

Figure S13

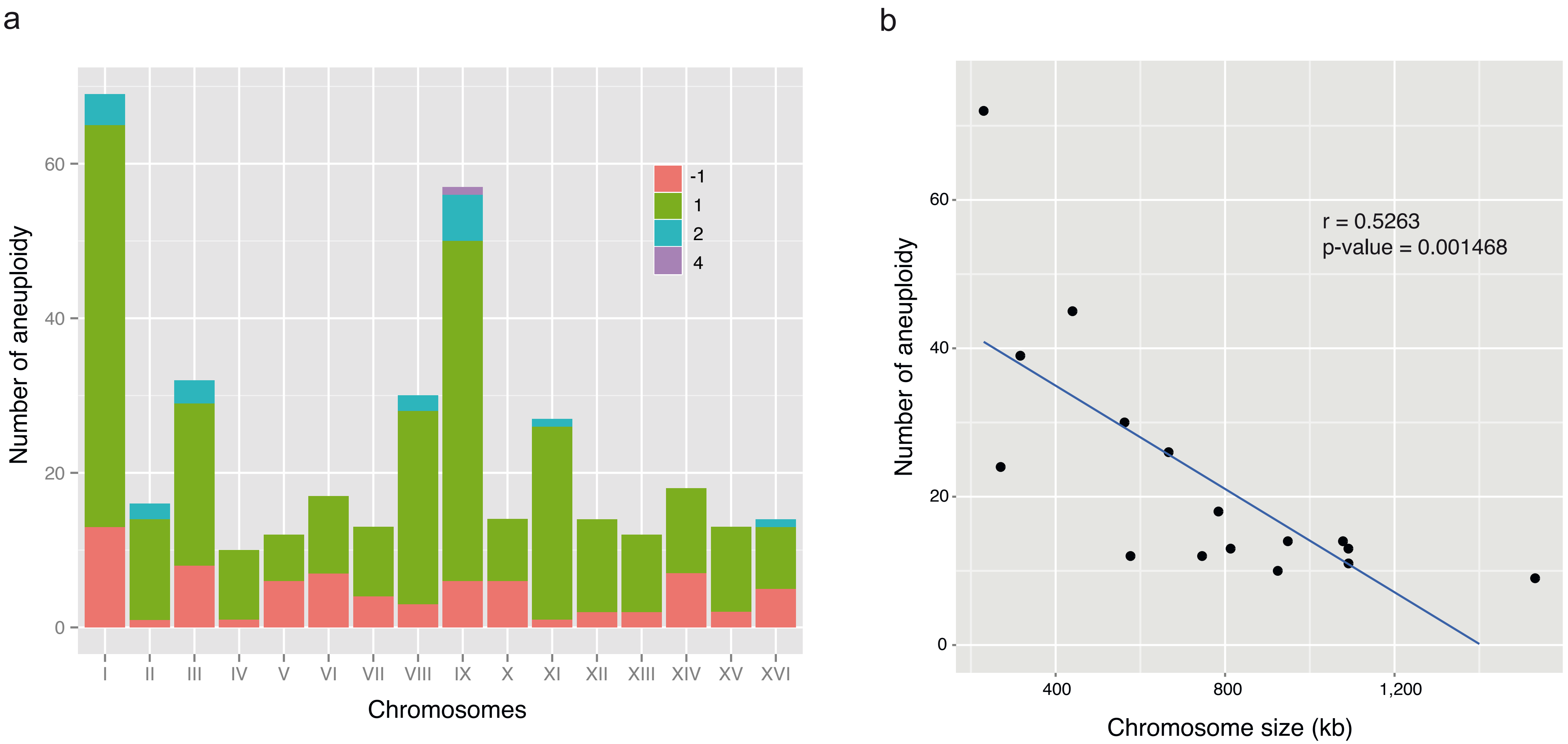
a
b
Number of aneuploidy
Chromosomes
I
II
III
IV
V
VI
VII
VIII
IX
X
XI
XII
XIII
XIV
XV
XVI
0
20
40
60
-1
1
2
4
Number of aneuploidy
Chromosome size (kb)
400
800
1,200
r = 0.5263
p-value = 0.001468

Figure S14

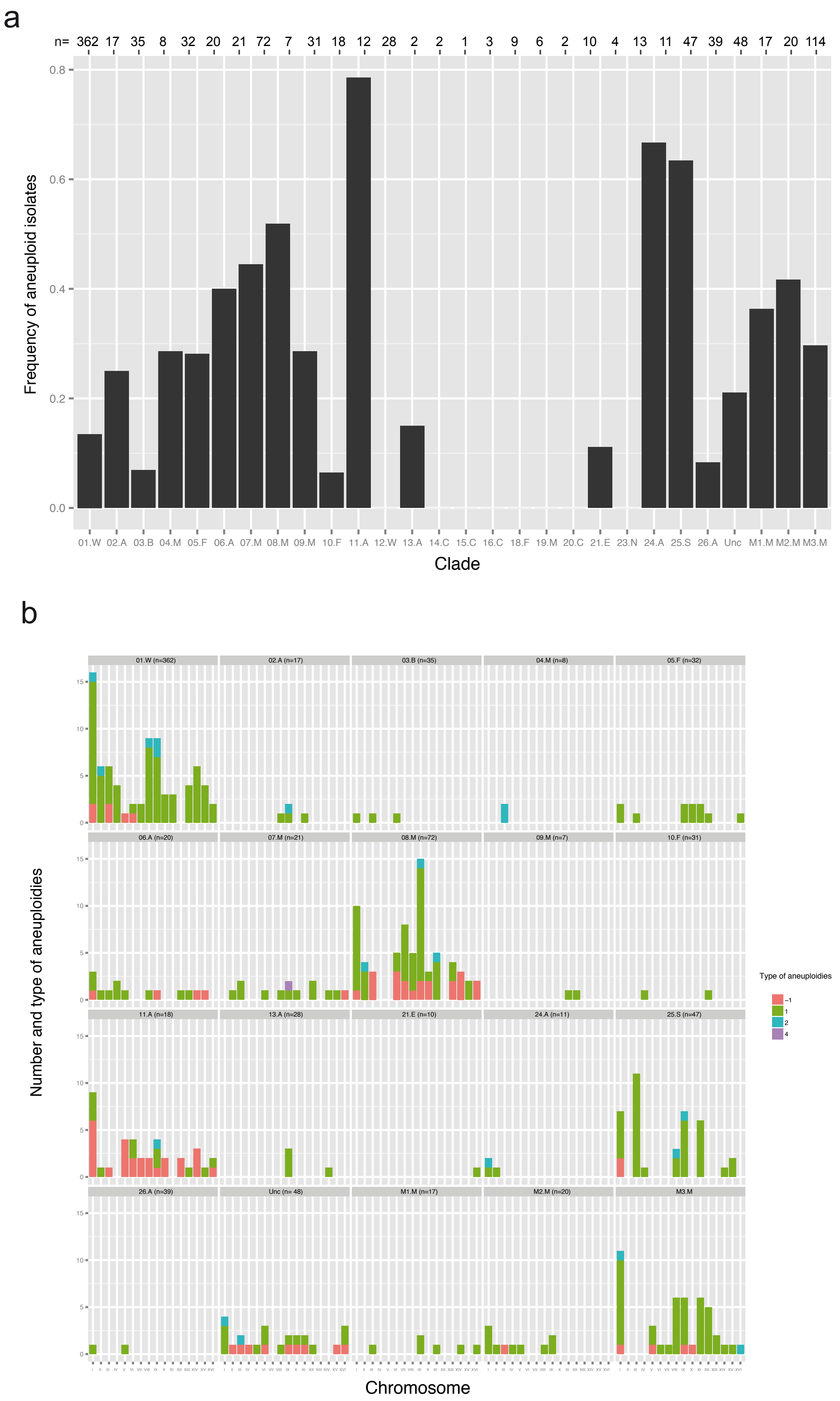
a
n= 362 17 35 8 32 20 21 72 7 31 18 12 28 2 2 1 3 9 6 2 10 4 13 11 47 39 48 17 20 114
Frequency of aneuploid isolates
0.0
0.2
0.4
0.6
0.8
01.W 02.A 03.B 04.M 05.F 06.A 07.M 08.M 09.M 10.F 11.A 12.W 13.A 14.C 15.C 16.C 18.F 19.M 20.C 21.E 23.N 24.A 25.S 26.A Unc M1.M M2.M M3.M
Clade
b
01.W (n=362)
02.A (n=17)
03.B (n=35)
04.M (n=8)
05.F (n=32)
06.A (n=20)
07.M (n=21)
08.M (n=72)
09.M (n=7)
10.F (n=31)
11.A (n=18)
13.A (n=28)
21.E (n=10)
24.A (n=11)
25.S (n=47)
26.A (n=39)
Unc (n= 48)
M1.M (n=17)
M2.M (n=20)
M3.M
Number and type of aneuploidies
Chromosome
Type of aneuploidies
−1
1
2
4

Figure S15

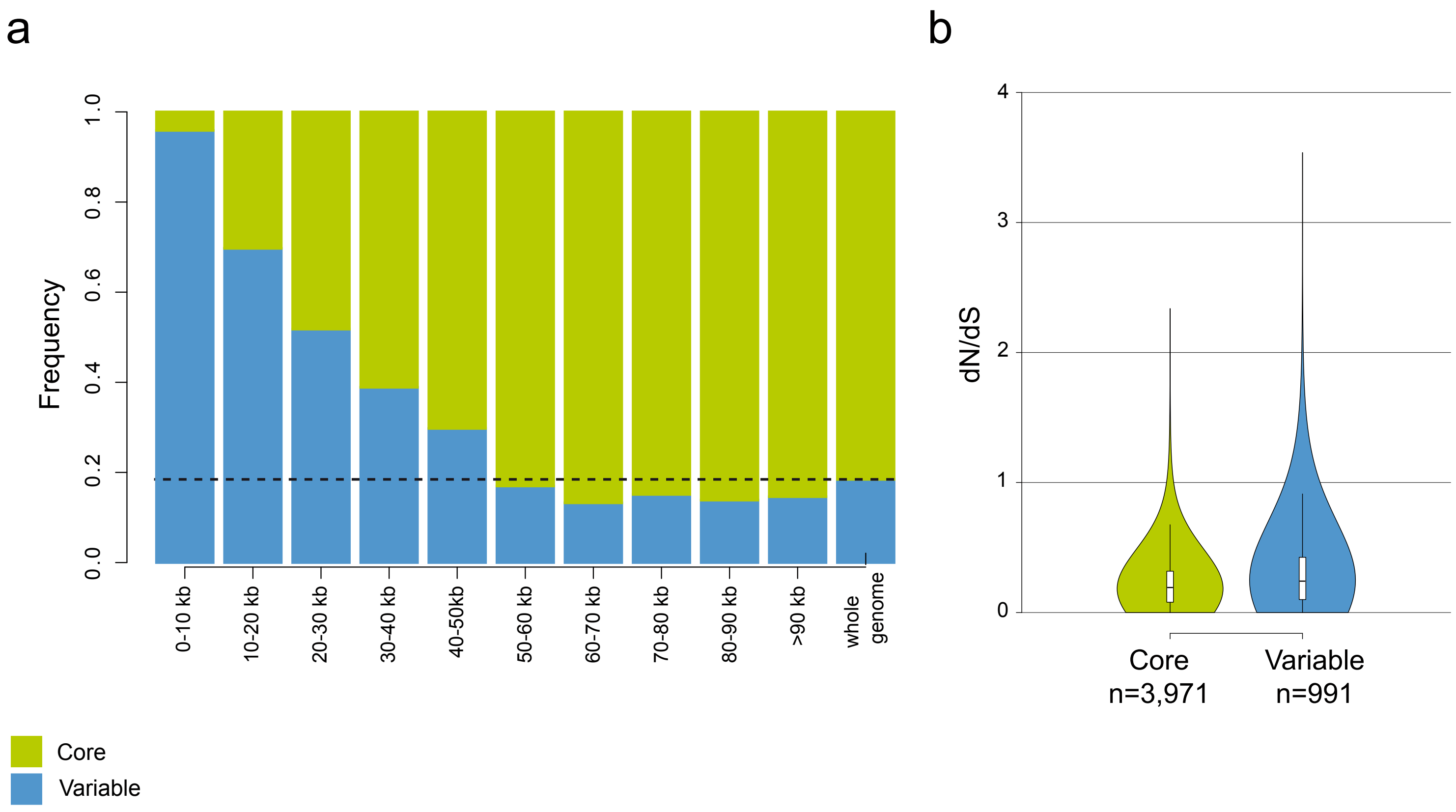

a
b
Frequency
0.0
0.2
0.4
0.6
0.8
1.0
0-10 kb
10-20 kb
20-30 kb
30-40 kb
40-50kb
50-60 kb
60-70 kb
70-80 kb
80-90 kb
>90 kb
whole genome
Core
Variable
dN/dS
0
1
2
3
4
Core
n=3,971
Variable
n=991

Figure S16

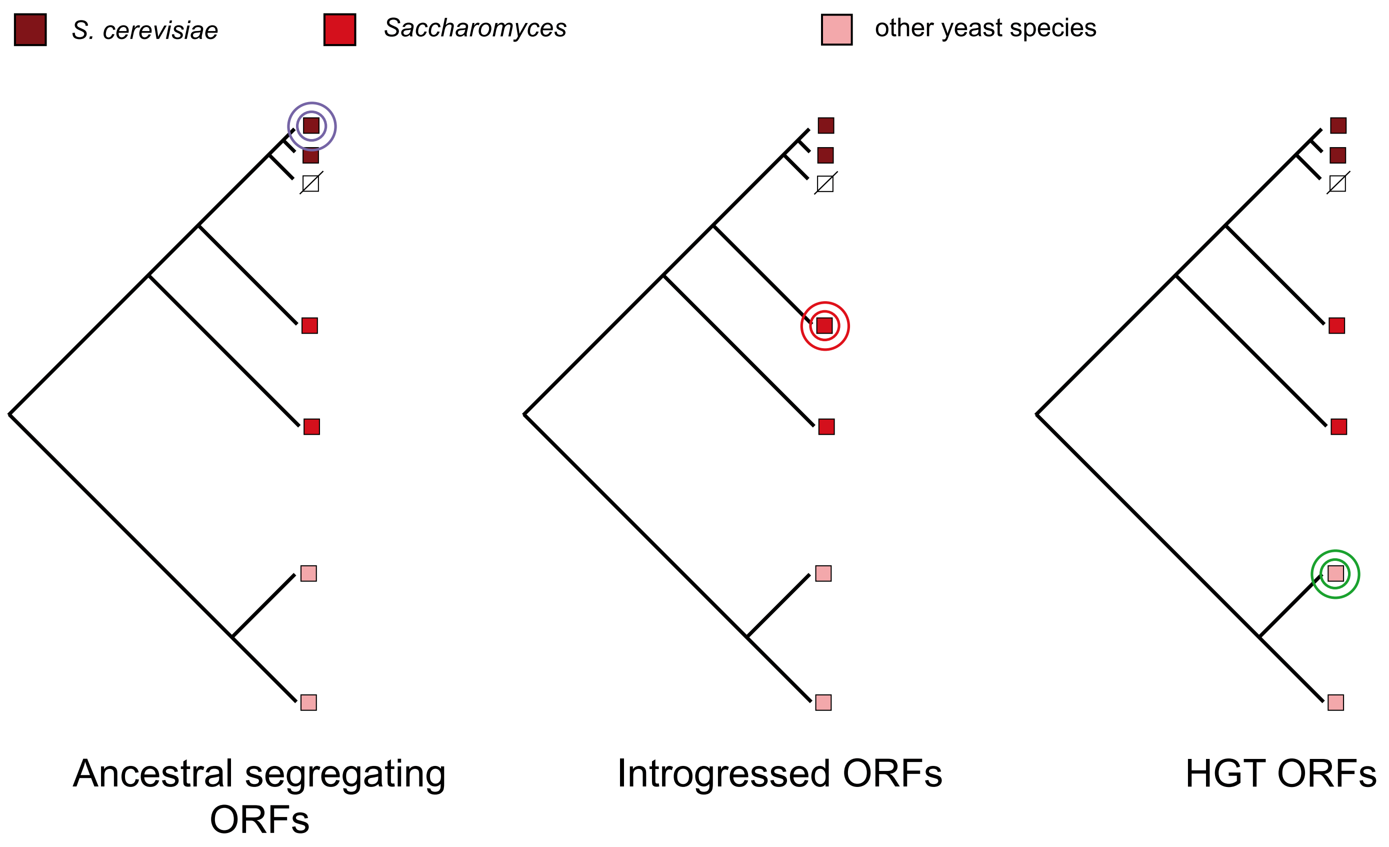
S. cerevisiae
Saccharomyces
other yeast species
Ancestral segregating ORFs
Introgressed ORFs
HGT ORFs

Figure S17

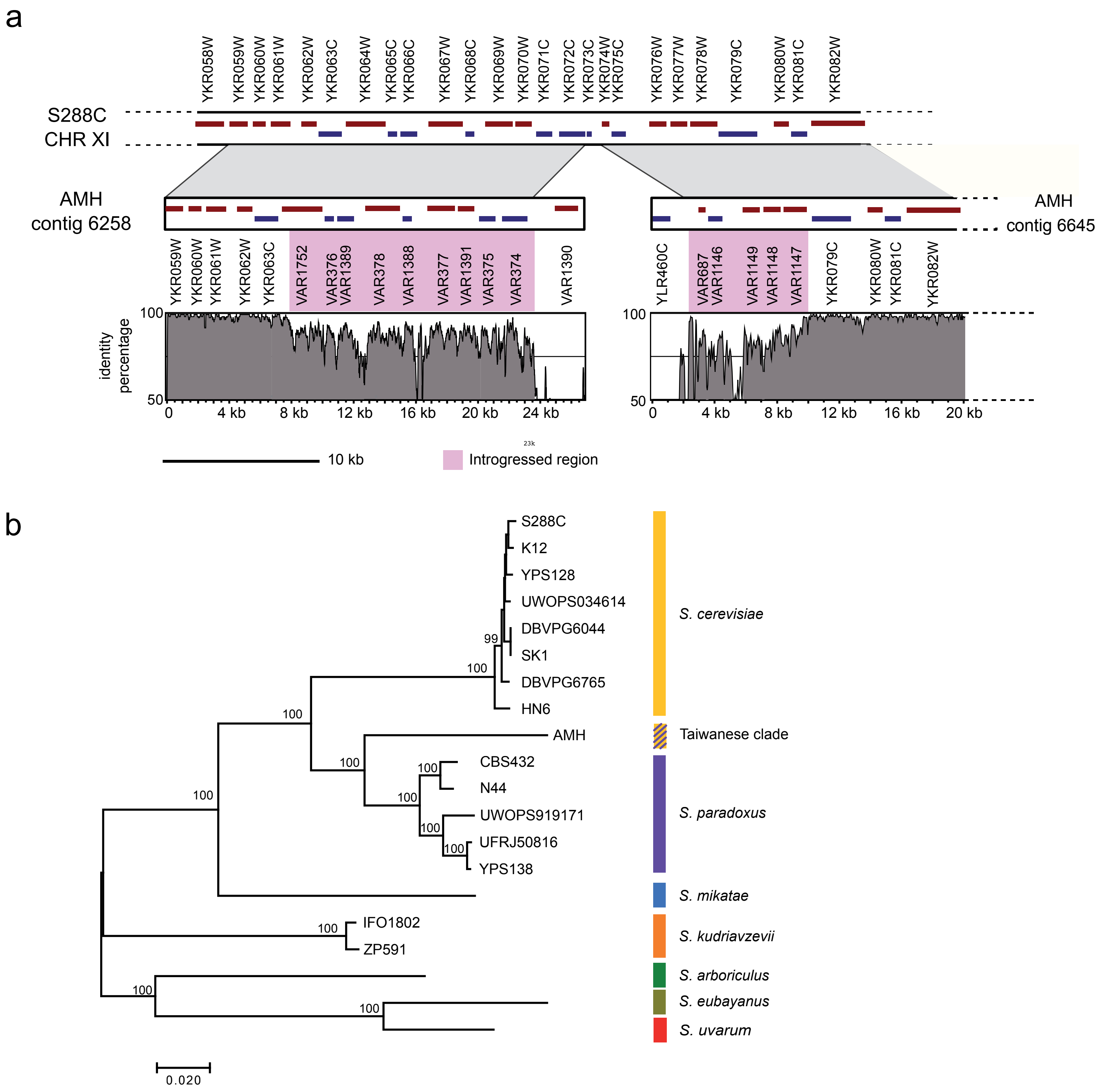
a
YKR058W
YKR059W
YKR060W
YKR061W
YKR062W
YKR063C
YKR064W
YKR065C
YKR066C
YKR067W
YKR068C
YKR069W
YKR070W
YKR071C
YKR072C
YKR073C
YKR074W
YKR075C
YKR076W
YKR077W
YKR078W
YKR079C
YKR080W
YKR081C
YKR082W
S288C
CHR XI
AMH
contig 6258
AMH
contig 6645
YKR059W
YKR060W
YKR061W
YKR062W
YKR063C
VAR1752
VAR376
VAR1389
VAR378
VAR1388
VAR377
VAR1391
VAR375
VAR374
VAR1390
YLR460C
VAR687
VAR1146
VAR1149
VAR1148
VAR1147
YKR079C
YKR080W
YKR081C
YKR082W
identity percentage
100
50
0
4 kb
8 kb
12 kb
16 kb
20 kb
24 kb
10 kb
Introgressed region
b
S288C
K12
YPS128
UWOPS034614
DBVPG6044
SK1
DBVPG6765
HN6
AMH
CBS432
N44
UWOPS919171
UFRJ50816
YPS138
IFO1802
ZP591
99
100
0.020
S. cerevisiae
Taiwanese clade
S. paradoxus
S. mikatae
S. kudriavzevii
S. arboriculus
S. eubayanus
S. uvarum

Figure S18

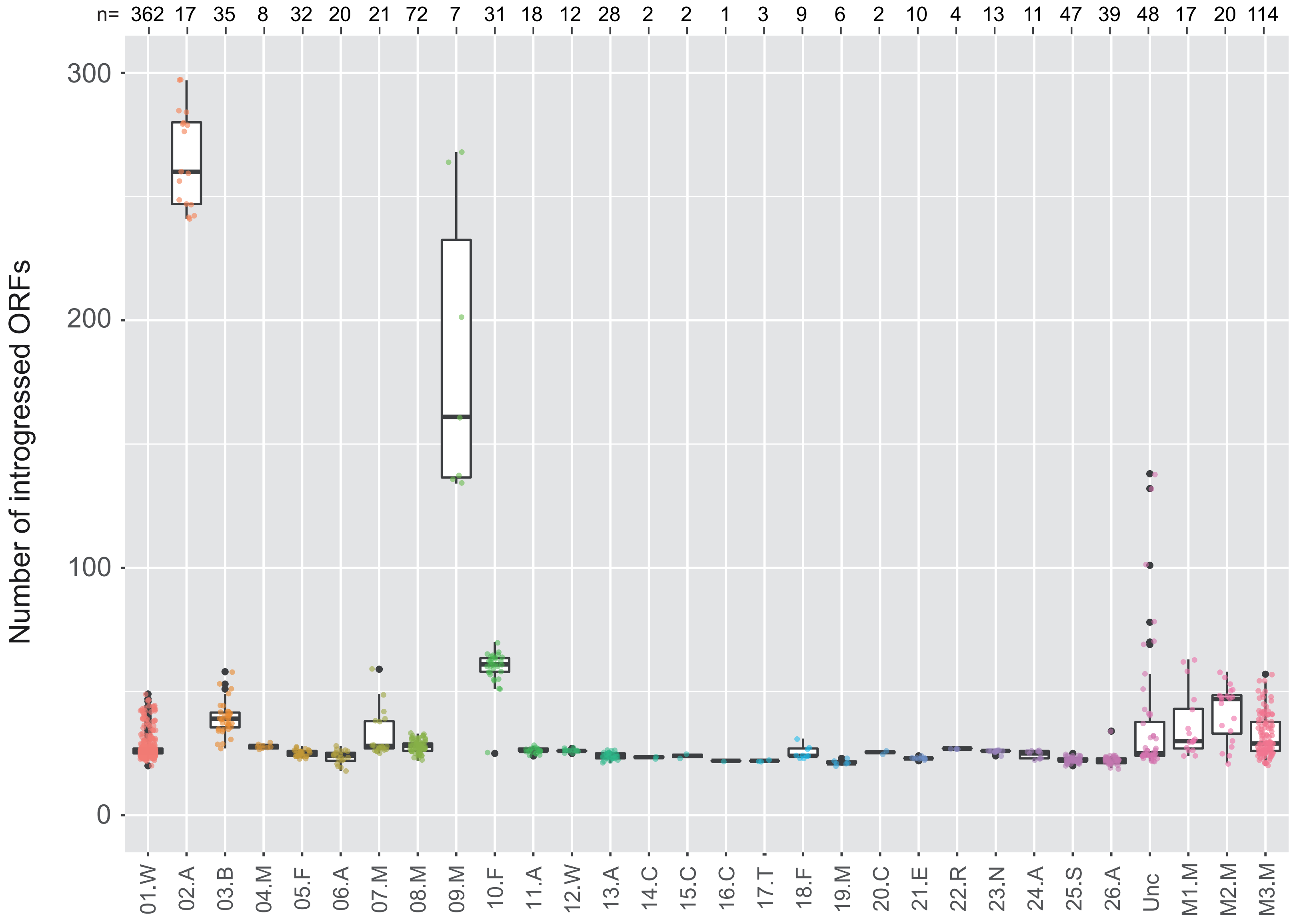

n=
362 17 35 8 32 20 21 72 7 31 18 12 28 2 2 1 3 9 6 2 10 4 13 11 47 39 48 17 20 114
300
200
100
0
Number of introgressed ORFs
01.W
02.A
03.B
04.M
05.F
06.A
07.M
08.M
09.M
10.F
11.A
12.W
13.A
14.C
15.C
16.C
17.T
18.F
19.M
20.C
21.E
22.R
23.N
24.A
25.S
26.A
Unc
M1.M
M2.M
M3.M

Figure S19

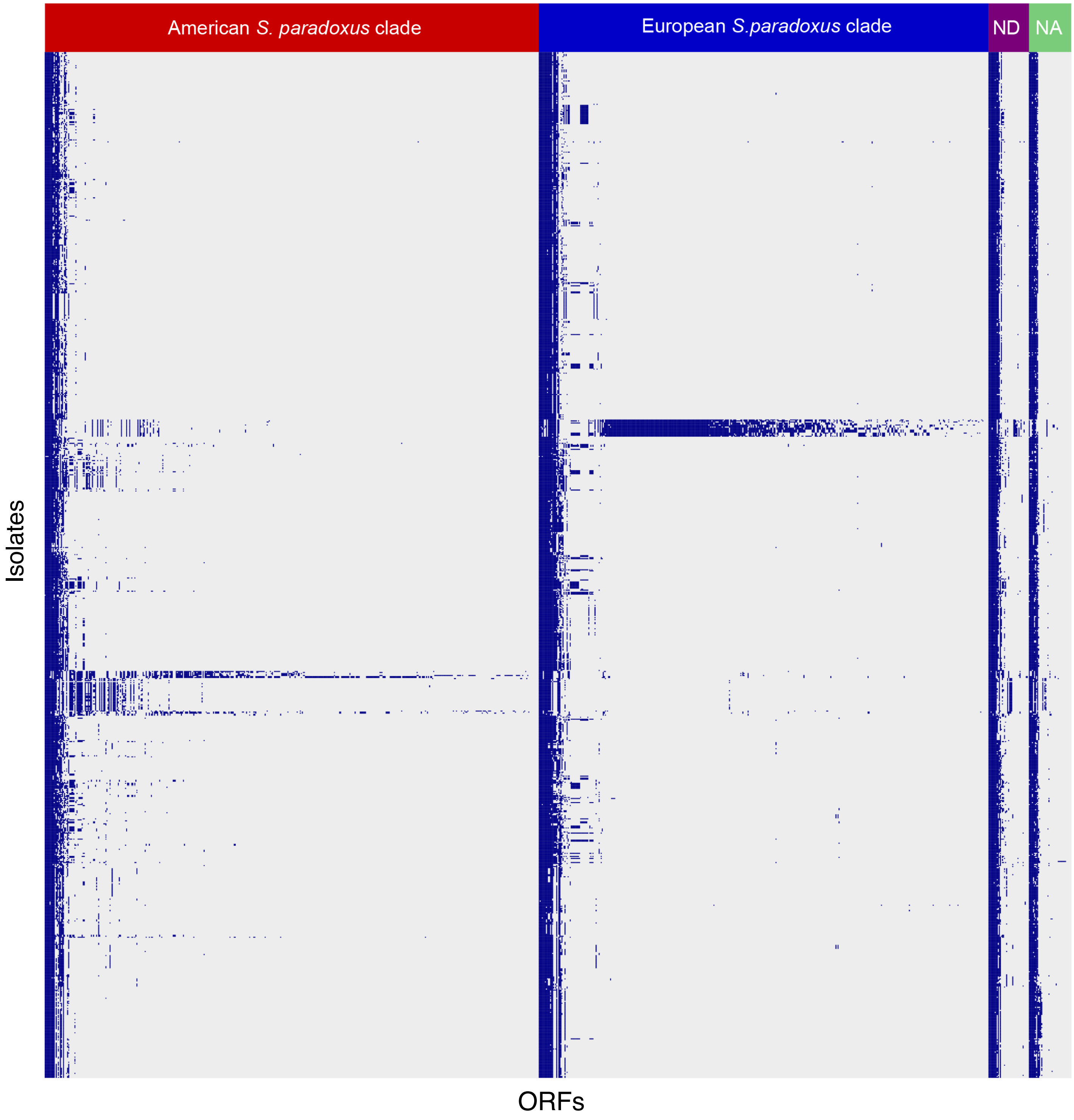
American S. paradoxus clade
European S.paradoxus clade
ND
NA
Isolates
ORFs

Figure S20

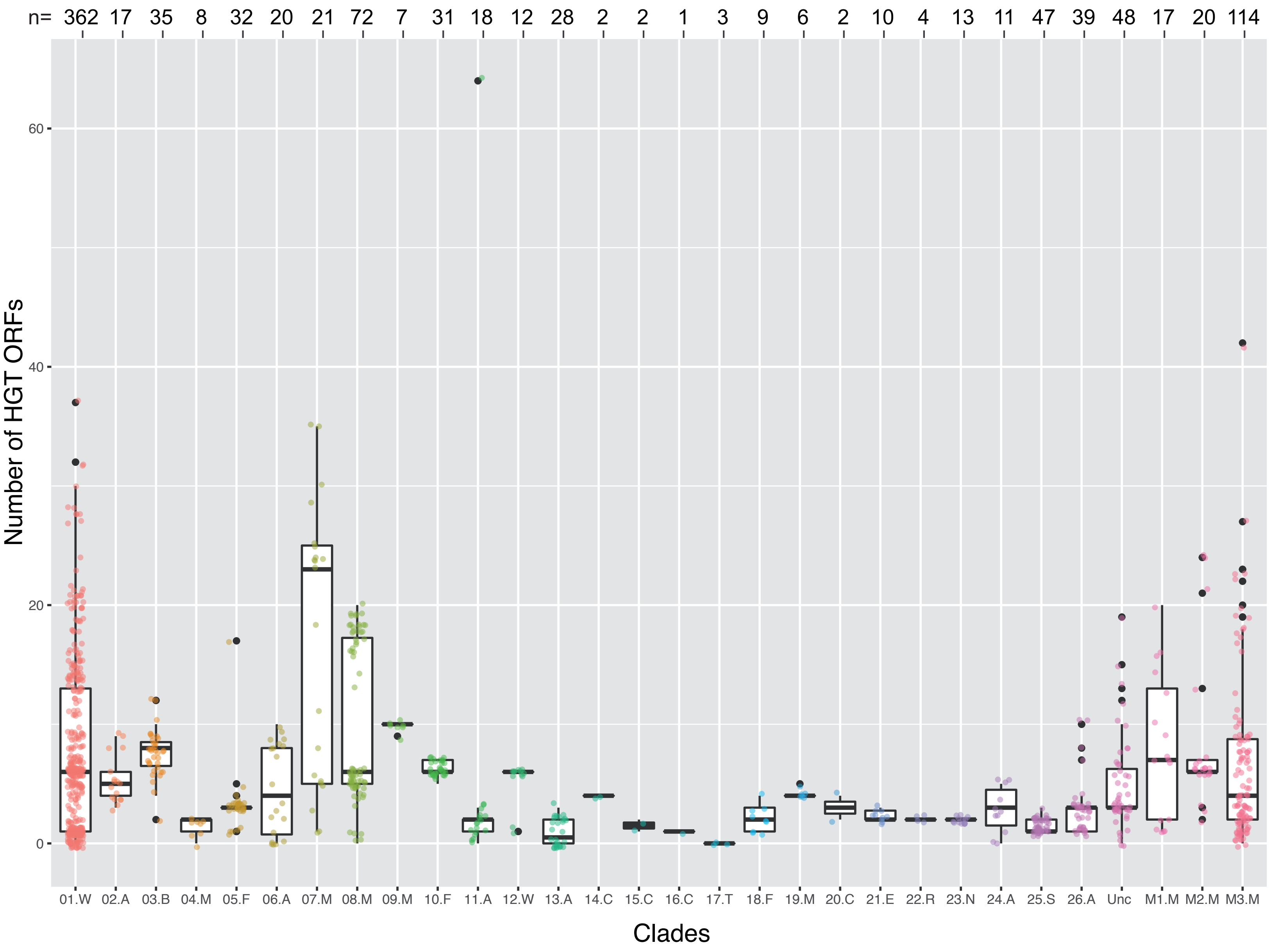
n= 362 17 35 8 32 20 21 72 7 31 18 12 28 2 2 1 3 9 6 2 10 4 13 11 47 39 48 17 20 114
Number of HGT ORFs
0
20
40
60
01.W 02.A 03.B 04.M 05.F 06.A 07.M 08.M 09.M 10.F 11.A 12.W 13.A 14.C 15.C 16.C 17.T 18.F 19.M 20.C 21.E 22.R 23.N 24.A 25.S 26.A Unc M1.M M2.M M3.M
Clades

Figure S21

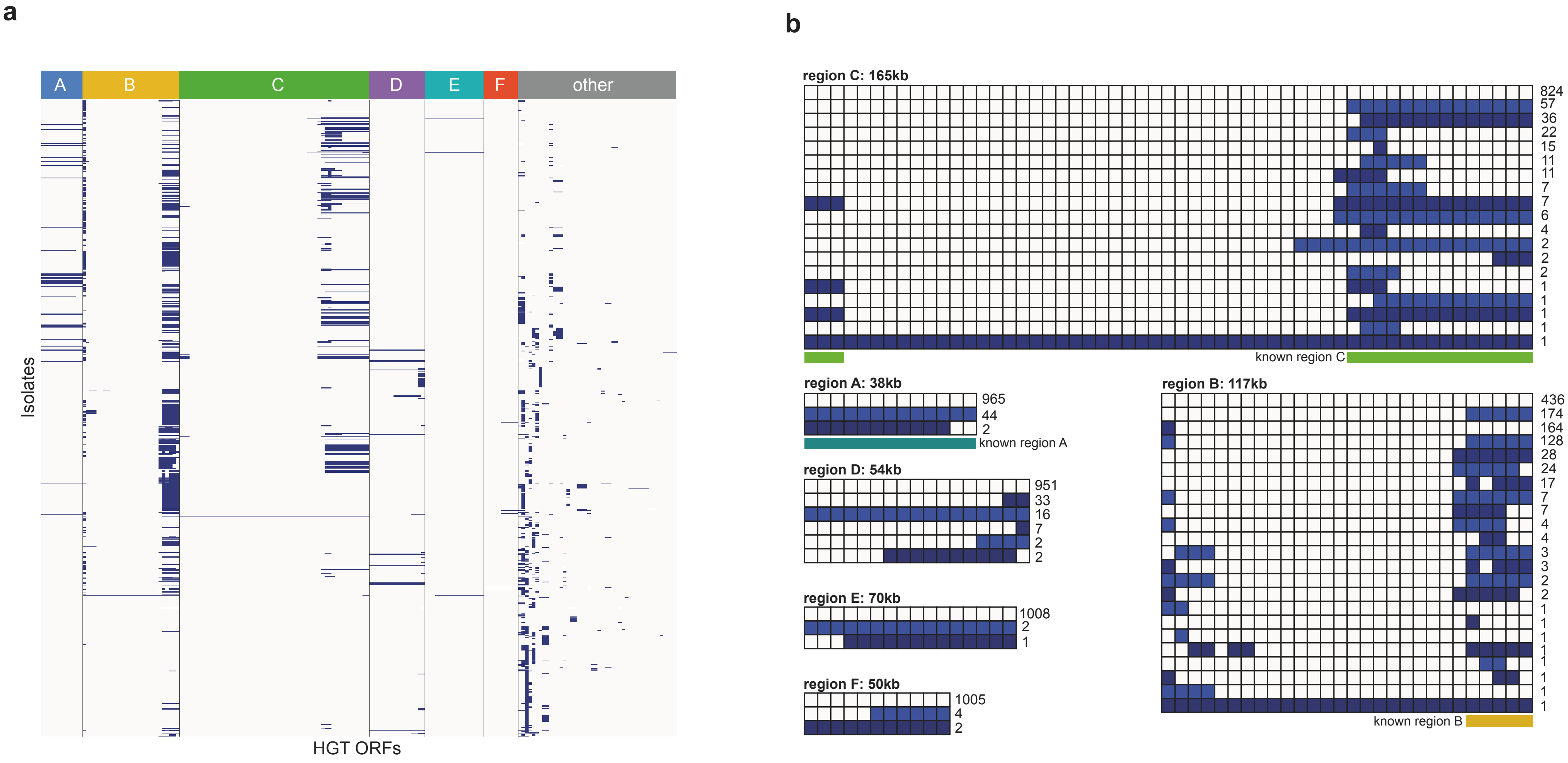
a
A
B
C
D
E
F
other
Isolates
HGT ORFs
b
region C: 165kb
824
57
36
22
15
11
11
7
7
6
4
2
2
2
1
1
1
1
1
known region C
region A: 38kb
965
44
2
known region A
region D: 54kb
951
33
16
7
2
2
region E: 70kb
1008
2
1
region F: 50kb
1005
4
2
region B: 117kb
436
174
164
128
28
24
17
7
7
4
4
3
3
2
2
1
1
1
1
1
1
1
1
known region B

Figure S22

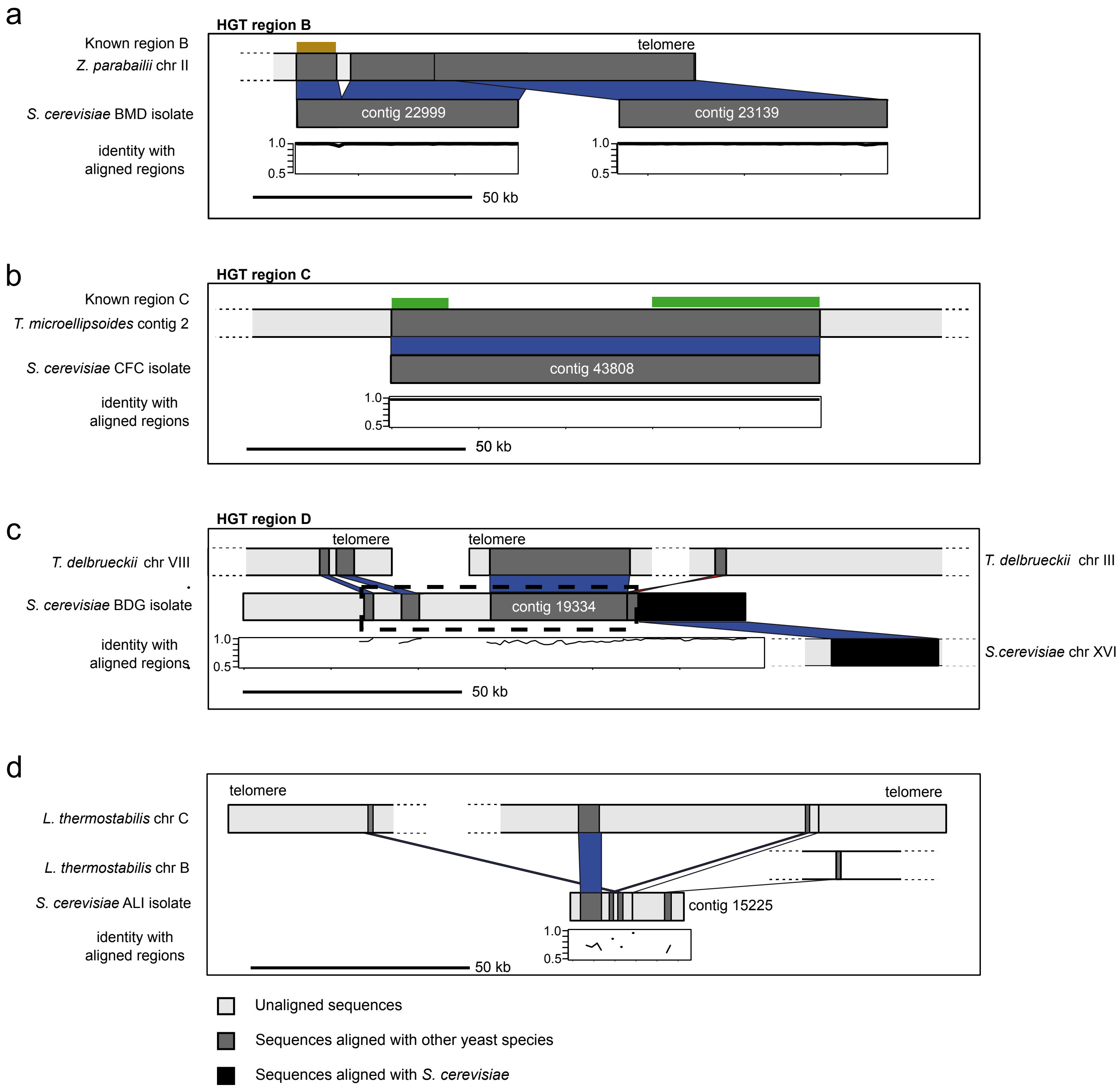

a
HGT region B
Known region B
Z. parabailii chr II
telomere
S. cerevisiae BMD isolate
contig 22999
contig 23139
identity with aligned regions
1.0
0.5
50 kb
b
HGT region C
Known region C
T. microellipsoides contig 2
S. cerevisiae CFC isolate
contig 43808
identity with aligned regions
1.0
0.5
50 kb
c
HGT region D
telomere
telomere
T. delbrueckii chr VIII
T. delbrueckii chr III
S. cerevisiae BDG isolate
contig 19334
identity with aligned regions
1.0
0.5
S.cerevisiae chr XVI
50 kb
d
telomere
telomere
L. thermostabilis chr C
L. thermostabilis chr B
S. cerevisiae ALI isolate
contig 15225
identity with aligned regions
1.0
0.5
50 kb
Unaligned sequences
Sequences aligned with other yeast species
Sequences aligned with S. cerevisiae

Figure S23

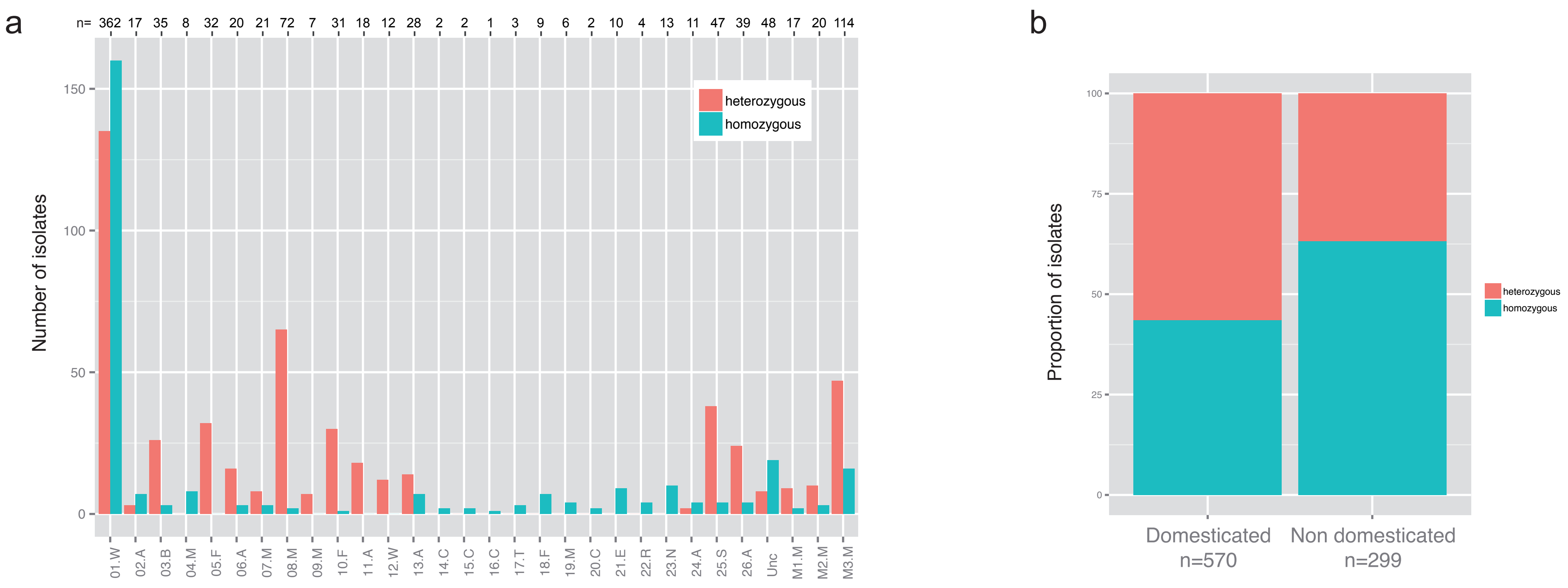
a
n= 362 17 35 8 32 20 21 72 7 31 18 12 28 2 2 1 3 9 6 2 10 4 13 11 47 39 48 17 20 114
Number of isolates
150
100
50
0
heterozygous
homozygous
01.W 02.A 03.B 04.M 05.F 06.A 07.M 08.M 09.M 10.F 11.A 12.W 13.A 14.C 15.C 16.C 17.T 18.F 19.M 20.C 21.E 22.R 23.N 24.A 25.S 26.A Unc M1.M M2.M M3.M
b
Proportion of isolates
100
75
50
25
0
heterozygous
homozygous
Domesticated
n=570
Non domesticated
n=299

Figure S24

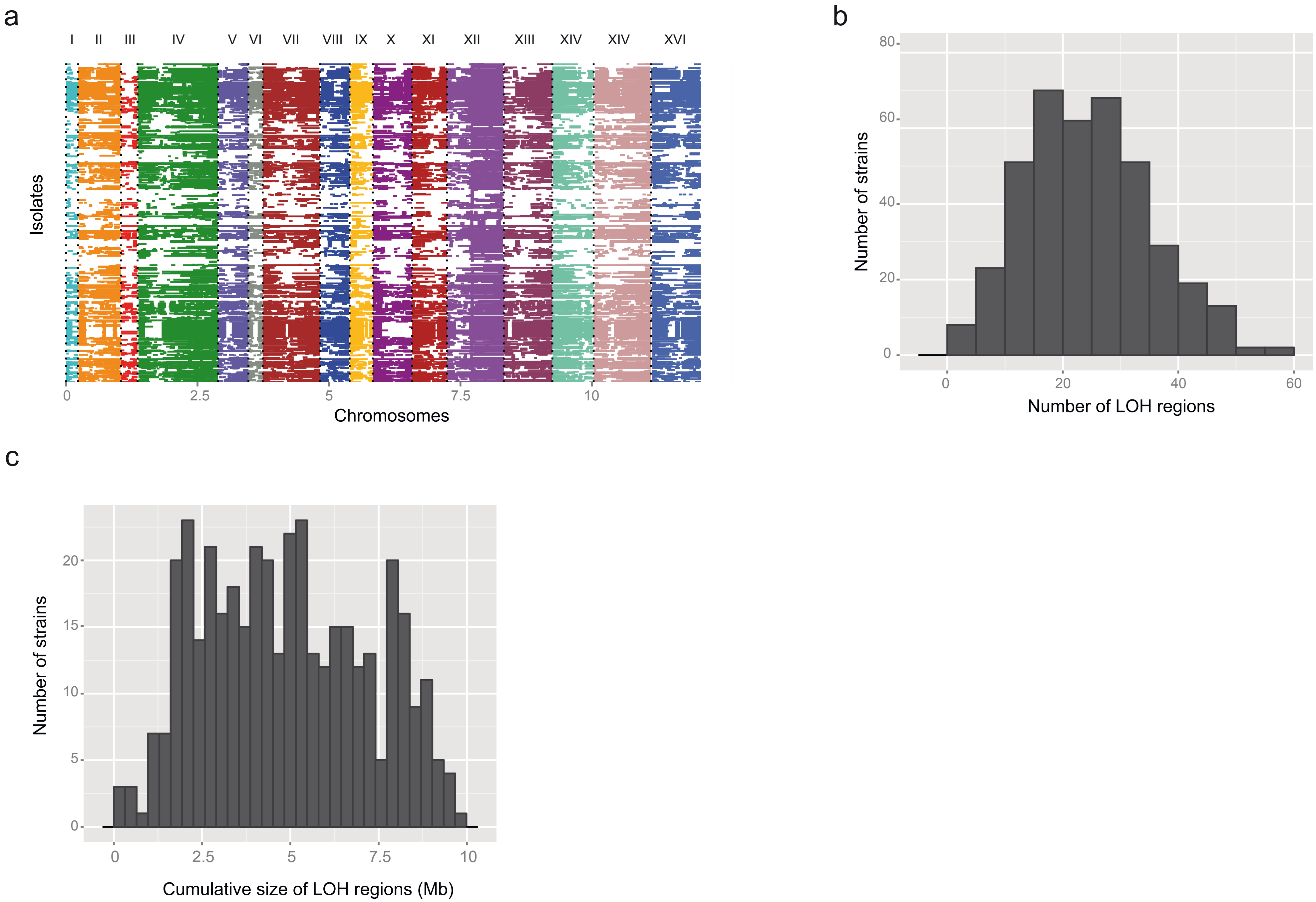
a
I
II
III
IV
V
VI
VII
VIII
IX
X
XI
XII
XIII
XIV
XIV
XVI
Isolates
0
2.5
5
7.5
10
Chromosomes
b
80
60
40
20
0
Number of strains
0
20
40
60
Number of LOH regions
c
20
15
10
5
0
Number of strains
0
2.5
5
7.5
10
Cumulative size of LOH regions (Mb)

Figure S25

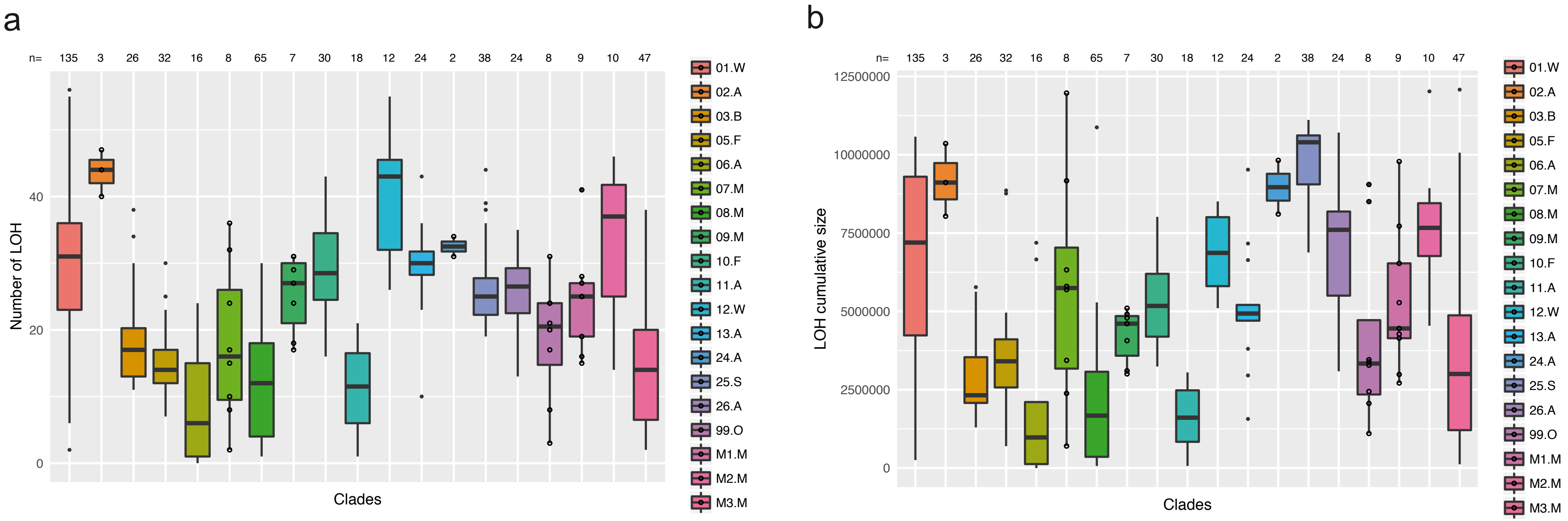

a
n= 135 3 26 32 16 8 65 7 30 18 12 24 2 38 24 8 9 10 47
Number of LOH
0
20
40
Clades
01.W
02.A
03.B
05.F
06.A
07.M
08.M
09.M
10.F
11.A
12.W
13.A
24.A
25.S
26.A
99.O
M1.M
M2.M
M3.M
b
n= 135 3 26 32 16 8 65 7 30 18 12 24 2 38 24 8 9 10 47
LOH cumulative size
0
2500000
5000000
7500000
10000000
12500000
Clades

Figure S26

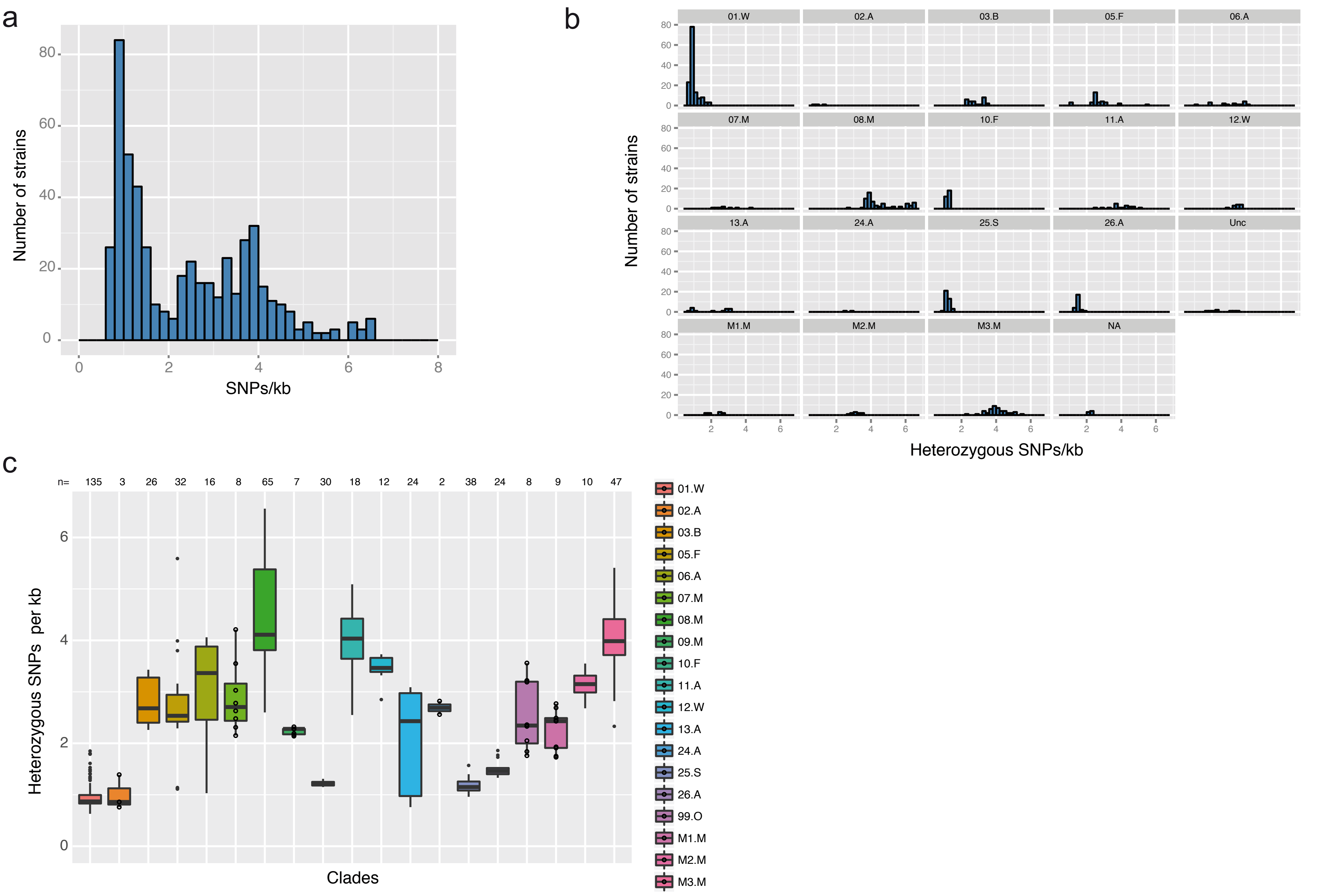
a
Number of strains
SNPs/kb
b
Number of strains
Heterozygous SNPs/kb
01.W
02.A
03.B
05.F
06.A
07.M
08.M
10.F
11.A
12.W
13.A
24.A
25.S
26.A
Unc
M1.M
M2.M
M3.M
NA
c
n= 135 3 26 32 16 8 65 7 30 18 12 24 2 38 24 8 9 10 47
Heterozygous SNPs per kb
Clades
01.W
02.A
03.B
05.F
06.A
07.M
08.M
09.M
10.F
11.A
12.W
13.A
24.A
25.S
26.A
99.O
M1.M
M2.M
M3.M

Figure S27

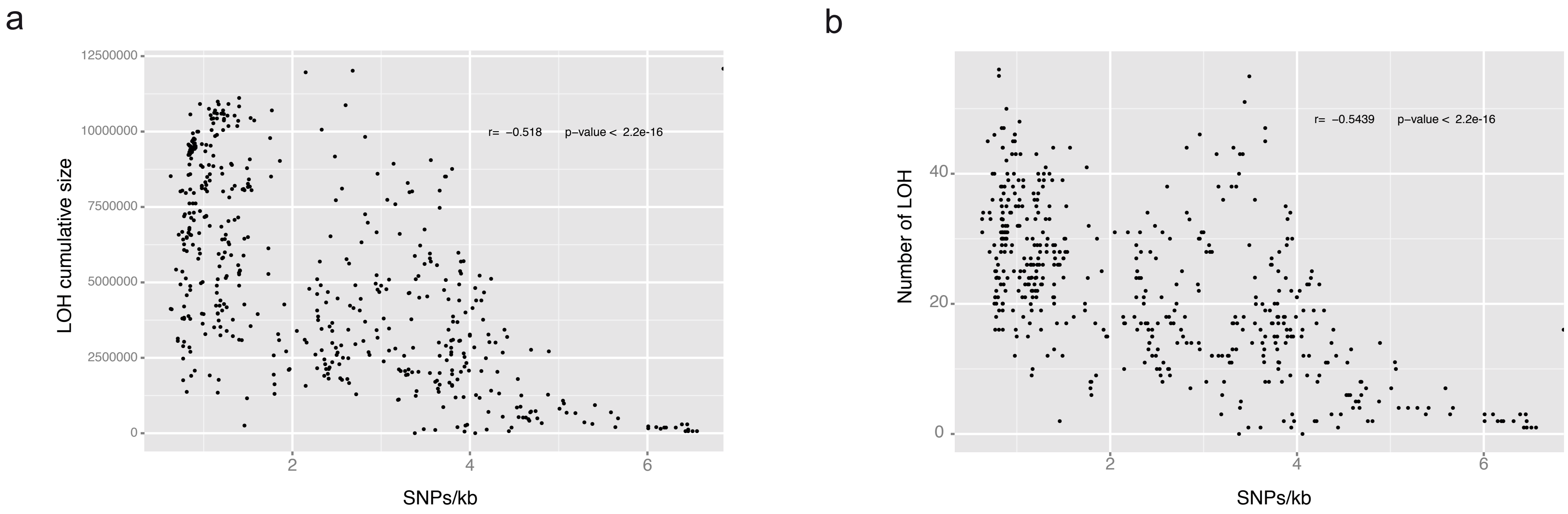
a
12500000
10000000
7500000
5000000
2500000
0
LOH cumulative size
2
4
6
SNPs/kb
r= −0.518 p−value < 2.2e-16
b
40
20
0
Number of LOH
2
4
6
SNPs/kb
r= −0.5439 p−value < 2.2e-16

Figure S28

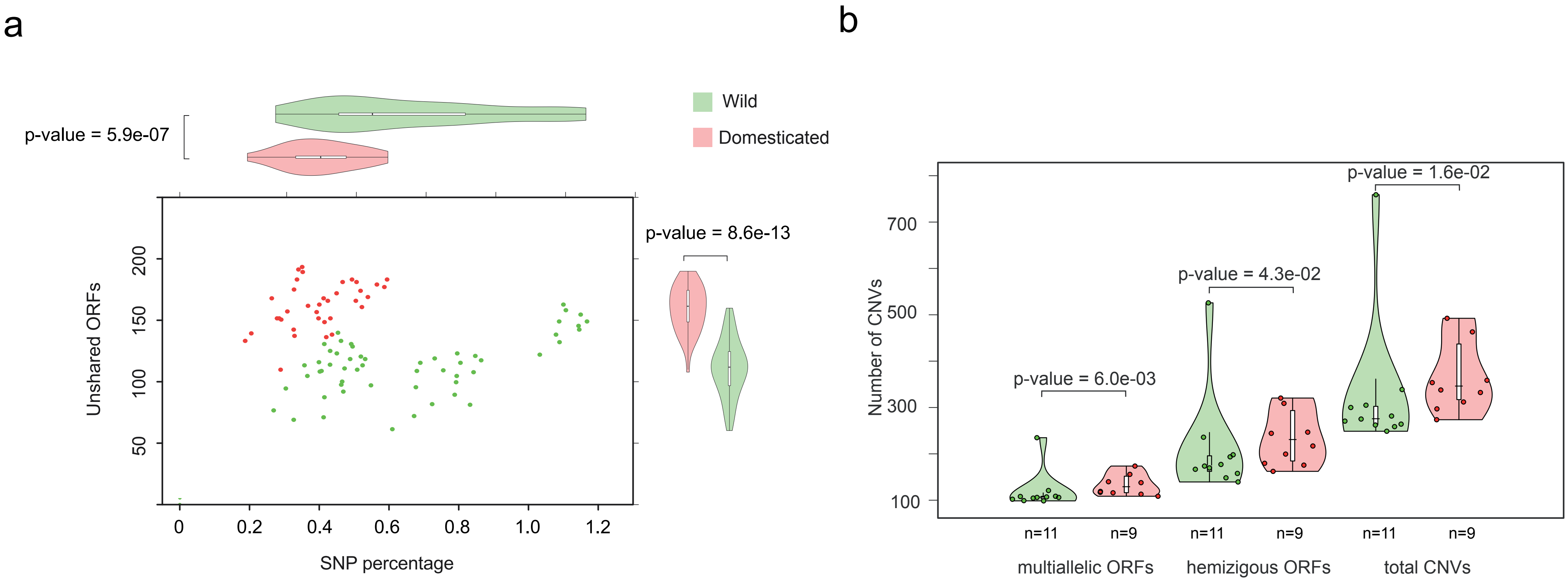
a
p-value = 5.9e-07
Wild
Domesticated
p-value = 8.6e-13
Unshared ORFs
50
100
150
200
0
0.2
0.4
0.6
0.8
1.0
1.2
SNP percentage
b
Number of CNVs
100
300
500
700
p-value = 6.0e-03
p-value = 4.3e-02
p-value = 1.6e-02
n=11
n=9
n=11
n=9
n=11
n=9
multiallelic ORFs
hemizigous ORFs
total CNVs

Figure S29

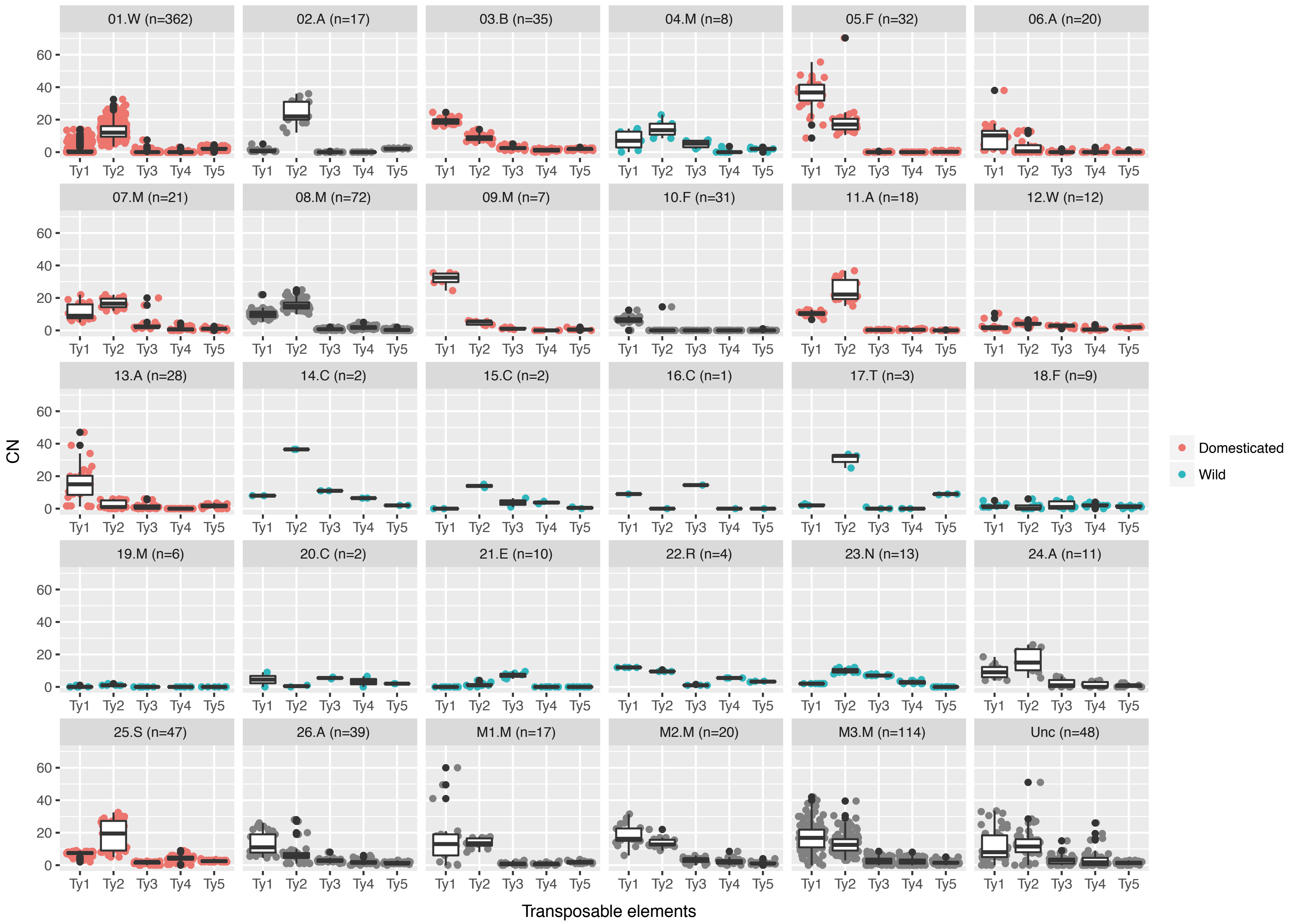
01.W (n=362)
02.A (n=17)
03.B (n=35)
04.M (n=8)
05.F (n=32)
06.A (n=20)
07.M (n=21)
08.M (n=72)
09.M (n=7)
10.F (n=31)
11.A (n=18)
12.W (n=12)
13.A (n=28)
14.C (n=2)
15.C (n=2)
16.C (n=1)
17.T (n=3)
18.F (n=9)
19.M (n=6)
20.C (n=2)
21.E (n=10)
22.R (n=4)
23.N (n=13)
24.A (n=11)
25.S (n=47)
26.A (n=39)
M1.M (n=17)
M2.M (n=20)
M3.M (n=114)
Unc (n=48)
CN
0
20
40
60
Ty1
Ty2
Ty3
Ty4
Ty5
Transposable elements
Domesticated
Wild

Figure S30

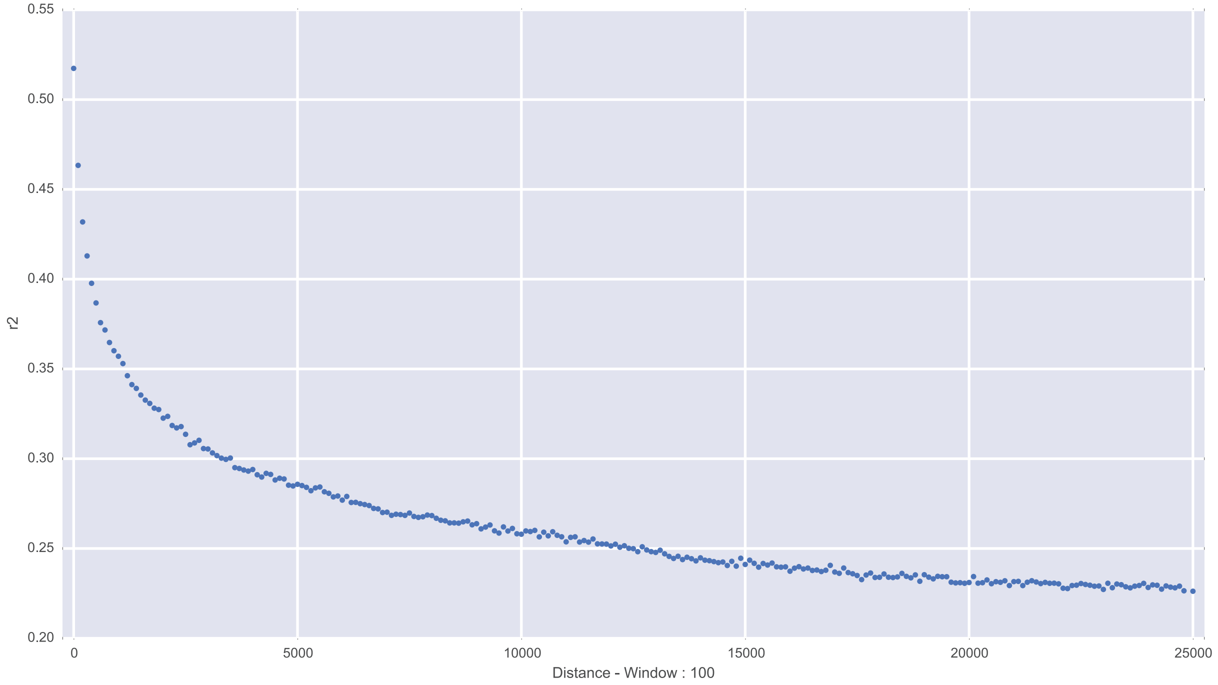

0.55
0.50
0.45
0.40
0.35
0.30
0.25
0.20
r2
0
5000
10000
15000
20000
25000
Distance - Window : 100

Figure S31

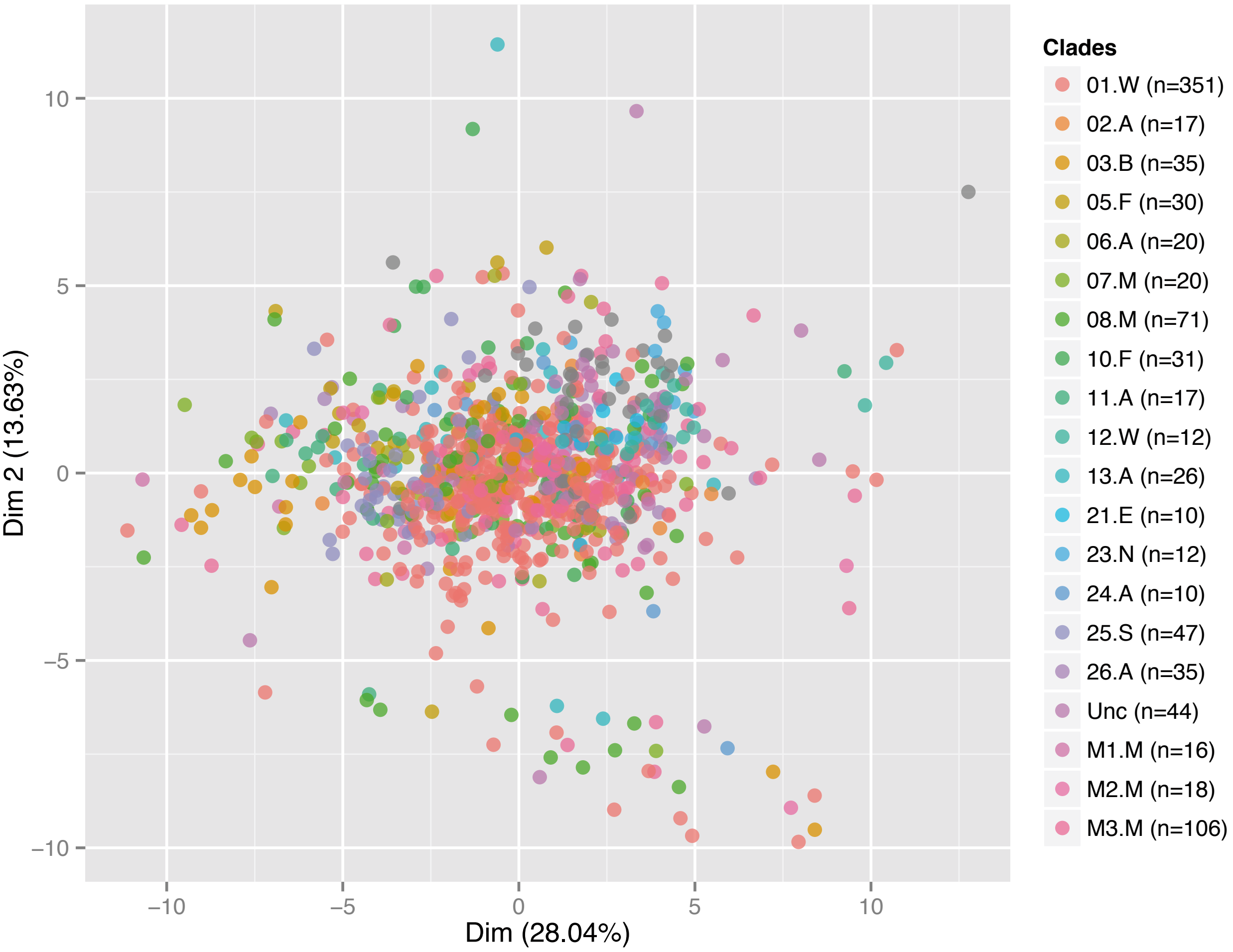
Clades
01.W (n=351)
02.A (n=17)
03.B (n=35)
05.F (n=30)
06.A (n=20)
07.M (n=20)
08.M (n=71)
10.F (n=31)
11.A (n=17)
12.W (n=12)
13.A (n=26)
21.E (n=10)
23.N (n=12)
24.A (n=10)
25.S (n=47)
26.A (n=35)
Unc (n=44)
M1.M (n=16)
M2.M (n=18)
M3.M (n=106)
Dim 2 (13.63%)
Dim (28.04%)
10
5
0
−5
−10

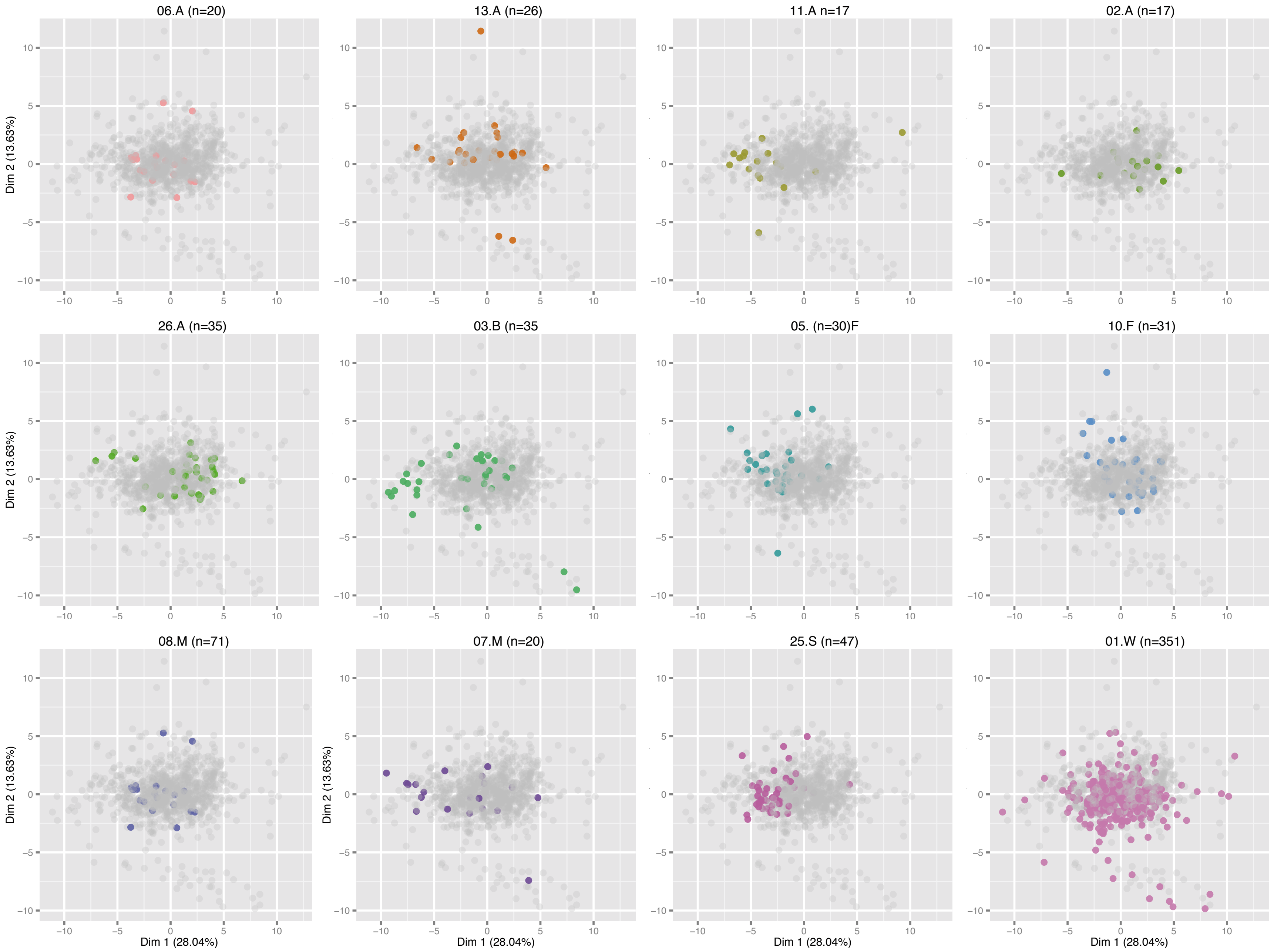

Figure S31. Principal component projection (PCA) using growth ratio as in Fig. S30. Here, each graph is meant to highlight a clade against the total population. For each clade, n indicates the number of isolates.

Figure S33

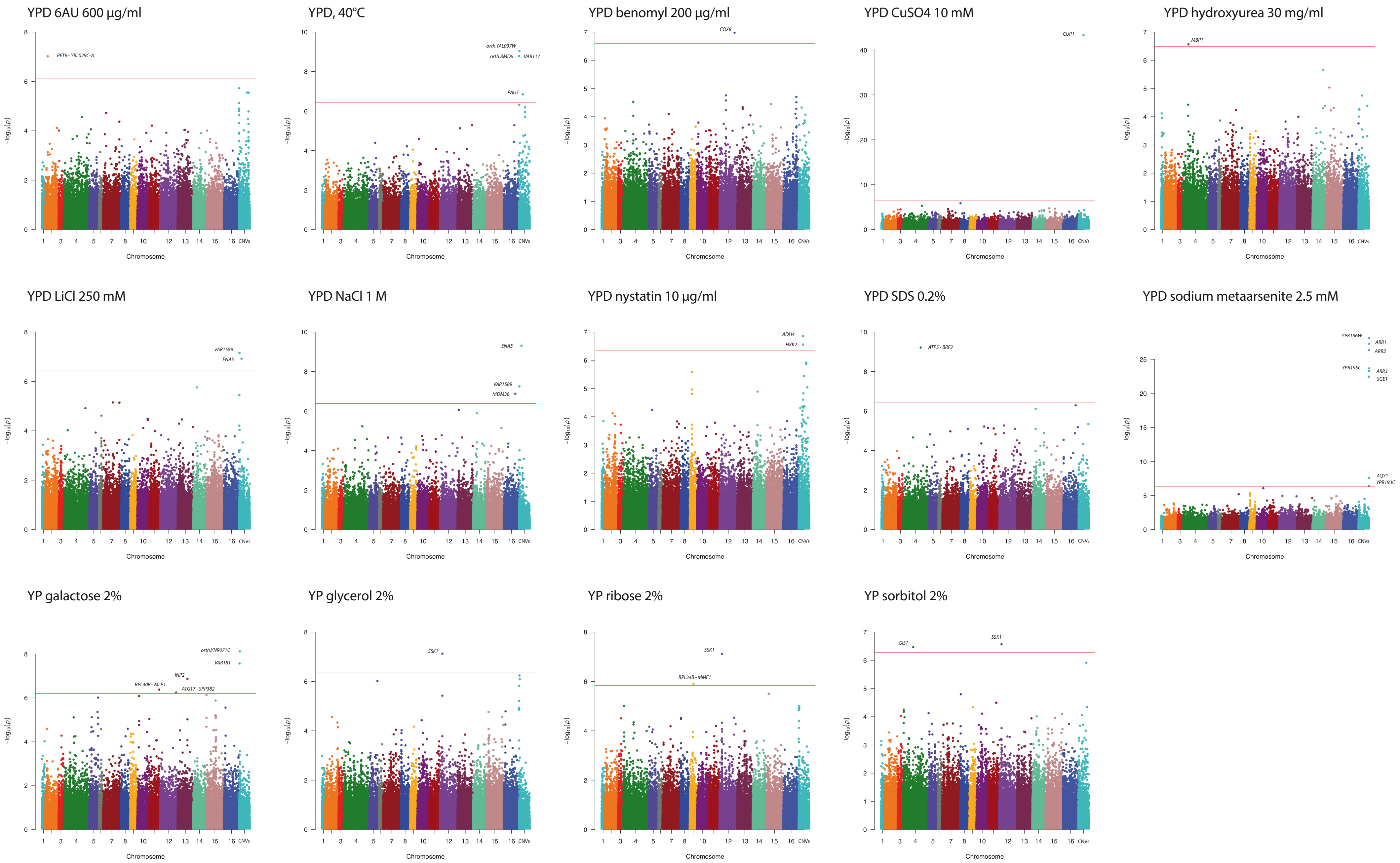
YPD 6AU 600 µg/ml
PET9 - YBL029C-A
YPD, 40°C
orth.YAL037W
orth.RMD6
VAR117
PAU5
YPD benomyl 200 µg/ml
COX8
YPD CuSO4 10 mM
CUP1
YPD hydroxyurea 30 mg/ml
MBP1
YPD LiCl 250 mM
VAR1589
ENA5
YPD NaCl 1 M
ENA5
VAR1589
MDM36
YPD nystatin 10 µg/ml
ADH4
HXK2
YPD SDS 0.2%
ATP5 - BRF2
YPD sodium metaarsenite 2.5 mM
YPR196W
ARR1
ARR2
YPR195C
ARR3
SGE1
AQY1
YPR193C
YP galactose 2%
orth.YNR071C
VAR181
INP2
RPL40B - MLP1
ATG17 - SPP382
YP glycerol 2%
SSK1
YP ribose 2%
SSK1
RPL34B - MMF1
YP sorbitol 2%
GIS1
SSK1
Chromosome
−log10(p)

Figure S34

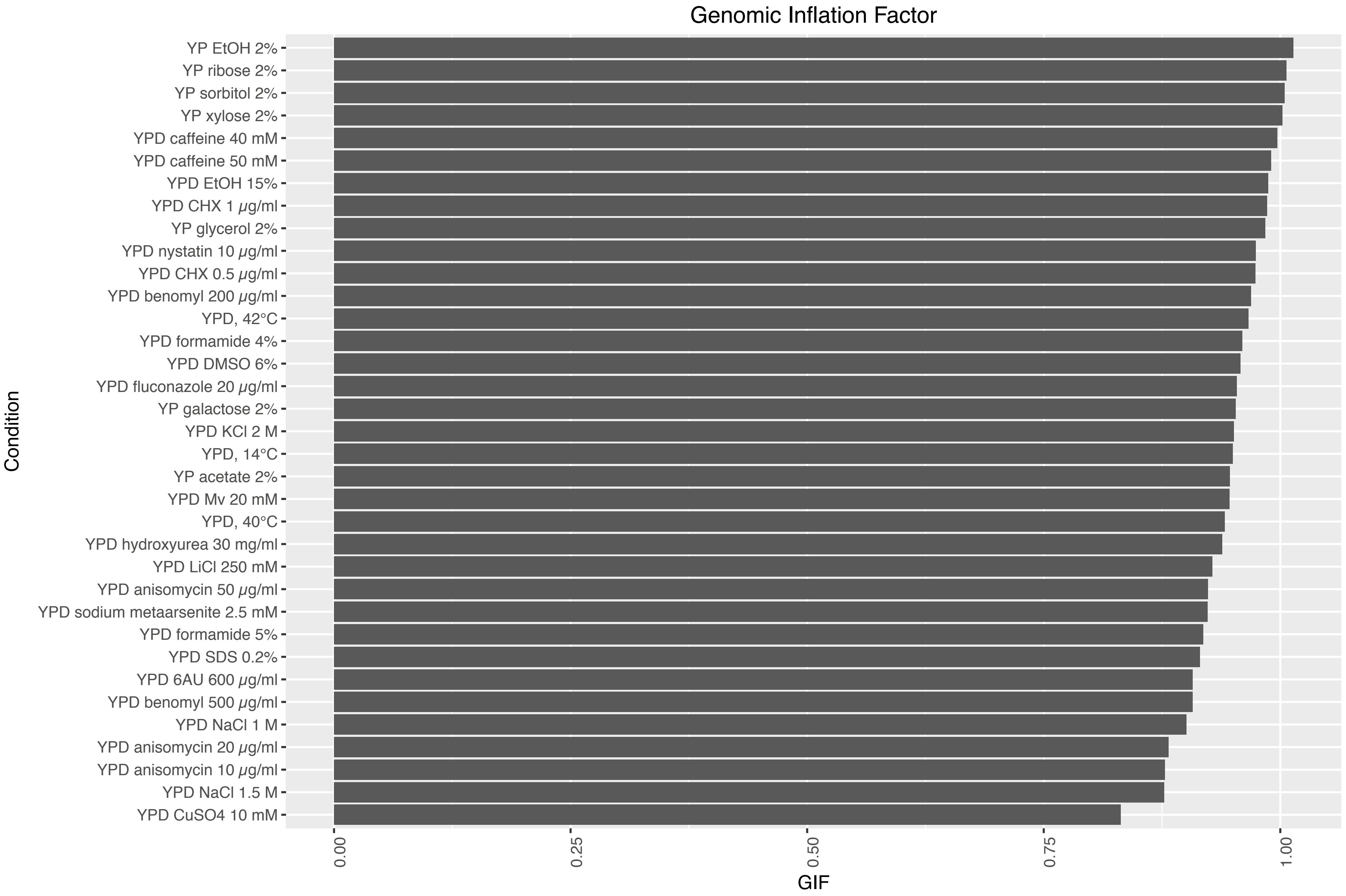

Genomic Inflation Factor
Condition
YP EtOH 2%
YP ribose 2%
YP sorbitol 2%
YP xylose 2%
YPD caffeine 40 mM
YPD caffeine 50 mM
YPD EtOH 15%
YPD CHX 1 μg/ml
YP glycerol 2%
YPD nystatin 10 μg/ml
YPD CHX 0.5 μg/ml
YPD benomyl 200 μg/ml
YPD, 42°C
YPD formamide 4%
YPD DMSO 6%
YPD fluconazole 20 μg/ml
YP galactose 2%
YPD KCl 2 M
YPD, 14°C
YP acetate 2%
YPD Mv 20 mM
YPD, 40°C
YPD hydroxyurea 30 mg/ml
YPD LiCl 250 mM
YPD anisomycin 50 μg/ml
YPD sodium metaarsenite 2.5 mM
YPD formamide 5%
YPD SDS 0.2%
YPD 6AU 600 μg/ml
YPD benomyl 500 μg/ml
YPD NaCl 1 M
YPD anisomycin 20 μg/ml
YPD anisomycin 10 μg/ml
YPD NaCl 1.5 M
YPD CuSO4 10 mM
0.00
0.25
0.50
0.75
1.00
GIF

Supplementary Figure legends

Figure S1. Overview of the 1,011 sequenced isolates. a, Geographical origins of the isolates with circle sizes proportional to isolates number. **b,** Ecological origins of the isolates. Number in parentheses indicate isolates per category. **c,** Genome sequencing coverage of the 1,011 *S. cerevisiae* isolates. Sequencing coverage distribution of the 918 sequenced isolates in the frame of this project (blue) and for the 93 previously sequenced genomes (red).

Figure S2. Frequency spectrum of SNPs in the 1,011 genomes. Minor allele frequency (MAF) of polymorphisms was determined for SNPs with different location in the genome and functional annotation.

Figure S3. Small indels across the 1,011 genomes. A total of 125,701 indels (up to 50 bp) were detected across the 1,011 genomes. **a,** Frequency spectrum of small-scale indels. Distribution of the indel size in non-coding (**b**) and coding (**c**) regions.

Figure S4. CNV distribution across isolates and ORFs. a, Distribution of median CN values across a different set of ORFs. Plasmids, mitochondria, and repetitive elements show the highest CN, while ORFs located in the core chromosome have virtually no CNV. n indicates the number of ORFs in each plot. **b,** CNV occurs in the majority of the ORFs, with only 1,242 ORFs without increase of CN in any strain. However, most of ORF CNVs (n=7,182) occur at low frequencies (less than 5% among the isolates). **c,** Histogram of the maximal CN per each pangenomic ORF. Most of the ORFs never exceed CN = 2 (n=7,042).

Figure S5. Fitness trait distribution patterns. Distribution of the 971 phenotyped isolates for each trait (bin size = 0.06; growth conditions are described in Table S2). Most of the traits vary continuously across the population and are complex, whereas a small number of them shows a bimodal distribution characteristic of a Mendelian inheritance (*e.g.* $CuSO_4$).

Figure S6. Narrow-sense heritability of fitness traits. The genome-wide heritability (GW heritability) ranges from 0.47 to 0.9 with an average of 0.69. Growth conditions are described in Table S2).

Figure S7. PCA based on presence of variable ORFs in all the 1,011 isolates. The plots show the position of each strain projected onto components of the PCA based on presence/absence of variable ORFs. **a-c,** Colours and symbols indicate the strain clades. The 1st, 2nd and 3rd components describe, respectively, the strain order in the phylogenetic tree, the introgressions in the alpechin clade (02.A) as well as the introgressions in the Mexican agave (09.M) and French Guiana (10.F) clades. **d,** Colours indicate wild and domesticated clades while the symbols are the same as in the other panels. All the wild clades, including the Mediterranean oak, group together and are in the top right corner. The only domesticated clade that clusters with the wild isolates is the West African cocoa subpopulation.

Figure S8. ***S. cerevisiae* population structure.** The underlying population structure inferred using the software ADMIXTURE using a varying number of ancestry components (from 2 to 17). Strains are ordered based on the phylogenetic tree. The diversity among the mosaic groups of strains is clear with a much higher ancestral complexity of the Mosaic region 3.

Figure S9. Population genomics of the natural 2µ plasmid. a, NJ tree built using representative plasmid sequence variants. Class D is only detected in the most diverged Taiwanese lineage and likely derived from another Saccharomyces species. The classes B and B* are not included since they are recombinant forms of classes A and C. **b,** Distribution of the different plasmid classes in the sequenced strains. **c,** Broad range of plasmid copy number in the different sequence variants. n indicates the number of plasmid CN for each plot. Centre lines, median; boxes, interquartile range

(IQR); whiskers, 1.5× IQR. Data points beyond the whiskers are outliers. See supplementary Table 21 for confidence intervals.

Figure S10. Principal components calculated using SNP genotypes of CHN I-V and Taiwanese isolates. The plot shows the position of all other sequenced isolates (n=1,011) projected onto the resulting space. The non-Chinese lineages all project onto the same part of the space. This suggests a scenario with a single, shared Chinese origin of all non-Chinese *S. cerevisiae* strains, rather than a scenario where multiple, different Chinese lineages contributed to non-Chinese strains in different parts of the world.

Figure S11. Ploidy level variation across the subpopulations. The frequency of the ploidy level was represented for each of the 26 subpopulations as well as for the 3 mosaic groups. Most of the subpopulations have only diploid isolates. However, an enrichment for isolates with higher ploidy (>2n) was found for in the 3 phylogenetically distinct beer clades (African - 06.A, mosaic - 07.M and ale - 11.A beers, $\chi 2$ test, p-value < 4.2e-16), the mixed subpopulation (08.M) containing the baker isolates ($\chi 2$ test, p-value = 2.7e-14) and the African palm wine 13.A ($\chi 2$ test, p-value = 0.0002). For each clade, n indicates the number of isolates.

Figure S12. Fitness trait and ploidy level by conditions. For each condition, the fitness distribution was partitioned by ploidy level. Most conditions follow the general trend *i.e.* demonstrate a mitotic advantage of diploidy. Growth conditions are described in Table S2. The number above the violin plot represent the n number for each plot: centre lines, median; boxes, interquartile range (IQR); whiskers, 1.5× IQR. Data points beyond the whiskers are outliers.

Figure S13. Genome-wide distribution of aneuploidy events. a, Distribution of the aneuploidy events by chromosome. The color of the bar plots refers to the number of sub/supernumerary chromosomes. **b,** Number of aneuploidy events affecting each chromosome plotted against the size of the chromosomes. Aneuploidies are only weakly correlated with chromosomal size as previously observed in the clinical isolates[10]. r corresponds to the Pearson's correlation coefficient.

Figure S14. Aneuploidy level and subpopulations. For each clade, n indicates the number of isolates. **a,** Proportion of aneuploid isolates for each of the defined subopulations. An enrichment of aneuploid strains is observed in the sake - 25.S ($\chi 2$ test, p-value = 2.9e-08), ale beer - 11.A ($\chi 2$ test, p-value = 5.9e-06) and the mixed subpopulation containing the baker isolates - 08.M ($\chi 2$ test, p-value = 3.6e-09) subpopulations. **b,** Genome-wide distribution of the aneuploidies by clade. The color of the bar plots refers to the number of sub/supernumerary chromosomes.

Figure S15. Core and variable ORFs. a, Frequencies of core and variable ORFs related to the distance from the nearest telomere (x-axis). The horizontal dashed line indicates the whole genome percentage of dispensable ORFs. The terminal 50 kb of the chromosomes are enriched in variable ORFs. **b,** dN/dS shows an overall stronger signature of purifying selection in the variable ORFs (p-value = 5.89e-15). The essential genes have been removed to avoid bias caused by their increased frequency among the core ORFs. Center lines, median; boxes, interquartile range (IQR); whiskers, 1.5× IQR. See supplementary Table 21 for confidence intervals.

Figure S16. Origin of variable ORFs. We catalogued variable ORFs as ancestral segregating, introgressed or horizontal gene transfer (HGT) according to their phylogeny. ORFs with their closest orthologs in other *S. cerevisiae* isolates (purple circles) and consistent with genome phylogeny are defined as ancestral segregating. ORFs that show the best match with orthologs belonging to a closely related *Saccharomyces* species (red circles) were considered introgressed. ORFs having their best match with orthologs in other less related species were catalogued as HGTs.

Figure S17. Introgression from an unknown *Saccharomyces* species in the Taiwanese lineage. a, We discovered a 24 kb introgression in chromosome XI of the Taiwanese clade. The *S. cerevisiae* YKR064W - YKR078W ORFs were replaced by a highly divergent genomic segment with syntenic

ORFs. The annotation of the S288C region, the matching contigs from the AMH isolate and the annota- tion are shown. Pink background highlights the introgressed ORFs. The YLR460C gene (Chr. XII 1059757-1060887) is found at the end of a contig in all the three strains suggesting a translocation event flanking the introgressed region. Red and blue indicate the ORF orientation. Sequence similarity plots of pairwise aligned sequences show perfect synteny and high identity level in the flanking regions of the introgression. **b,** Phylogenetic analysis revealed that the introgressed DNA belongs to an unknown *Saccharomyces* species with ~13% divergence to *S. cerevisiae* and ~11% to *S. paradoxus* isolates. The ML rooted tree is based on the alignment of 14 concatenated introgressed ORFs.

Figure S18. Variation of introgressed ORFs across subpopulations. Boxplots represent the distribution of the number of introgressed ORFs per strain for each subpopulation. Enrichment was found in the alpechin (median of 257 introgressed ORFs, p-value = 2.00e-12, variance among set non-significantly different), bioethanol (median 39, p-value = 1.02e-09), Mexican agave (median 159, p-value = 1.34e-5) and French Guiana (median 61, p-value = 7.22e-16) clades. All the p-values are calculated using the two-sided Mann-Whitney-Wilcoxon test. Center lines, median; boxes, interquartile range (IQR); whiskers, 1.5× IQR. Data points beyond the whiskers are outliers. Most conditions follow the general trend *i.e.* demonstrate a mitotic advantage of diploidy. Growth conditions are described in Table S2. The number above the violin plot represent the n number for each plot. Centre lines, median; boxes, interquartile range (IQR); whiskers, 1.5× IQR. Data points beyond the whiskers are outliers. See supplementary Table 21 for confidence intervals.

Figure S19. Population landscape and ancestry of introgressed ORFs. Isolates were sorted as in the phylogenetic tree and ORFs were sorted first according to the *S. paradoxus* clade (American, European, ND not determined, NA not applicable since derived from different species), followed by number of occurrences in our collection. There is a striking correlation between the geographical origin of the S. cerevisiae clades and the ORF ancestry. Spanish Alpechin have mainly European *S. paradoxus* ORFs while Brazilian bioethanol, Mexican agave and French Guiana clades have *S. paradoxus* ORFs with American ancestry.

Figure S20. Variation of HGT ORFs across subpopulations. Boxplots represent the distribution of the number of the HTG ORFs per strain for each subpopulation. Notably, an enrichment of HGT ORFs was found in some human associated subpopulations, such as the Wine/European - 01.W, Brazilian bioethanol - 03.B, mosaic beer - 07.M and mixed origins - 08.M origins (2-sided Mann-Whitney test p-values = 8.50e-05, 2.73e-05, 1.33e-05, 8.24e-07, respectively). Above the plot, the number of isolate for each clade is reported. Center lines, median; boxes, interquartile range (IQR); whiskers, 1.5× IQR. Data points beyond the whiskers are outliers. See supplementary Table 21 for confidence intervals.

Figure S21. Global view of HGT ORFs. a, The heat map shows an overview of the presence (dark blue) of HGT ORFs in the sequenced strains. The six largest events are labelled as regions A-F and isolates are ordered according to the tree (see isolate Supplementary table 1). **b,** Patterns of ORF for the six large HGT events (regions A-F). The profiles are ordered according to the number of strain occurrences (number on the right, starting with the number of strains without the HGT event). The extent of the previously characterized regions (A-C) is indicated by coloured bars underneath. See also Supplementary note 3.

Figure S22. Origin of HGT regions. The plots show the HGT regions found in *S. cerevisiae* strains aligned to their closest hit. The identity was calculated on 1 kb windows. a-c, regions B, C and D contigs and likely donors are shown (grey boxes) and sequence identity calculated using nucmer is plotted underneath. In c, the dashed box contains the region D. This region is subtelomeric and maps to two different *Torulaspora delbrueckii* chromosomes. The region homologous to chromosome VIII maps to two different regions with the small fragment with inverted orientation (indicated in red). d, HGT region from unknown donor detected in a single South American isolate (ALI). Given the high sequence divergence with the closest matching species, we used PROmer to align the sequences.

Figure S23. Heterozygous versus homozygous isolates. a, Number of heterozygous and homozygous isolates for each clade. **b,** Proportion of heterozygous isolates for domesticated and non-domesticated subpopulations. n indicates the number of isolates.

Figure S24. Distribution of the LOH events. a, Distribution of the LOH regions within the heterozygous natural isolates across the 16 nuclear chromosomes (I to XVI). Colored regions correspond to LOH events whereas white regions represent heterozygous parts of the genomes. **b,** Distribution of the number of regions under LOH. **c,** Distribution of the number of the cumulative size of the LOH regions per genome.

Figure S25. Loss-of-heterozygosity level and subpopulations. For each clade, n indicates the number of isolates. **a,** Distribution of the number of regions under LOH per isolates. **b,** Cumulative size of the LOH regions per genome in each subpopulation. Center lines, median; boxes, interquartile range (IQR); whiskers, 1.5× IQR. Data points beyond the whiskers are outliers.

Figure S26. Heterozygous level within the *S. cerevisiae* species. a, Distribution of the number of heterozygous site per kb in the sequenced isolates. This number was determined by removing the LOH regions. **b,** Distribution of the number of heterozygous site per kb for each subpopulation. **c,** Box plots depicting the variation of the number of heterozygous sites per kb in each and between subpopulations. For each clade, n indicates the number of isolates. Center lines, median; boxes, interquartile range (IQR); whiskers, 1.5× IQR. Data points beyond the whiskers are outliers.

Figure S27. Heterozygosity and LOH level. An anti-correlation is observed between the cumulative size (**a**) as well as the number of LOH regions (95 percent confidence interval: -0.586; -0.445) (**b**) and the number of heterozygous sites per kb (95 percent confidence interval: -0.514; -0.358) across 415 heterozygous isolates. The heterozygosity level is determined after masking the LOH regions. r corresponds to the Pearson's correlation coefficient. See supplementary Table 21 for confidence intervals.

Figure S28. Patterns of genome evolution between wild and domesticated clades. a, Pairwise comparisons between couples of either domesticated (red) or wild (green) clades (n=36 pairs of domesticated clades and 55 pairs of wild clades). Comparisons of domesticated lineages have more differences in their ORFs content than comparisons of wild lineages, despite lower SNP differences. The violin plots of the distributions are shown on top for the SNPs distance (2-sided Mann-Whitney test p-value = 5.86e-07) and on the right for the number of unshared ORFs (2-sided Mann-Whitney test p-value = 8.59e-13). For all the comparisons, except the SNPs distance, the variances of the data are not significantly different. **b,** Wild clades have fewer CNVs than domesticated clades (median 275 versus 346, 2-sided Mann-Whitney test p-value = 1.6e-02), compared to both multiallelic (105 versus 127.5, 2-sided Mann-Whitney test p-value = 6.0e-03) or hemizygous ORFs (173 versus 230, 2-sided Mann-Whitney test p-value = 4.3e-02). To avoid bias due to aneuploidy or polyploidy, only euploid diploid isolates have been taken into account, although analysing all of the strains provided similar results. Center lines, median; boxes, interquartile range (IQR); whiskers, 1.5× IQR. Data points beyond the whiskers are outliers.

Figure S29. Ty distribution across subpopulation. Panels show for each clade (n indicates the number of isolates by clades) the copy number per strain of the five transposon families. There is a great deal of variation ranging from almost complete absence (*e.g.* Malaysian clade) to large copy number expansions of specific families (*e.g.* Ty1 and Ty2 in French Dairy, Ty2 in Alpechin, Ty1 in Mexican Agave, Ty3 in Ecuadorean). Center lines, median; boxes, interquartile range (IQR); whiskers, 1.5× IQR. Data points beyond the whiskers are outliers. See supplementary Table 21 for confidence intervals.

Figure S30. Linkage disequilibrium in the 1,011 genomes. LD decays to half of its maximum value around 500 bp. Estimation of LD was performed on 82,869 SNPs from 1011 individuals. r^2 is a measure of LD (see Methods).

Figure S31. Phenotypic diversity and subpopulations. Principal component analysis (PCA) using growth ratio under all phenotypic conditions as markers. Isolates are colored according to clade. The PCA analysis, performed on 971 independent individuals and 35 variables, does not divide individuals into the identified subpopulations. For each clade, n indicates the number of isolates.

Figure S32. Principal component projection (PCA) using growth ratio as in Fig. S31. Here, each graph is meant to highlight a clade against the total population. For each clade, n indicates the number of isolates.

Figure S33. Genome-wide association results in *S. cerevisiae*. Manhattan plots for all conditions that reached significance in our genome-wide association analysis. The chromosomes are represented on the x-axis from 1 to 16, and the CNVs are represented as an extra chromosome. The threshold in red is obtained with 100 permutations of the phenotypes. GWAS was performed using 971 independent individuals (82,869 SNPS and 925 CNVs).

Figure S34. Genome-wide inflation factor of the $\chi 2$ test statistics for our GWAS.

Supplementary Notes

Supplementary Note 1 - Detailed description of the *Saccharomyces cerevisiae* clades

01.W - Wine/European (n=362). The Wine/European lineage is the most sampled clade and contains the large majority of winemaking strains. We sequenced 79 strains, mainly from Italy (n=32) and Spain (n=26), isolated between 1933 and 1960 before the introduction of the wine starter practice. These strains mainly belong to the Wine/European clade and while some are very closely related, they do not form specific subclades. Nevertheless, nine strains isolated from 1933 to 1956, mainly Italian, create a tight group according to both SNP distance and ORFs content. Another 17 strains (10 with isolation date information dating back to the 1950s) also create a separate group. These strains were isolated in Spain from both natural and domesticated niches, except for a single soil isolate from Finland.

The Wine/European clade shows at least 4 major subclades (1.1-1.4, Fig. 1). Two subclades (1.2 and 1.3) are enriched for clinical strains. The subclade 1.2 (**n=13**) is characterised by extreme amplification of subtelomeric Y'-elements (median = 77, five isolates have > 150 copies) and subclade 1.3 (**n=23**) contain a group of closely related clinical strains from Europe and as well as the probiotic *S. cerevisiae* var. *boulardii* strains and Asian strains from flowers (notably also from the lychee tree, which was the primary isolation source of the probiotic strains). Wild strains represent 16% of the isolates in the Wine/European clade (n=58, majority European n=42) while another 15% (n=55) has undetermined origin. The wild isolates are almost randomly distributed across the clade.

Nevertheless, one subclade is enriched in wild strains (2-sided Mann-Whitney test p-value = 3.6e-4). This subclade contains 8 strains from wine related sources (6 from grape must, 1 from wine, and 1 from grapes), and 10 from "domesticated nature" sources, *i.e.* fir, plum, apple, apricots, and pear (trees and fruits) (**n=18**). Twelve of these eighteen harbour a partial region C (ranging from ORF 2 to 6). The most divergent subclade (1.4) is composed mainly of Georgian wine strains isolated from wine conserved in amphorae (n=39). This subclade is sufficiently divergent that ADMIXTURE described it as a separate clade (see Fig S8). Caucasus is thought to be the birthplace of winemaking (~6000 years ago) and these isolates might represent the current closest relatives to the original pool of wine domesticated *S. cerevisiae* strains.

02.A - Alpechin (n=17). These strains were mostly isolated from olive mill wastewater (alpechin) in Spain and share ancestry with the Wine/European clade. All the isolates in this lineage have a much higher quantity of dispensable material compared to all the other lineages. This dispensable ORFs abundance is due to massive introgression events of ~400 ORFs per strain originating from *S. paradoxus*. The frequencies of shared introgressed ORFs is high, for example 244 ORFs are shared by at least half of the isolates while at least 112 are common to all the 17 strains.

M1.M - Mosaic region 1 (n=17). This group consists of strains with various origins including 8 isolates from French cider and 2 wild isolates. Both the isolated branches and the ADMIXTURE results indicate mosaicism in concordance with the DAPC data. In contrast with the known mosaic strains, these isolates do not seem to derive from a large number of clades but are mostly related to the wine strains with a minor ancestry component shared with the Brazilian bioethanol clade and the other mosaic groups.

03.B - Brazilian bioethanol (n= 35). This is a loose cluster of strains isolated in Brazil from sugar cane products, bioethanol sugar mills and cachaça liquor. There is an internal tight cluster with increased CN of the RDN unit (median 126 copies, compared to 109 of the whole bioethanol clade and 96 for the entire sequenced collection). This cluster shows abundant presence of American *S. paradoxus* introgression. Analysis based on similarity plots and ADMIXTURE results suggest that the Brazilian bioethanol ancestry component is shared, to different degrees, with all the mosaic groups of the tree.

04.M - Mediterranean oak (n=8). A tight cluster of wild strains related to the recently described and well characterised European wild population. Our strains belonging to this clade extend the known distribution of this subpopulation up to the Caucasian region at the border of Europe and Asia. Despite their SNP genetic distance, this clade is highly similar in genome content to the other natural strains from Asia and the Americas at the bottom of the phylogenetic tree. Those similarities are also captured by the DAPC analysis on the genomic content difference matrix, which create a single cluster for all these strains (Fig. S7).

05.F - French dairy (n=32). This clade mostly consists of strains isolated from French cheeses and shows a considerable amount of intra-clade variation both in terms of SNPs and genome content. A peculiarity of the genomes belonging to this clade is that they have the lowest number of unique ORFs (median 5967.5) compared to any other clade. These strains show better fitness when grown using galactose as carbon source and harbour a multi-allelic DLD3 (D-Lactate-dehydrogenase) gene (copy number =3). Phylogenetic analyses suggest that this clade is a clean lineage, although very closely related to the African beer lineage. This lineage harbours the largest copy number (CN) of transposable elements (median CV is 56.75 ranging from 29 to 79) driven by drastic expansion of the Ty1 and Ty2 classes.

06.A - African beer (n=20). This lineage is characterised by high ploidy. It is one of the two clades with a median ploidy greater than 2 (median 3.5, max 5). South African Kaffir beer strains have variable ploidy (1, 2 or 3) while bili bili (Chad) strains have ploidies of 3 or 4. The highest ploidy is 5 among the pearl millet beer isolates from Ivory Coast. This clade also includes 3 African bakery strains (all diploids) that form a small subgroup and 3 natural strains (two from the mainland are tetraploid, one from Madagascar is diploid). The production of these African beverages does not rely on the use of commercial starter strains but occur by spontaneous fermentation. The lineage appears to have been through a strong process of specialization,

losing several genes and becoming the lineage with the second lowest number of unique ORFs (median 5973). ADMIXTURE results suggest that this is a clean lineage.

07.M - Mosaic beer (n=21). This group consists mainly of beer strains but also includes a few strains with different ecological origins. Both the DAPC and ADMIXTURE analyses suggest this is a mosaic clade with shared ancestry common to several other mosaic strains, especially those belonging to the group M3 and similar to the ones from Brazilian bioethanol and other mosaic groups M1 and M2. The ploidy in this clade is low compared to the other two beer clusters (median 2). The clade was already described previously[1] (see Beer 2 clade) but its mosaic component was not evident.

M2.M - Mosaic region 2 (n=20). More than half (n=12) of these mosaic single-branch strains are natural isolates and include a cluster of strains isolated from the Israeli Evolution Canyon (n=8) and a smaller cluster of cider isolates (n=3). Both PCA and ADMIXTURE indicate that all isolates are mosaic.

08.M - Mixed origin (n=72). A large cluster of isolates isolated from a large array of ecological sources. This group includes the majority of the baking isolates (n=23 out of 38) as well as clinical (11), and wild isolates (26). A total of 10% of the beer strains in our collection belong to this clade (6 out of 59). ADMIXTURE indicates largely a clean clade closely related to the Ale beer lineage. The clade has been described previously[1].

09.M - Mexican agave (n=7). These strains were isolated from artisanal Agave fermentation for Mezcal production in Tamaulipas, Mexico. We detected in this group the highest number of unique ORFs per genome due to a massive amount *S. paradoxus* introgressions largely maintained in a heterozygous state. The ADMIXTURE results suggest that they originated from an outcross between Wine/European and French Guiana strains but we were unable to conclusively confirm this with additional SNP analysis approaches.

10.F - French Guiana human (n=31). These strains were isolated from a remote village of Wayampi Amerindians in French Guiana. Peculiarly, these strains were mostly isolated from stool of healthy people and few from plants and animals associated with this indigenous human population. This clade also has a large number of introgressed *S. paradoxus* genomic blocks driving a large genome content difference. SNP analysis using ADMIXTURE suggest a clean lineage without contribution from other ancestries.

11.A - Ale beer (n=18). Previously identified (see Beer 1 clade)[1], this clade consists of several European strains mainly from the UK and Benelux and several of them are used in Ale beer production. This clade has the highest ploidy (median 4). In contrast to the mosaic beer clade, this group seems to be a nearly clean lineage, considering both genome content and SNPs, although ADMIXTURE indicate a possible relationship with the Mixed origin clade.

12.W - West African cocoa (n=13). A small tight clade formed by strains isolated from cocoa seed fermentation. Genome content analysis places this clade as the closest to the Mediterranean oak clade. This is consistent with the cocoa fermentation process that rarely uses starter cultures and instead relies on wild populations, which makes uncertain to catalogue these strains as domesticated. ADMIXTURE analysis, also consistent with a recent report[2], suggested signs of mixtures with European (Wine/European) and Asian (Far East Asia) lineages, but also similarities with North American and African palm wine.

M3.M - Mosaic region 3 (n=113). This group of strains has very variable geographic and ecological origins and has been partially described in previous population genomics surveys. Our data confirm the high mosaicism of these isolates with multiple lineages involved. Neither in terms of genome content nor SNP distance, the isolates in this group share enough similarity to cluster into a clade. Different sources of mosaic ancestry can be identified via the ADMIXTURE results. A small subgroup of 17 strains is separated from the main group by the West African cocoa fermentation lineage. This minor cluster has a major bioethanol component, which deceases in frequency in a subgroup of three strains and is replaced by Wine/European and African palm wine components. A large group of 96 strains (23 clinical) show an abundant Wine/European component that decrease and is gradually replaced by a Sake component following the strain order along the phylogenetic tree backbone**.** DAPC on genomic content analyses confirm the similarities between subset of these isolates and the other mosaic groups, the bioethanol, the mosaic beer and the Wine/European clades.

13.A - African palm wine (n=28). This clean lineage includes strains isolated from palm wine production, typical of several African countries. Previously this clade was identified as West African[3], but we have found that its geographical distribution reaches also the eastern coast of the African continent. However, strains from different countries generate distinct geographic subclades (Djibouti, Ivory Coast and Nigeria) with the East African and West African that separate into two main sublineages. ADMIXTURE data seems to indicate that the East African subclade has North American ancestry in addition to the African palm wine main component.

14.C to 16.C - Wild Chinese lineages. These strains are representative of a large strain collection of highly diverged lineages isolated from primeval forest in China[4]. CHN I (n=1) was isolated on the Bawangling Mountain in the island of Hainan and represented the most divergent known *S. cerevisiae* lineage. CHN II (n=2) strains were isolated from the Qinling Mountain in Shaanxi region and CHN III (n=2) strains were found on Wuzhi Mountain in Hainan.

17.T - Taiwanese (n=3). These strains were isolated from Taiwanese forest soil. They now represent the most diverged *S. cerevisiae* clade ever described. This lineage has an average difference of 1.1% in

nucleotide composition compared to the other isolates, which is more than twice the typical distance between different clades (0.5%) and exceed the divergence of CHN I (0.8%), which was hereto the most divergent known lineage.

18.F - Far East Asia (CHN IV) (n=9). This cluster of wild strains is related to other Asian lineages and harbours a group of Far East Russian, Japanese and CHN IV[4] strains (from Beijing).

19.M - Malaysian (n=6). These strains were isolated in Malaysia, mainly from the nectar of Bertam palms. This lineage is characterised by major chromosomal rearrangements that cause the strong post-zygotic reproductive isolation barrier. This clade harbours the lowest number of transposable elements (the median Ty CN is 1, ranging from 1 to 3).

20.C - CHN V (n=2). These isolates were isolated from the primeval forest on the Wuzhi Mountain on the Chinese island of Hainan[4].

21.E - Ecuadorean (n=10). This group of natural wild strains from flowers or insects came from the Yasuni National Park in the Orellana province of Ecuador. ADMIXTURE models this lineage as pure and tightly connected to neighbouring clades such as the Malaysian, North American, Far East Asia and especially CHN-V. A unique feature of this clade is the complete absence of the subtelomeric Y' elements.

22.F - Far East Russian (n=4). A group of close strains from Blagoveshchensk, a Russian city at the border with China, related to the Malaysian and the Ecuadorean clades.

23.N - North American oak (n=13). This is a tight clade (almost a clonal expansion) of wild strains from woodland in Pennsylvania. These North American strains show strong similarities with phylogenetically related strains from Japan and Taiwan.

24.A - Asian islands (n=11). All isolates belonging to this clade are Asian except one. Eight strains have been isolated on several Asian islands from insular states (Philippines, Indonesia and Sri Lanka) and include both wild and domesticated origins. ADMIXTURE assigns to these strains the component shared by the North American clade but with some minor contribution from the Sake clade component.

25.S - Sake (n=47). This cluster is a tight group of Japanese sake strains and 23 strains form an extremely thigh subclade. The sake clade is closely related to the neighbouring Asian fermentation clade and they branched off from the same phylogenetic lineage. Sake yeast strains were generated by the Brewing Society of Japan and the National Research Institute of Brewing in middle of the 1900s. Most of the sake isolates originated from the Kyokai no. 7 lineage, derived from four established strains (K6, K7, K9, and K10).

Isolation of mutants (mutation breeding) and generation of hybrids (cross breeding) were the approaches used to generate isolates for sake production. Only a limited number of individuals were used during domestication leading to the observed low level of genetic diversity.

Interestingly, one Japanese sake strain (CMG) carries 14 ORFs introgressed from *S. mikatae*, consistent with their geographic overlap. This event represents the only largest example of introgressions in addition to the one reported from *S. paradoxus*. It is interesting to note that *S. mikatae* is the next closely related species after *S. paradoxus*, perhaps suggesting that the level of sequence divergence play a role in the generation or fate of genomic introgressions.

26.A - Asian fermentation (n= 39). Many strains have South-Eastern Asia (n=17) origin, but other Asian, American, European and African strains are also present. There is an internal tight cluster of 10 strains from Ecuador (n=6), Asia, Africa and Europe. The ecological origins are variable and include 8 rice related strains (4 from rice wine and 4 from other rice fermentations). This clade is a clean lineage closely related to the Sake clade for both genome content and SNPs distance.

Supplementary Note 2 - The *Saccharomyces cerevisiae* natural plasmid

The 2μ *S. cerevisiae* plasmid is widely present in the collection (653 isolate out of 1,011, Supplementary fig. 9). Based on sequence, three classes of plasmids were previously described[5] and we obtained relative frequencies in the population. Class A is the most common form (n=463) and it is also present in the reference strain S288C, while class C is a much less common form (n=26). A recombinant form, called Class B (n=171), is known to have the *FLP1* and *REP2* genes from the Class A and *RAF1* and *REP* genes from Class C. In addition of these classes previously characterised, we identified some extremely rare variants. The Class B* is found only in one isolate and it is the complementary recombinant form of class B, having *RAF1* and *REP* from Class A and *FLP1* and *REP2* from Class C, perhaps derived from the same recombination event that generated the Class B type. We detected the *S. paradoxus* 2μ type (Class P) in three isolates. The Class A, C and P sequences have pairwise difference of ~ 10% (A vs. C 8%, A *vs.* P 10%, C *vs.* P 11%. In the three highly divergent Taiwanese isolates, we identified an extremely divergent plasmid (Class D) with a divergence of about 20% compared to all the *S. cerevisiae* and *S. paradoxus* classes. We compared these four plasmid types with the *S. eubayanus*[6] plasmid, which has a sequence divergence of 28% from the other classes. These results are consistent with the form D originated from an introgression from closely related species (e.g. *S. mikatae*, *S. arboricolus* or *S. kudriavzevii* for which the plasmid sequence is unavailable) or from a new undescribed species. It is worth noting that the Taiwanese lineage also harbour an introgression of 14 ORFs from an unknown species (Supplementary fig. 17**)**, perhaps the same that also has transferred the 2μ plasmid. We have used the sequence coverage to infer the plasmid copy numbers and revealed extreme variation across isolates and plasmid type (Supplementary fig. 9**)**.

Supplementary Note 3 - Detailed description of introgression and horizontal gene transfer events

In total, 913 introgressed ORFs were identified with 885 coming from *S. paradoxus*. Interestingly, introgressed ORFs are rare in the highly diverged lineages, consistent with secondary contacts with *S. paradoxus* occurring mainly after the out-of-China dispersal (Fig. 2). No ORFs can be traced to *S. kudriavzevii* or *S. eubayanus*, despite hybrids with *S. cerevisiae* being frequently described. These results imply that the lack of introgression from more divergent species is either not occurring because of higher sequence divergence or is selected against because of biological incompatibilities.

The amount of introgressed content is highly variable between the different clades (Supplementary fig. 18). Massive enrichment was found in the alpechin, bioethanol, Mexican agave and French Guiana subpopulations (two-sided Mann-Whitney-Wilcoxon test p-value 9.46e-46), *i.e.* human associated niches where the two species might coexist and consequently represent interspecific hybrid zones. As mentioned, there is a striking match between the geographic origins of the four *S. cerevisiae* clades and the ancestry of *S. paradoxus* introgressed ORFs (Supplementary fig. 19). In fact, introgressions from the American *S. paradoxus* subpopulation were found in the French Guiana, Brazilian bioethanol and Mexican agave clades whereas the alpechin lineage, mainly isolated from Europe, carries introgressions from the European *S. paradoxus* subpopulation.

We also identified 6 large HGT events (regions A-F) that account for a total size of ~500 kb, with 3 events of ~150 kb partially characterized previously (A, B and C regions) (Supplementary fig. 21a, Supplementary fig. 22, Supplementary table 6)[7]. One striking finding is that large HGT events are maintained in very few strains, whereas smaller fragments from the same HGT event are more abundant in the population. The distributions of the ORFs in the A-F regions show complex patterns of presence/absence, which is compatible with multiple reductions of the large ancestral HGT events (Supplementary fig. 21b).

The region A was detected in 44 strains, 36 of which belong to the Wine/European clade. In addition, a smaller version lacking the terminal two ORFs was found in two additional strains (CHE and CHF).

For the region B acquired from *Zygosaccharomyces bailii* sensu lato, the pattern is more complex. We detected a single strain (BMD) that harbours an extremely large event (117 kb versus the 17 kb originally described) with at least 22 additional ORFs. These additional ORFs are syntenic to a region present in *Z. bailii* on the chromosome II (Supplementary fig. 22a) and are contiguous in the BMD *de novo* assembly, supporting that this extended region B represents the ancestral event before size reduction by multiple independent deletions. The 5 ORFs originally described are the most common in the population but additional ORFs from the ancestral event can be retained. Remnants of the ancient region B are present in 575 strains: 166 with only one ORFs and they are not restricted to the Wine/European clade.

A similar scenario is observed for the 65 kb region C transferred from *Torulaspora microellipsoides* and initially described in EC1118[7]. We collected multiple evidences indicating a larger ancestral event followed

by deletions leading to size reduction. A single strain isolated from the Carlsberg brewery contains the long ancestral event of ~165 kb with at least 41 ORFs. Small relics of this larger event are detected in 186 additional strains. Recently, two strains were described carrying ORFs located at the two extremities of the ancestral event we detected (Supplementary fig. 22b)[8].

The three newly identified regions (D, E, F) all show clear signs of size reductions (Supplementary fig. 21). The region D that consist of 16 ORFs of 54 kb with high sequence identity (~95%) with *Torulaspora delbrueckii*. This block is syntenic to *T. delbrueckii* chromosome 3, except for an extremity, which aligns to the right subtelomere of chromosome 8 (Supplementary fig. 22c). The region E (17 ORFs 70 kb) and region F (10 ORFs 50 kb) could not be traced to a clear donor yeast species, although the best identities matches are with *Saccharomycetaceae* genera. Out of the three strains in which the region E has been identified, one of them lack of three terminal ORFs, while four out of six strain containing the region F lack five terminal ORFs (Supplementary fig. 21b).

We also identify at least 46 ORFs found isolated or in very small clusters in single isolates, which we refer to as candidate HGT events. We detected an event encompassing 6 ORFs in a South American isolate (ALI). These ORFs show a best match with *Lachancea thermostabilis* with ~60% identity (Supplementary fig. 22d). In addition to the HGT events coming from yeast species, a handful of inter-kingdom HGT have been detected. Two ORFs from bacteria were previously characterized in the S288C reference, these ORF are *YLR157C*, present in 88 strains and *YOL164W*, present in 114 strains. In addition, we found 3 ORFs with likely bacteria origin (found in A, 27 and 228 strains) and one ORF found in 3 strains which is related to the viral yeast killer protein M28 found in *S. paradoxus* (protein identity of 82%), which is known to be integrated in some isolates conferring a killer phenotype. These three strains belong to separate clades (African beer, mosaic and African palm wine) but share the African origin.

Supplementary Note 4 - Timing estimation of major events in the evolutionary history of yeast

Given no well-defined fossil record is available for yeasts to be used for calibration-based methods, we performed our molecular dating analysis using a molecular-clock-based method based on previous studies[9,10]. These previous studies used synonymous substitution sites as a proxy for sequences under neutral evolution and infer the divergence time between different strains accordingly by assuming a strict molecular clock. Here, we restricted the analysis to the 4-fold degenerated (4D) sites for the calculation to further minimize the confounding effect introduced by natural selection (*e.g.* selection for biased codon usage). Based on the 41-way CDS alignments that we constructed for the phylogenetic analysis showed in Figure 2a, a total of 160,415 (denoted as N here) 4D sites were extracted. If one were to suppose the total number of different 4D-sites between strain A and strain B is m, the expected neutral evolution distance with Jukes-Cantor correction (djc) can be calculated as: $d_{jc} = -3/4 \log(1-4/3 \cdot m/N)$. Assuming a maximal year-round generation number of G = 2,920 (*i.e.* 2,920 generation per year or 8 generations per day)[9] and two

independent estimates[9,11] of spontaneous mutation rates per base pair (bp) per generation, $\mu = 1.84 \times 10^{-10}$ and $\mu = 1.67 \times 10^{-10}$, we can estimate the minimal bound for the divergence time between strain A and strain B using the formula: T = (djc/u)/G. In this way, we obtained tentative estimates for the timing of the *S. cerevisiae* - *S. paradoxus* speciation, *S. cerevisiae* out-of-China and different *S. cerevisiae* domestication events as follows. Our estimates regarding to the evolutionary history of the sake and wine lineages are consistent with previous estimates using different datasets[9,10]. More precise estimates could be obtained in the future when lineage-specific generation time and mutation rate data become available.

Events	Strain pair based for the calculation	Years	
		$\mu=1.84 \times 10^{-10}$	$\mu=1.67 \times 10^{-10}$
S. cerevisiae - *S. paradoxus* speciation	CBS432 vs. S288C	292,983	322,808
S. cerevisiae out-of-China	BAM vs. S288C	14,295	15,750
Sake *S. cerevisiae* domestication	ADQ vs. Y12	3,915	4,314
Wine *S. cerevisiae* domestication	ADS vs. DBVPG6765	1,411	1,555
The divergence time between the wine and sake *S. cerevisiae* lineages	Y12 vs. DBVPG6765	12,562	13,841

References

1. Gallone, B. *et al*. Domestication and divergence of *Saccharomyces cerevisiae* beer yeasts. *Cell* **166**, 1397–1410 (2016)
2. Ludlow, C.L. *et al*. Independent origins of yeast associated with coffee and cacao fermentation. *Curr Biol.* **26**, 965-971 (2016)
3. Liti, G., Carter, D. *et al.* Population genomics of domestic and wild yeasts. *Nature* **458**, 337–341 (2009)
4. Wang, Q.M., Liu, W.Q., Liti, G., Wang, S. A. & Bai, F. Y. Surprisingly diverged populations of *Saccharomyces cerevisiae* in natural environments remote from human activity. *Mol Ecol.* **21**, 5404–17 (2012)
5. Strope, P.K. *et al*. The 100-genomes strains, an *S. cerevisiae* resource that illuminates its natural phenotypic and genotypic variation and emergence as an opportunistic pathogen. *Genome Res.* **25**, 762–774 (2015)
6. Baker, E. *et al.* The genome sequence of *Saccharomyces eubayanus* and the domestication of lager-brewing yeasts. *Mol Biol Evol.* **32**, 2818–31 (2015)
7. Novo, M. *et al*. Eukaryote-to-eukaryote gene transfer events revealed by the genome sequence of the wine yeast *Saccharomyces cerevisiae* EC1118. *Proc. Natl Acad. Sci. USA* **106**, 16333–8 (2009)
8. Marsit, S. *et al.* Evolutionary advantage conferred by an eukaryote-to-eukaryote gene transfer event in wine yeasts. *Mol. Biol. Evol.* **32**, 1695–707 (2015)

9. Fay, J. C. & Benavides, J. A. Evidence for domesticated and wild populations of *Saccharomyces cerevisiae*. *PLoS Genet.* **1**, 66–71 (2005)
10. Liti, G., Barton, D. B. & Louis, E. J. Sequence diversity, reproductive isolation and species concepts in *Saccharomyces*. *Genetics* **174**, 839–50 (2006)
11. Zhu, Y.O. *et al*. Precise estimates of mutation rate and spectrum in yeast. *Proc Natl Acad Sci U S A*. **111**, E2310–8 (2014)
