# Supplementary material for: Genome evolution across 1,011 Saccharomyces cerevisiae isolates
Source: Nature. 2018 Apr 11;556(7701):339–44. doi: 10.1038/s41586-018-0030-5 (PMC6784862; doi:10.1038/s41586-018-0030-5)
Supplement: Supplementary file 2 — Reporting Summary [file 41586_2018_30_MOESM2_ESM.pdf]

nature research

Corresponding author(s): Joseph Schacherer, Gianni Liti

☐ Initial submission ☐ Revised version ☒ Final submission

Life Sciences Reporting Summary

Nature Research wishes to improve the reproducibility of the work that we publish. This form is intended for publication with all accepted life science papers and provides structure for consistency and transparency in reporting. Every life science submission will use this form; some list items might not apply to an individual manuscript, but all fields must be completed for clarity.

For further information on the points included in this form, see Reporting Life Sciences Research. For further information on Nature Research policies, including our data availability policy, see Authors & Referees and the Editorial Policy Checklist.

Experimental design

1. Sample size

Describe how sample size was determined.	No statistical methods were used to predetermine sample sizes.

2. Data exclusions

Describe any data exclusions.	- Ploidy and level of aneuploidy: only strains in their natural state were considered for these analysis. We consequently excluded strains that were genetically manipulated.<br>- Pangenome: The CDH and CFH strains were excluded from this analysis due to the presence of Staphylococcus epidermis contamination,<br>- SNPs with a minor allele frequency (MAF) lower than 5% were excluded from the linkage disequilibrium analysis, as well as from the genome-wide association studies,<br>- SNPs with missing genotypes in more than 11 individuals were excluded from the linkage disequilibrium analysis, as well as from the genome-wide association studies,<br>- Core and variable ORFs: essential genes were excluded from the dN/dS comparison (Fig S14)<br>- CNVs between wild and domesticated clades: only euploid diploid isolates were considered for this analysis (Fig S27). We consequently excluded aneuploid strains as well as haploid and polyploid strains.

3. Replication

Describe whether the experimental findings were reliably reproduced.	Experimental replication was not attempted in our study.

4. Randomization

Describe how samples/organisms/participants were allocated into experimental groups.	Samples were not randomized for the experiments.

5. Blinding

Describe whether the investigators were blinded to group allocation during data collection and/or analysis.	Blinding was not used during data collection.

Note: all studies involving animals and/or human research participants must disclose whether blinding and randomization were used.

6. Statistical parameters

For all figures and tables that use statistical methods, confirm that the following items are present in relevant figure legends (or in the Methods section if additional space is needed).

n/a	Confirmed	
☐	☒	The exact sample size (*n*) for each experimental group/condition, given as a discrete number and unit of measurement (animals, litters, cultures, etc.)
☒	☐	A description of how samples were collected, noting whether measurements were taken from distinct samples or whether the same sample was measured repeatedly
☒	☐	A statement indicating how many times each experiment was replicated
☐	☒	The statistical test(s) used and whether they are one- or two-sided (note: only common tests should be described solely by name; more complex techniques should be described in the Methods section)
☒	☐	A description of any assumptions or corrections, such as an adjustment for multiple comparisons
☐	☒	The test results (e.g. *P* values) given as exact values whenever possible and with confidence intervals noted
☐	☒	A clear description of statistics including central tendency (e.g. median, mean) and variation (e.g. standard deviation, interquartile range)
☒	☐	Clearly defined error bars

See the web collection on statistics for biologists for further resources and guidance.

Software

Policy information about availability of computer code

7. Software

Describe the software used to analyze the data in this study.	Abyss (v1.5.2), ADMIXTURE (v.1.23), ASTRAL (v4.7.12), BLAST package (v2.2.28+), bwa (v0.7.4-r385), condetri v2.2, EIGENSOFT (v6.0.1), FastX toolkit (v0.0.13.2), FaST-LMM (v2.07), FigTree (v1.4.2), FlowJo, GATK (v3.3-0),Maker (v2.31.8), MUSCLE (v3.8.1551), PAL2NAL (v14), PAML (v4.8), Picard-tools (v1.124), Plink (v2.0), Proteinortho (v5.15), RATT, RAxML (v8.2.6), SIFT (v5.2.2), SnpEff (v4.1), SOAP (v2.21), Variscan (v2.0), vcftools (v0.1.12), YGAP.<br>R packages: adegenet(v2.01), ape(v3.4), densityClust(v0.2.1), flowCore(v1.32.2), flowViz(v1.30.1), gitter(v1.1.1), SNPrelate(v1.0.1)<br>The custom codes can be accessed via the following links:<br>http://1002genomes.u-strasbg.fr/files/<br>http://www.genoscope.cns.fr/fastxtend/

For manuscripts utilizing custom algorithms or software that are central to the paper but not yet described in the published literature, software must be made available to editors and reviewers upon request. We strongly encourage code deposition in a community repository (e.g. GitHub). *Nature Methods* guidance for providing algorithms and software for publication provides further information on this topic.

Materials and reagents

Policy information about availability of materials

8. Materials availability

Indicate whether there are restrictions on availability of unique materials or if these materials are only available for distribution by a for-profit company.	All the strains are available on request except for 11 isolates, which cannot be distributed.

9. Antibodies

Describe the antibodies used and how they were validated for use in the system under study (i.e. assay and species).	n/a

10. Eukaryotic cell lines

a. State the source of each eukaryotic cell line used.	n/a
b. Describe the method of cell line authentication used.	n/a
c. Report whether the cell lines were tested for mycoplasma contamination.	n/a
d. If any of the cell lines used are listed in the database of commonly misidentified cell lines maintained by ICLAC, provide a scientific rationale for their use.	n/a

Animals and human research participants

Policy information about studies involving animals; when reporting animal research, follow the ARRIVE guidelines

11. Description of research animals

Provide details on animals and/or animal-derived materials used in the study.	n/a

Policy information about studies involving human research participants

12. Description of human research participants

Describe the covariate-relevant population characteristics of the human research participants.	n/a

nature research

Corresponding author(s): Joseph Schacherer, Gianni Liti

☐ Initial submission ☐ Revised version ☒ Final submission

Flow Cytometry Reporting Summary

Form fields will expand as needed. Please do not leave fields blank.

Data presentation

For all flow cytometry data, confirm that:

- ☐ 1. The axis labels state the marker and fluorochrome used (e.g. CD4-FITC).
- ☐ 2. The axis scales are clearly visible. Include numbers along axes only for bottom left plot of group (a 'group' is an analysis of identical markers).
- ☐ 3. All plots are contour plots with outliers or pseudocolor plots.
- ☐ 4. A numerical value for number of cells or percentage (with statistics) is provided.

Methodological details

5. Describe the sample preparation.	Cells were first pulled out from glycerol stocks in liquid YPD in 96 well plates (30°C, overnight). 5 µL of the culture were transferred into 195 µL of fresh YPD and incubated 8 hours at 30°C. Then, 3 µL were taken and resuspended in 100 µL of cold 70% ethanol. Cells were fixed overnight at 4°C, washed twice with PBS, resuspended in 100 µL of staining solution (15 µM PI [Sigma-Aldrich], 100 µg/mL RNase A (Sigma-Aldrich), 0,1% v/v Triton-X, in PBS) and finally incubated 3 hours at 37°C in the dark.
6. Identify the instrument used for data collection.	FACS-Calibur flow cytometer using the HTS module for processing 96 well plates
7. Describe the software used to collect and analyze the flow cytometry data.	FlowJo and R packages FlowViz, flowCore, and densityClust
8. Describe the abundance of the relevant cell populations within post-sort fractions.	no sorting applied
9. Describe the gating strategy used.	no gate used

Tick this box to confirm that a figure exemplifying the gating strategy is provided in the Supplementary Information. ☐
